# Exploring Probenecid Derived 1,3,4-Oxadiazole-Phthalimide Hybrid as α-Amylase Inhibitor: Synthesis, Structural Investigation, and Molecular Modeling

**DOI:** 10.3390/ph16030424

**Published:** 2023-03-10

**Authors:** Bilal Ahmad Khan, Syeda Shamila Hamdani, Muhammad Khalid, Muhammad Ashfaq, Khurram Shahzad Munawar, Muhammad Nawaz Tahir, Ataualpa A. C. Braga, Ahmed M. Shawky, Alaa M. Alqahtani, Mohammed A. S. Abourehab, Gamal A. Gabr, Mahmoud A. A. Ibrahim, Peter A. Sidhom

**Affiliations:** 1 Department of Chemistry, The University of Azad Jammu and Kashmir, Muzaffarabad 13100, Pakistan; 2 Institute of Chemistry, Khwaja Fareed University of Engineering & Information Technology, Rahim Yar Khan 64200, Pakistan; 3 Centre for Theoretical and Computational Research, Khwaja Fareed University of Engineering & Information Technology, Rahim Yar Khan 64200, Pakistan; 4 Department of Physics, University of Sargodha, Punjab 40100, Pakistan; 5 Institute of Chemistry, University of Sargodha, Sargodha 40100, Pakistan; 6 Department of Chemistry, University of Mianwali, Mianwali 42200, Pakistan; 7 Departamento de Química Fundamental, Instituto de Química, Universidade de São Paulo, Av. Prof. Lineu Prestes 748, São Paulo 05508-000, Brazil; 8 Science and Technology Unit (STU), Umm Al-Qura University, Makkah 21955, Saudi Arabia; 9 Pharmaceutical Chemistry Department, College of Pharmacy, Umm Al-Qura University, Makkah 21955, Saudi Arabia; 10 Department of Pharmaceutics, College of Pharmacy, Umm Al-Qura University, Makkah 21955, Saudi Arabia; 11 Department of Pharmacology and Toxicology, College of Pharmacy, Prince Sattam Bin Abdulaziz University, Al-Kharj 11942, Saudi Arabia; 12 Agricultural Genetic Engineering Research Institute (AGERI), Agricultural Research Center, Giza 12619, Egypt; 13 Computational Chemistry Laboratory, Chemistry Department, Faculty of Science, Minia University, Minia 61519, Egypt; 14 School of Health Sciences, University of KwaZulu-Natal, Durban 4000, South Africa; 15 Department of Pharmaceutical Chemistry, Faculty of Pharmacy, Tanta University, Tanta 31527, Egypt

**Keywords:** oxadiazole, α-amylase inhibition, X-ray diffraction, molecular docking, DFT calculations

## Abstract

1,3,4-Oxadiazole moiety is a crucial pharmacophore in many biologically active compounds. In a typical synthesis, probenecid was subjected to a sequence of reactions to obtain a 1,3,4-oxadiazole–phthalimide hybrid (**PESMP**) in high yields. The NMR (^1^H and ^13^C) spectroscopic analysis initially confirmed the structure of **PESMP**. Further spectral aspects were validated based on a single-crystal XRD analysis. Experimental findings were confirmed afterwards by executing a Hirshfeld surface (HS) analysis and quantum mechanical computations. The HS analysis showed the role of the π⋯π stacking interactions in **PESMP**. **PESMP** was found to have a high stability and lower reactivity in terms of global reactivity parameters. α-Amylase inhibition studies revealed that the **PESMP** was a good inhibitor of α-amylase with an s value of 10.60 ± 0.16 μg/mL compared with that of standard acarbose (IC_50_ = 8.80 ± 0.21 μg/mL). Molecular docking was also utilized to reveal the binding pose and features of **PESMP** against the α-amylase enzyme. Via docking computations, the high potency of **PESMP** and acarbose towards the α-amylase enzyme was unveiled and confirmed by docking scores of −7.4 and −9.4 kcal/mol, respectively. These findings shine a new light on the potential of **PESMP** compounds as α-amylase inhibitors.

## 1. Introduction

**Figure 1:**
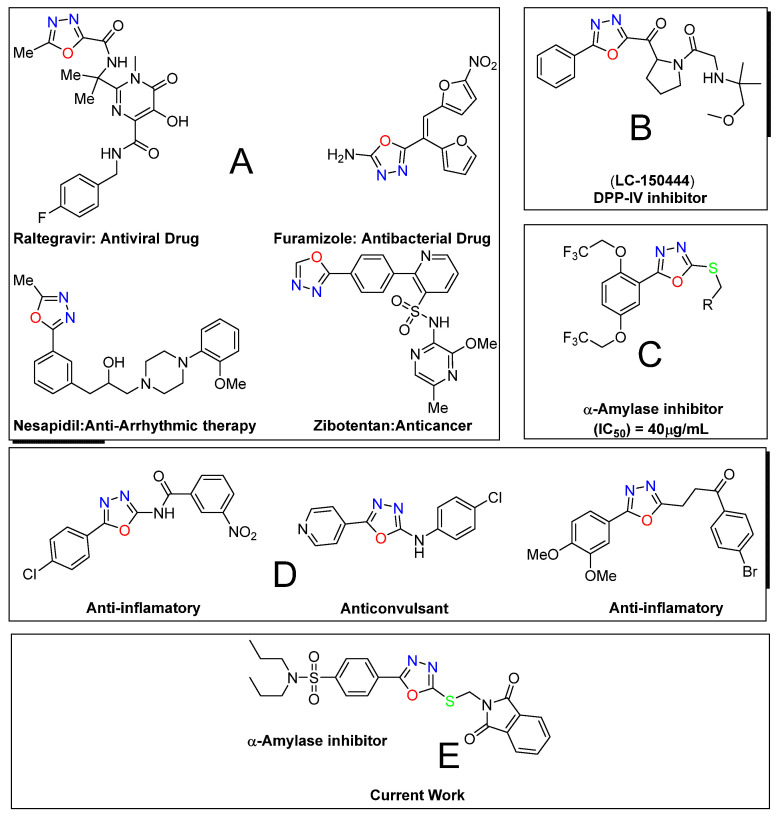
Schematic representation of the 1,3,4-oxadiazole moiety: (**A**) in commercial drugs; (**B**) in pre-clinical trials; (**C**) as α-amylase inhibitor; (**D**) in versatile biological activities; and (**E**) in current work.

Diabetes mellitus is a chronic disease aroused by the inability of the body to consume blood sugar, which leads to high blood sugar levels and the malfunction of the vascular system, vision, urinary system, and nerves. According to a WHO report, about 422 million people all around the globe are suffering from diabetes mellitus and 1.5 million/year deaths are caused by it; an ascending trend in these numbers has been continuously observed [1,2]. α-Amylase is released by the salivary glands and pancreas and catalyzes the breakdown of starch and polysaccharides to produce sugars as end-products. The excessive production of this enzyme causes an accelerated degradation of polysaccharides, resulting in an extreme sugar concentration in the blood. One way to control diabetes mellitus is to control the release of α-amylase in the blood, thus producing a restricted amount of sugars [3]. 1,3,4-Oxadiazole, a five-membered heterocycle moiety with two nitrogen atoms and one oxygen atom in the cycle, has been recognized to have a decreased aromatic and improved diene character [4]. Compounds relevant to this category of heterocycles have expressed a versatile therapeutic potential as antimicrobial [5], antiepileptic [6], anticancer [7,8], hypoglycemic [9,10], antiviral [11], and anti-inflammatory agents (Figure 1) [12]. Many commercially available drugs bear the 1,3,4-oxadiazole moiety in their core structure as a pharmacophore (Figure 1) [13]. Among those drugs, raltegravir is used to treat HIV [14], furamizole treats microbial infections [15], nesapidil is prescribed as a vasodilator [16], and zibotentan is an anticancer drug [17].

The developments in the synthesis of 1,3,4-oxadiazole scaffolds and their biological activities have been reviewed [18,19]. Probenecid, a sulfonamide containing benzoic acid, is a commercially available drug used to manage hyperuricemia [20]. Probenecid-derived amide linkage-containing structures have been successfully synthesized and analyzed for their carbonic anhydrase inhibition [21]. A little less attention has been devoted to the conversion of probenecid into its oxadiazole derivatives and investigations of their biological activities. However, probenecid-derived 1,3,4-oxadiazole–phthalimide hybrids have been investigated for their dengue virus NS2B/NS3 protease inhibition [10]. Moreover, phthalimide-based 1,3,4-oxadiazoles have been found to be anticonvulsant [22], cytotoxic [23], and peripheral analgesic [24] agents. In the context of our previous research [10,25], we envisioned that a combination of probenecid, 1,3,4-oxadiazole, and phthalimide moieties to access a hybrid molecule might result in enhancing enzyme inhibitions. Thus, a synthesis of enzyme inhibitors was undertaken, along with a single-crystal analysis, DFT exploration, and α-amylase inhibition studies of probenecid-derived 1,3,4-oxadiazole–phthalimide hybrids. Docking predictions were carried out to unveil the docking pose and score of **PESMP** against the α-amylase enzyme. These findings shine new light on the potential of **PESMP** as an α-amylase inhibitor.

## 2. Results and Discussion

### 2.1. Chemistry

**Scheme 1:**
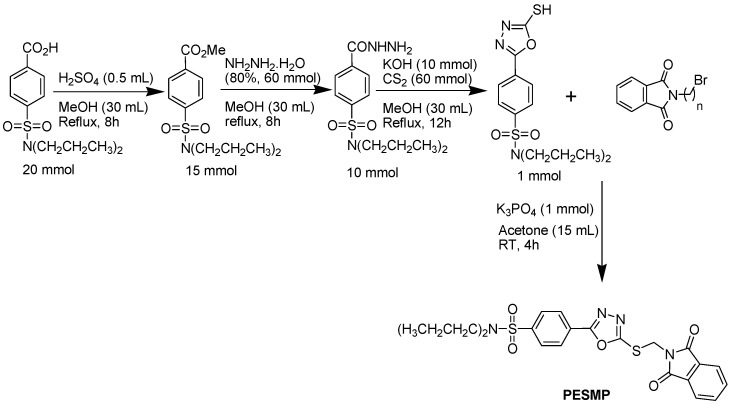
Synthetic path to access **PESMP** compound.

The **PESMP** compound was synthesized by a multi-step synthetic path, as depicted in Scheme 1. The purity of **PESMP** was confirmed based on thin-layer chromatography. The pure compound had a melting point of 154–155 °C. In the ^1^H NMR spectrum of **PESMP** (Figure S1), a triplet at *δ* 0.89 ppm (coupling constant = 7.35 Hz) was designated for six protons. A multiplet for four protons of two methylene groups (CH_3_CH_2_CH_2_) was observed at δ 1.49–1.66 ppm. Another multiplet for four protons of two methylene groups (N-CH_2_) attached to the N-atom was present at *δ* 3.00–3.22 ppm. A singlet for two protons at *δ* 5.41 ppm represented *S*CH_2_N. The aromatic protons were detected between δ 7.71 and 8.25 ppm.

Similarly, the ^13^C-NMR spectrum also established the synthesis of the desired **PESMP** (Figure S2). The methyl carbons were observed at δ 11.56, and the methylene carbons attached to the methyl group were found at δ 22.34 ppm. The methylene carbons attached to the nitrogen atom were found at δ 40.14 ppm, and a methylene carbon sandwiched between sulfur and nitrogen was present at *δ* 50.32 ppm. Aromatic carbons were found in the range of δ 124–143 ppm. The carbon atoms of 1,3,4-Oxadiazole ring were found at 165.86 and 166.81 ppm, whereas the carbonyl carbons of the phthalimide ring were found at 168.2 ppm. 

### 2.2. Single-Crystal Analysis

**Figure 2:**
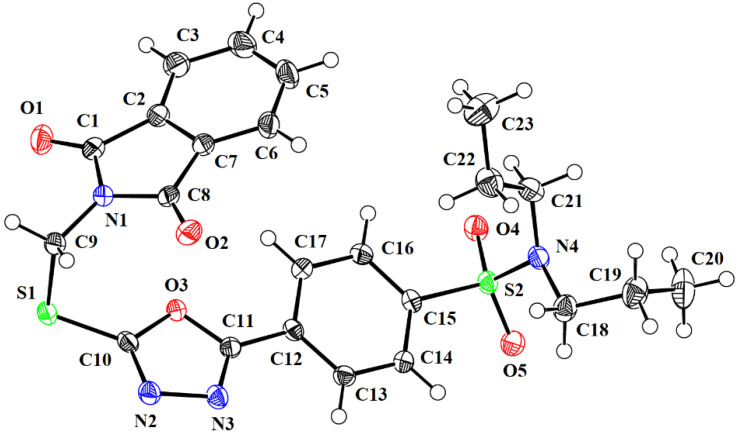
ORTEP presentation of **PESMP** which is visualized at a 50% level of probability. Hydrogen atoms are displayed by small circles with arbitrary radii.

In **PESMP** (Figure 2, Table 1), the 2-methylisoindoline-1,3-dione moiety A (C1-C9/N1/O1/O2), 2,5-dihydro-1,3,4-oxadiazole-2-thiol group B (C10/C11/N2/N3/O3/S1), and hydrosulfonylbenzene ring C (C12-C17/S2) are planar with root mean square (RMS) deviations of 0.0344, 0.0137, and 0.0069 Å, respectively. The dihedral angles A/B and B/C are 25.76(4) ° and 15.03(6) °, respectively. The root mean square plane of group B (C10/C11/N2/N3/O3/S1) is oriented at the dihedral angles of 25.76(4) ° and 15.03(6) ° with respect to moiety A (C1-C9/N1/O1/O2) and ring C (C12-C17/S2), respectively.

**Figure 3:**
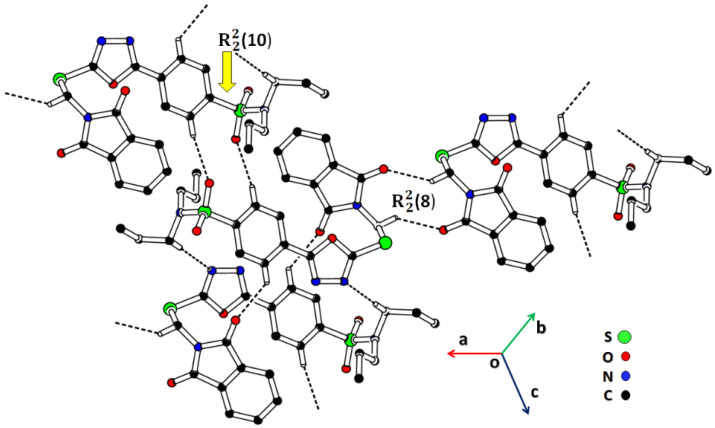
Packing diagram of **PESMP** elucidates the interlinkage of the molecules via C-H⋯O and C-H⋯N bondings. Only selected hydrogen atoms are shown to avoid ambiguity.

The geometry around the S2-atom is a distorted tetrahedron as the bond angles containing the S2-atom as a central atom range from 106.87(6) ° to 107.66(6) ° and the bond lengths range from 1.4337 (10) to 1.7732 (14) Å (Table S1). Two dipropylamine groups named group D (C18-C20/N4) and group E (N4-C21-C23) containing N4 as a common atom are directed at the dihedral angles of 39.8(8) ° and 42.8(9) °, respectively. The structure of **PESMP** is stabilized by intramolecular C-H⋯O bonding (Table 2 and Figure 3). 

Symmetry codes: (i) −*x*, −*y* + 2, −*z* + 1; (ii) −*x* + 1, −*y* + 1, −*z*; (iii) −*x* + 1, −*y* + 1, −*z* + 1; (iv) 1 + *x*, *y*, *z*; (v) 1 − *x*, 2 − *y*, 1 − *z*. Cg1, Cg2, and Cg3 are the centroids of the (C12/C17), (C10/C11/N2/N3/O3), and (C1/C2/C7/C8/N1) rings, respectively.

**Figure 4:**
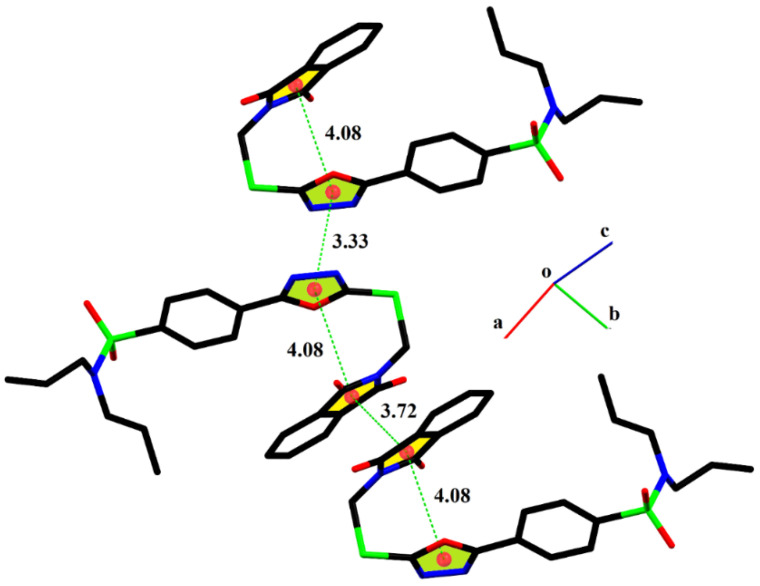
Graphical representation of intra- as well as inter-molecular offset π⋯π stacking interactions. For the purpose of clarity, hydrogen atoms are skipped. Distances are in Å.

The molecules are further connected via C-H···O bonding to form $R_{2}^{2}$(10) hydrogen-bonded loops (Figure 4). The $R_{2}^{2}$(10) H-bonded loops are connected through C-H⋯O and C-H⋯N bonding. As a result, $R_{2}^{2}$(8) H-bonded loops are formed. The weak intra-molecular π⋯π stacking interactions are unveiled via crystal packing. The five-membered ring of group A is involved in an intra-molecular π⋯π stacking interaction with the five-membered ring of group B with an inter-centroid separation value of 4.08 Å (Figure 4). The dihedral angle between the rings is 26.27(8) °.

**Figure 5:**
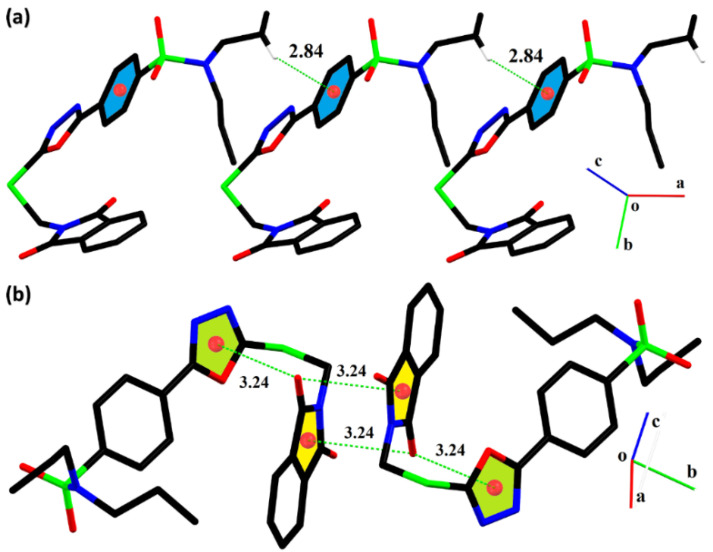
Representation for (**a**) C-H⋯π interactions that form an infinite chain of molecules besides the a-axis, (**b**) C-O⋯π interactions that interlink molecules through a dimeric form. Distances are in Å.

The inter-centroid separation of the intermolecular offset π⋯π stacking interactions is observed in the range of 3.33–3.72 Å, forming a chain of molecules along the [110] direction. The similar five-membered rings of the symmetry-related molecules are involved in inter-molecular offset π⋯π stacking interactions with the ring offset, or slippage ranges from 1.396 to 1.994 Å. The CH from one of the propyl groups participates in the C-H⋯π interaction with the aromatic ring of the symmetry-related molecules (1 + x, y, z), which forms an infinite chain of molecules that goes along the *a*-axis (Figure 5a, Table 2). An intra-molecular C-O⋯π interaction is found in **PESMP** with an O⋯π distance of 3.2430(12) ° and a C-O⋯π angle of 90.74(9) ° [26]. In crystal packing, the inter-molecular C-O⋯π interaction shows an O⋯π distance of 3.2417 (12) Å and a C-O⋯π angle of 80.93(8) °, which interlink the molecules in the form of dimers (Figure 5b, Table 2).

### 2.3. Hirshfeld Surface Analysis and Interaction Energy Calculation

**Figure 6:**
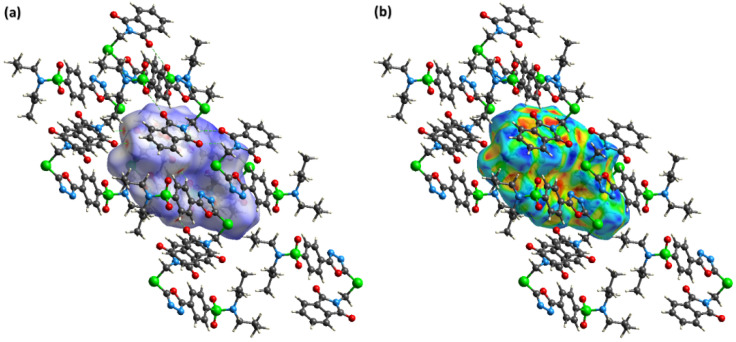
Hirshfeld surface of **PESMP** mapped over (**a**) *d*_norm_ using scope from −0.2450 to 1.5592 a.u., and (**b**) shape index using scope from −1 to 1 a.u.

A Hirshfeld surface (HS) analysis is an informative technique to elaborate and recognize non-covalent interactions in single crystals. It was developed to divide crystal densities into molecular fragments [27,28,29,30,31,32,33]. The hydrogen bonding interactions can be elucidated using HS plots over the normalized distance (*d*_norm_) property. The red- and blue-colored regions on surface stand for short and long contacts, respectively. Meanwhile, the spots on the white region indicate that the contacts have distances between their atoms equal to the sum of the van der Waals radii summation. Figure 6a,b show the HS surfaces over normalized distances (*d*_norm_) and shape index, respectively.

**Figure 7:**
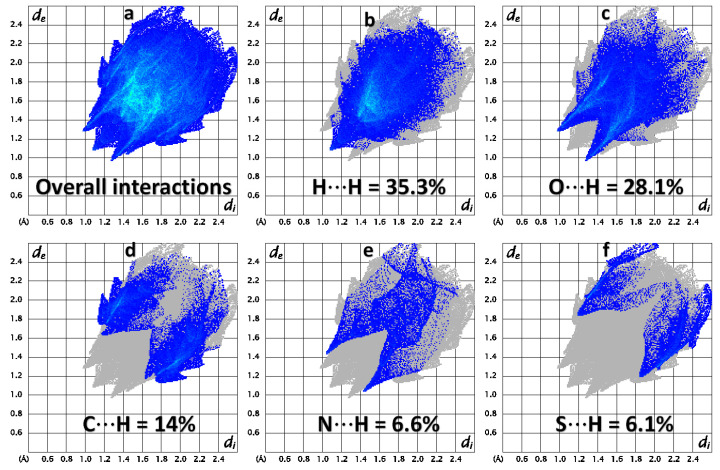
2D fingerprint plot of interactions and important interatomic contacts in **PESMP**.

The red spots around one of the *O*-atoms of the carbonyl and sulfonyl groups as well as the one *N*-atom of the 2,5-dihydro-1,3,4-oxadiazole confirm the contributions of the H-bonding interactions (Figure 6a). The interactions of π⋯π stacking can be recognized by extracting the HS over the shape index property. The occurrence of successive blue and red triangular patches on the HS around the aromatic ring shows π⋯π stacking interactions [34]. These areas are noted on the HS plots relevant to the shape index property for **PESMP** (Figure 6b), which confirms the existence of π⋯π stacking interactionsin **PESMP**. The contribution of the interatomic contacts in defining the overall packing of the crystal is determined by a 2D fingerprint plot analysis [25,35,36,37,38,39]. 2D fingerprints of all the interactions are generated (Figure 7). The reciprocal contacts are also included while forming the 2D plots.

Overall interaction is displayed in Figure 7a. The most significant contributor to crystal packing is announced to be the H⋯H contact, with 35.3% (Figure 7b). The other important contacts are O⋯H, C⋯H, N⋯H, and S⋯H, with contributions of 35.3%, 28.1%, 14%, 6.6%, and 6.1%, respectively (Figure 7c–f). The C⋯C and C⋯N contacts show lower contributions to the crystal packing as compared to the contacts, as shown in Figure 7, although these contacts are involved in π⋯π stacking interactions. The contacts that have comparatively smaller percentage contributions to the crystal packing are shown in Figure S3.

The enrichment ratio provides the actual contacts for a given single crystal [40,41]. The enrichment ratio is determined as a result of the division of the actual contact value obtained directly from CrystalExplorer by the ratio of the random contact determined theoretically. The enrichment ratio values for the pair of chemical species are summarized in Table 3. 

According to the compiled data in Table 3, C⋯C was the most favorable contact with an enrichment ratio of 3.31. The second most favorable contact was O⋯H, with an enrichment ratio of 1.39. The H⋯H and C⋯C contacts became less favorable by decreasing the enrichment ratio to less than one. A possible reason for the higher enrichment ratio of the C⋯N contact as compared to that of the C⋯C contact is that the intermolecular offset π⋯π stacking and C-O⋯π interaction interactions involve π-rings containing one or more nitrogen atoms. In crystal packing, various features of the single crystals, especially their mechanical properties, depending on the voids. For a single crystal to have good mechanical properties, the voids should be small. The voids were assessed by means of the Hartree–Fock theory, and the electronic densities of all the atoms were added up by supposing the spherical nature of the atoms [42,43,44,45,46]. Figure S4 is the graphical view of the voids in **PESMP**. The volume of voids was 148.35 Å^3^, which indicated that only 12.6% of the total volume was taken up by voids. The void research addressed the tight packing of molecules along with the lack of significant cavities in the crystal packing of the **PESMP** compound.

**Figure 8:**
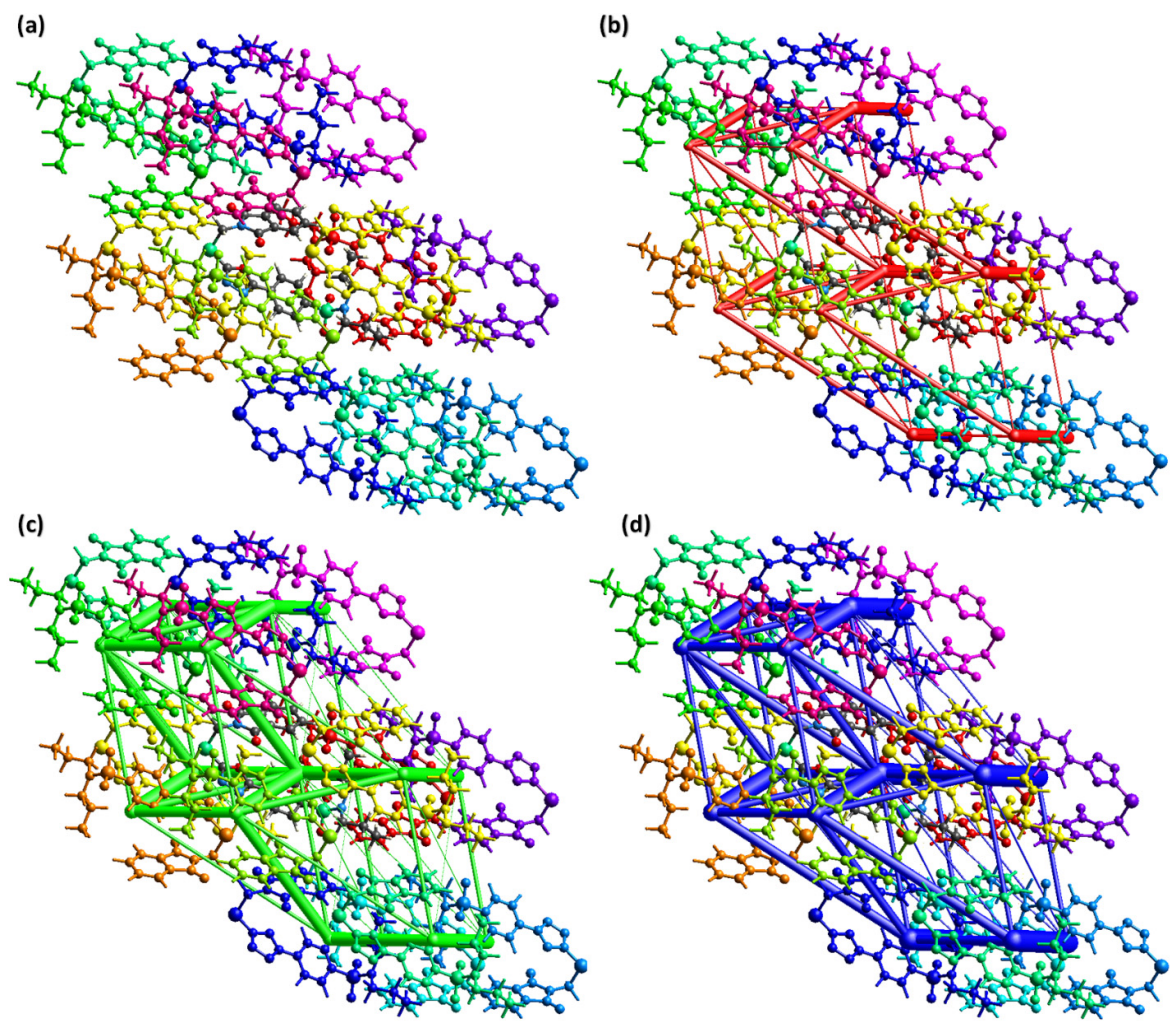
(**a**) Graphical representation of the molecules involved in the computations of the interaction energy between molecular pairs. Energy framework for (**b**) coulomb energy, (**c**) dispersion energy, and (**d**) total energy.

The CrystalExplorer software was employed for the calculation of the interaction energy within the molecular pairs. The calculation was carried out on a series of molecules located within 3.8 Å^3^ of the reference molecule using the B3LYP/6-31G(d,p) computational level. The symmetry-related molecules are displayed in Figure 8a. The obtained findings of the interaction energies among the molecular pairs are shown in Table S2. Table S3 collects the utilized atomic coordinates in the interaction energy calculations.

As summarized in Table S2, the highest attractive total energy was for the pair of molecules with an intermolecular distance of 8.14 Å. The highest energy value of −92.7 kJ/mol reflected attractive forces, indicating that the molecules were connected by inversion symmetry. The lowest attractive total energy with a value of −7.1 kJ/mol was found for the molecular pair with a distance of 19.10 Å between molecular centroids. For only one molecular pair, the total interaction energy was repulsive, with a value of 0.2 kJ/mol. Electrostatic energy was attractive for most of the molecular pairs except for the molecular pair R = 12.91 Å.

Figure 8b–d show the energy framework for coulomb, dispersion and total energy, respectively. The molecular centers were attached by the cylinders, and the thickness of the cylinder was directly related to the interaction strength. For the electrostatic coulomb energy, the thickness of the cylinders (Figure 8b) was smaller than that for the dispersion energy (Figure 8c), indicating that the dispersion energy was dominant over the coulomb energy.

### 2.4. DFT Calculations

#### 2.4.1. Frontier Molecular Orbitals Analysis 

Frontier molecular orbitals (FMOs) theory is employed as a dependable tool for estimating the electronic properties along with the chemical stability of a system [47,48]. In the context of FMOs, the electron density distribution in molecular orbitals is elucidated. FMOs also interpret the chemical affinity and relation of the examined molecule with other moieties. Furthermore, FMOs support our understanding of the reactive sites in any π-electron system [49]. FMOs investigate two principal orbitals. The first one is the highest occupied molecular orbital (HOMO), which holds and donates electrons as an electron donor. The second one is the lowest unoccupied molecular orbital (LUMO), which has a vacant orbital and tends to receive electrons [50]. Via *E*_HOMO_ and *E*_LUMO_ values, the FMO’s band gap (Δ*E*) is computed to elucidate the reactivity, electron transference properties, hardness, and softness of a molecule. The compounds possessing a greater energy difference between HOMO and LUMO are considered to be harder, kinetically more stable, and less reactive in their nature and vice versa [51]. 

**Figure 9:**
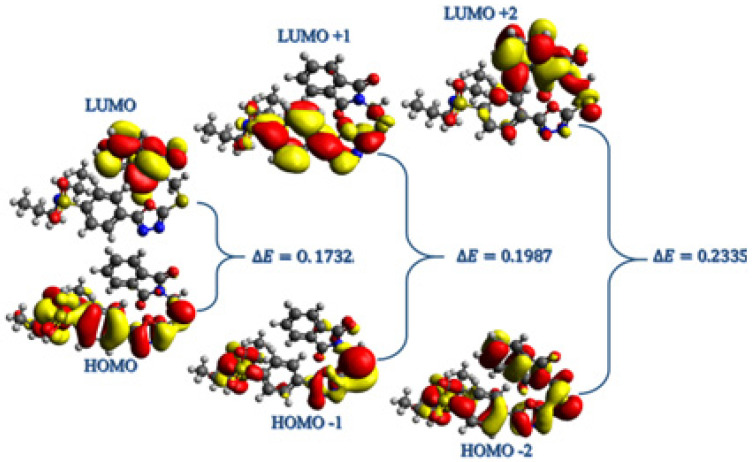
HOMO and LUMO diagrams of the **PESMP** compound, along with the band gap (Δ*E*, in a.u.) energetic values.

For the **PESMP** compound, FMO calculations were carried out, and the results of HOMO, HOMO − 1, HOMO − 2, LUMO, LUMO + 1, and LUMO + 2, along with their corresponding band gaps, are collected in Table 4. The molecular orbital sketch of the **PESMP** compound is shown in Figure 9.

As shown in Table 4, the energies of HOMO, HOMO − 1, and HOMO − 2 of the **PESMP** compound are −0.2678, −0.2808, and −0.2898 a.u. Furthermore, the energies of LUMO, LUMO + 1, and LUMO + 2 of the **PESMP** compound were −0.0946*,* −0.0822*,* and −0.0562 a.u., respectively. The energy gaps of HOMO and LUMO, HOMO − 1 and LUMO + 1, and HOMO − 2 and LUMO + 2 were 0.1732, 0.1987, and 0.2335 a.u., respectively.

In the **PESMP** compound, the charge density for HOMO was found over the oxadiazole and benzenesulfonamide moieties (Figure 9). The charge density for LUMO was situated on the phthalimide moiety, predicting an excellent charge transference from oxadiazole and benzenesulfonamide toward the phthalimide moiety.

#### 2.4.2. Global Reactivity Parameters (GRPs)

Global reactivity parameters (GRPs) are beneficial for calculating the stability and chemical reactivity of chemical systems [52]. For the **PESMP** compound, numerous GRPs, comprising ionization potential (*IP*), chemical potential (*μ*), electron affinity (*EA*), hardness (*η*), electronegativity (*X*), global softness (*σ*), and electrophilicity index (*ω*), were assessed by Koopman’s theorem as illustrated in the Section 3.2 [53].

The chemical hardness (*η*) of a molecule is related to the energy gap (Δ*E*) value. The overall softness and reactivity of the **PESMP** compound demonstrated an inverse relationship. *η* showed a value of 0.0866 a.u., revealing the high stability and lower reactivity of the considered compound. The ionization potential (*IP*) and electron affinity (*EA*) values were 0.2678 and 0.0946 a.u., respectively. Moreover, the values of *μ*, *ω*, and *σ* were 0.1812, 0.1896, and 5.7737 a.u., respectively. 

#### 2.4.3. Molecular Electrostatic Potential (MEP)

**Figure 10:**
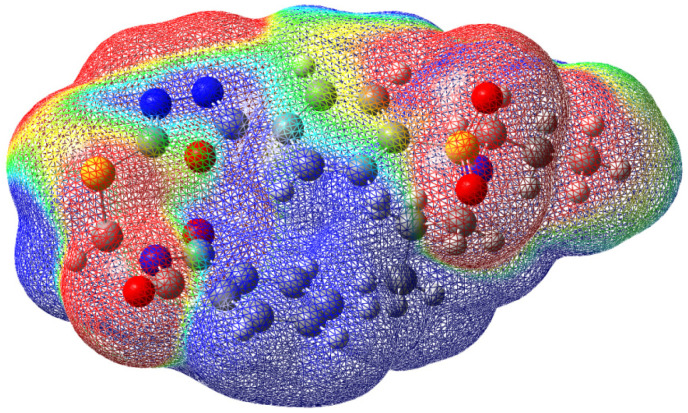
MEP map of the **PESMP** compound.

One way of determining the electrostatic potential values over the iso-electronic density map is to investigate a three-dimensional (3D) layout of the molecular electrostatic potential (MEP) surface. The MEP is conducted for the estimation of electrophilic and nucleophilic attacks on chemical systems [54]. The MEP diagram shows a variety of colors, red, blue, yellow, green, light blue, and blue, which represent significantly electron-rich, slightly electron-rich, neutral, slightly electron-deficient, and significantly electron-deficient regions, respectively [55]. The morphology, molecular size, and electrostatic potential amplitude of the **PESMP** compound are all clearly shown in Figure 10.

In the MEP map of the **PESMP** compound displayed in Figure 10, a red-colored zone is noted around the oxygen atoms. The likelihood of an electrophilic attack is therefore related to this area, which represents the electron-rich zone. On the hydrogen and certain carbon atoms, green and blue zones are seen, signifying electron-deficient zones, which point to a nucleophilic attack on these potential sites.

### 2.5. α-Amylase Inhibition Activity

The inhibitory potential of a probenecid-derived 1,3,4-oxadiazole–phthalimide hybrid **(PESMP)** against α-amylase was investigated at concentration levels ranging from 10 to 200 μg/mL, with **PESMP** inhibiting α-amylase by 81.06% at 200 μg/mL (Table 5). Acarbose, a commercially used standard α-amylase inhibitor, demonstrated inhibitory activity with an IC_50_ value of 8.80 ± 0.21 μg/mL.

### 2.6. Molecular Docking 

The efficiency of the AutoDock4.2.6 package in anticipating the correct inhibitor-α-amylase binding pose was validated. For validation purposes, the co-crystallized acarbose ligand was re-docked in the α-amylase enzyme’s active site, and the anticipated docking pose was compared to the experimental binding mode (PDB ID: 1ose [56]) (Figure S5). From Figure S5, the predicted docking pose was similar to the binding mode of the native structure of the acarbose inhibitor with an RMSD value of 0.23 Å, exhibiting several hydrogen bonds with the key amino acid residues of the α-amylase enzyme [25].

**Figure 11:**
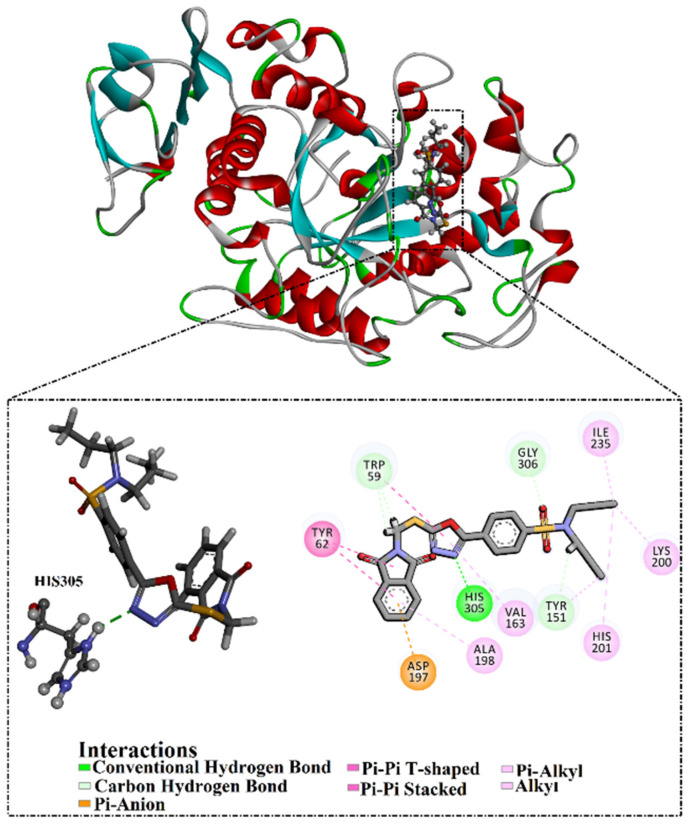
3D and 2D molecular interactions of **PESMP** with proximal residues of α-amylase enzyme.

The docking score and pose of **PESMP** against the α-amylase enzyme were predicted and compared to that of acarbose with the aid of AutoDock4.2.6. The anticipated docking score and pose are presented in Figure 11.

As depicted in Figure 11, **PESMP** demonstrated a −7.4 kcal/mol docking score. The potency of **PESMP** as an α-amylase inhibitor may be relevant to its ability to form a hydrogen bond with HIS305 (2.07 Å). Moreover, **PESMP** exhibited π⋯π T-shaped and π⋯π stacked interactions with the TYR62 residue and a π-anion interaction with the ASP197 residue. Moreover, **PESMP** demonstrated a carbon–hydrogen bond with the TRP59, TYR151, and GLY306 residues, a π-alkyl interaction with the VAL163 and ALA198 residues, and alkyl interactions with the LYS200, HIS201, and ILE235 residues (Figure 11). 

A pan-assay interference compound (PAINS) prediction for **PESMP** was also performed using the SmartsFilter web server (http://pasilla.health.unm.edu/tomcat/biocomp/smartsfilter, accessed on 1 December 2022). According to the predicted SmartsFilter results, **PESMP** passed the PAINS filter.

## 3. Experimental Section

### 3.1. Experimental Details

The current work was supported by the use of easily available laboratory-grade chemicals and reagents. The utilized solvents were of analytical grade and were employed without drying or distillation. The melting point was measured in an open capillary using a Gallenkamp melting point device (MP-D). Thin-layer chromatography was used to monitor all reactions using Merck pre-coated plates (silica gel 60 F254, 0.25 mm). Using UV rays (254 nm), fluorescence quenching was adopted to view the results. A Bruker AV-300 (300 MHz) spectrometer was employed to record the ^1^H and ^13^C NMR spectra. Single-crystal X-ray diffraction data were collected for **PESMP** compound on Bruker Kappa APEX-II CCD diffractometer with molybdenum X-ray source, which generated $k_{\alpha }$ radiations with a wavelength value of 0.71073 Å. The structure was solved and refined by SHELXT-2014 [57] and SHELXL-2019/2 [58], respectively. Mercury version 4.0 software [59] and PLATON [60] were employed for graphically illustrating single-crystal X-ray diffraction results.

#### 3.1.1. Synthetic Procedure for the Preparation of 4-(5-((1,3-Dioxoisoindolin-2-yl)methylthio)-1,3,4-oxadiazol-2-yl)-N,N-dipropylbenzene-sulfonamide (PESMP)

A modified multistep synthetic procedure was followed to access **PESMP**. 

Step I: In a typical experimental procedure, probenecid (PE) (30 mmol) was esterified in 20 mL of methanol using catalytic amounts of sulfuric acid (0.5 mL) at reflux temperature for 4 h. The mixture was extracted thrice with 30 mL of ethyl acetate after being neutralized with 50 mL of saturated sodium bicarbonate solution. The organic layer was dried over anhydrous sodium sulphate and filtered, and then the solvent was removed to obtain crude methyl ester of probenecid in a quantitative yield.

Step II: Methyl ester of probenecid (MPE) (with 25 mmol) was dissolved in 30 mL of methanol, and NH_2_NH_2_.H_2_O (80%, 0.06 mol) was added dropwise. After refluxing for 8 h, the reaction mixture was cooled to 25 °C and then dispensed into ice-cold water. Probenecid hydrazide (PEH) was precipitated, filtered, dried, and finally recrystallized from MeOH.

Step III: A solution of probenecid hydrazide (PEH) (20 mmol) in 10 mL of methanol and 3 equivalents of KOH dissolved methanol (30 mL) were added. After 10 min, carbon disulfide (30 mmol) was slowly added. Afterwards, the reaction mixture was refluxed for about 12 h. The reaction mixture was cooled down to 25 °C, concentrated, and poured into ice-cold water. The solution was acidified (pH = 2) with dilute hydrochloric acid. The precipitated probenecid oxadiazole (PEO) was washed with warm water and purified by recrystallization with the help of MeOH.

Step IV: Probenecid oxadiazole (PEO) (10 mmol) was dissolved in acetone and 12 mmol of potassium carbonate was added. After stirring for 10 min at 25 °C, 12 mmol of *N*-(bromomethyl)phthalimide was added, and the reaction mixture was stirred for another 6 h. The solvent was removed, and the crude was recrystallized from methanol to obtain pure **PESMP** (Scheme 1).

#### 3.1.2. Methyl 4-(dipropylsulfamoyl)benzoate (MPE)

Colorless solid; M.P.: 65–66 °C; yield: 87%; 1HNMR (300MHz, Chloroform-D) ppm: 8.16 (m, 2H), 7.88 (m, 2H), 3.97 (s, 3H), 3.11 (m, 4H), 1.54 (m, 4H), and 0.88 (t, J = 15 Hz, 6H); 13C NMR (75 MHz, Chloroform-D) ppm: 165.76, 144.27, 133.41, 130.22, 127.00, 52.61, 49.89, 21.91, and 11.15; FT-IR (cm^−1^): 3100, 3051 (C-H, Aromatic), 2935, 2873(C-H, Aliphatic), 1726 (Carbonyl), 1341(asym), and 1156(sym)(O=S=O).

#### 3.1.3. 4-(Dipropylsulfamoyl)benzoic acid hydrazide (PEH)

Colorless solid; M.P.: 116–118 °C; yield: 73%; 1HNMR (300MHz, DMSO-d6) ppm: 10.00 (s, 1H), 7.98 (d, J = 9 Hz, 2H), 7.86 (d, J = 9 Hz, 2H), 4.59 (s, 2H), 3.04 (t, J = 15 Hz, 4H), 1.46 (m, 4H), 0.80 (t, J = 15 Hz, 6H), and 13C NMR (75MHz, DMSO-d6); 164.94, 142.06, 137.31, 128.42, 127.29, 50.09, 22.09, and 11.42; FT-IR (cm^−1^): 3297, 3211 (NH_2_), 3086 (N-H), 3036, (C-H, Aromatic), 2964, 2873 (C-H, Aliphatic), 1658 (Carbonyl), 1328 (asym.), and 1155(sym.) O=S=O.

#### 3.1.4. N,N-dipropyl-4-(5-thioxo-4,5-dihydro-1,3,4-oxadiazol-2-yl)benzene sulfonamide (PEO)

Colorless solid; M.P.: 177–180 °C; yield: 77%; 1HNMR (300MHz, DMSO-d6) (ppm): 14.92 (s, 1H), 8.06 (d, J = 9 Hz, 2H), 7.97 (d, J = 9 Hz, 2H), 3.06 (t, J = 15 Hz, 4H), 1.46 (pnt, J = 15 Hz, 4H), and 0.80 (t, J = 15 Hz, 6H); 13C-NMR (75 MHz, DMSO-d6) ppm: 178.12, 159.79, 142.78, 128.24, 127.49, and 126.44; FT-IR (cm^−1^): 3068 (C-H, Aromatic), 2968 (C-H, Aliphatic), 1612, 1592 (C=N), 1340 (C-S), and 1158 (O=S=O).

#### 3.1.5. 2-((5-Phenyl-2-thioxo-1,3,4-oxadiazol-3(2H)-yl)methyl)isoindoline-1,3-dione (PESMP)

Colorless solid; M.P.: 154–155 °C; yield: 81%; 1H NMR (300 MHz, Chloroform-D) *δ* ppm: 0.89 (t, J = 7.35 Hz, 6 H, CH_3_), 1.49–1.66 (m, 4 H, CH_3_CH_2_), 3.00–3.22 (m, 4 H, NCH_2_), 5.41 (s, 2 H, *S*CH_2_N), 7.717.83 (m, 2 H, Aromatic), 7.85–7.91 (m, 2 H, Aromatic), 7.92–7.97 (m, 2 H, Aromatic), and 8.15–8.25 (m, 2 H, Aromatic); 13C NMR (75 MHz, Chloroform-D) *δ* ppm: 11.56 (Methyl-C), 22.34 (Methylene-C), 40.14 (*N*-mehtylene-C), 50.32 *S*-methylene-C), 77.42, 77.84, 124.32, 127.12, 127.85, 128.10, 132.06, 135.10, 143.64, 165.87 (Oxadiazole-C), 166.81 (Oxadiazole-C), and 168.2 (Carbonyl-C); FT-IR υ(cm^−1^): 3025 (Aromatic CH), 2960 (Methylene CH_2_), 1723 (Amide carbonyl), 1606, 1568 (Oxadiazole C=N), 1327, and 1154 (Sulfonamide SO_2_); anal. calcd for C_23_H_24_N_4_O_5_S_2_: C, 55.18; H, 4.83; N, 11.19; O, 15.98; and S, 12.81; found: C, 55.90; H, 4.88; N, 11.24; O, 16.01; and S, 12.84 (Figures S1 and S2).

### 3.2. Computational Details

#### 3.2.1. Hirshfeld Surface (HS) Analysis

Hirshfeld surface (HS) was analyzed for **PESMP** compound using CrystalExplorer version 21.5 [61] to further elucidate features of non-covalent interactions within a single crystal. In that spirit, the surface of normalized contact distance (*d*_norm_) was extracted using a color range from −0.2450 to 1.5592 a.u. Moreover, a map of the shape index property was generated with a color scope ranging from −1.0 a.u. (concave) to 1.0 au (convex). Two-dimensional fingerprints were created to unveil the contribution of interatomic contact to the overall crystal packing. To investigate the crystal packing environment widely, the enrichment ratio was evaluated by dividing the actual contact by the random contact theoretical proportion. Voids were also divulged using the Hartree–Fock theory to understand the properties of the single crystal from a mechanical point of view. Energy framework computations were performed to illustrate the energy framework and interaction energies of **PESMP** compound. Interaction energy calculations were carried out on a cluster of molecules located within 3.8 Å^3^ of the reference molecule using the B3LYP method with a 6-31G(d,p) basis set.

#### 3.2.2. Density Functional Theory (DFT) Analysis

DFT-based computations were performed with the Gaussian 09 program package [62] in conjugation with M06/6-311G(d,p) function for the quantum chemical investigations of synthesized PESMP compound. The input files were developed by using the GaussView 6.0 program [63]. To demonstrate the contributions of the electron density surrounding the molecular orbitals, FMOs theory was invoked. The distributions of HOMO and LUMO were generated. Using FMOs, energies of HOMO (*E*_HOMO_) and LUMO (*E*_LUMO_) were computed for the **PESMP** compound. Band gaps were accordingly assessed as the difference between the energies of LUMO and HOMO levels, as shown in the following Equation (1):

(1) $$\Delta E=\;E_{\mathrm{LUMO}}-E_{\mathrm{HOMO}}$$

Several global reactivity parameters (GRPs) were calculated for the **PESMP** compound as per Equations (2)–(8):

IP = −*E*_HOMO_ (2)

*EA = −E_LUMO_* (3)

(4) $$X=\frac{\left[IP-EA\right]}{2}$$

(5) $$\eta =\frac{\left[IP-EA\right]}{2}$$

(6) $$\mu =\frac{E_{\mathrm{HOMO}}+E_{\mathrm{LUMO}}}{2}$$

(7) $$\sigma =\frac{1}{2\eta }$$

(8) $$\omega =\frac{\mu^{2}}{2\eta }$$

MEP plot was generated for pictorially addressing the chemical nature of the **PESMP** compound. In MEP map, blue, green, yellow, orange, and red represented regions with decreasing order of positive electrostatic potential magnitudes. 

### 3.3. Enzyme Inhibition Activity

α-Amylase inhibition potential of probenecid-derived 1,3,4-oxadiazole–phthalimide hybrid (PESMP) was investigated employing the 3,5-dinitro-2-hydroxybenzoic acid (DNS) method [64]. **PESMP** (1 mg) and DNS (1 mg) were separately solvated in 1 mL of DMSO. A solution of **PESMP** and α-amylase (200 μL, pH 7.0) was maintained at 30 °C for 10 min. An aqueous starch solution (1%) was poured and the same temperature was regulated for another 3 min. The reaction was stopped by adding a DNS reagent (200 μL). The tube was immersed in boiling water. After 10 min, the tube was cooled down to 25 °C, and distilled water (5 mL) was added. The absorbance was obtained at 540 nm. Acarbose (10–250 μg/mL) was utilized as a standard inhibitor for the comparison of the inhibitory potential of **PESMP** in triplicate experiments, whereas 2% DMSO was used for the control incubation, which represents maximal enzyme activity. Percentage inhibition was calculated by using a formula (Equation (9)), and then IC_50_ values were calculated.

(9) $$\%\;\alpha \;amylase\;inhibition=\frac{\mathrm{Ao}-\mathrm{Ai}}{\mathrm{Ao}}\times 100$$

### 3.4. Molecular Docking

The employed docking calculations are detailedly described elsewhere [65,66,67]. Briefly, the α-amylase crystal structure (PDB access code: 1ose [56]) complexed with acarbose was retrieved and employed as a template for docking predictions. For the purpose of preparation, all ions, heteroatoms, and crystallographic water molecules were eliminated. The protonation states of the α-amylase enzyme were scrutinized with the assistance of the H++ website [68]. In addition, all missing hydrogens were added. The structure of **PESMP** was manually built, and then energetically minimized using MMFF94S force field within SZYBKI software [69,70]. The Gasteiger method was utilized to assign the atomic charges of **PESMP** and acarbose compound [71]. In this work, docking computation was conducted utilizing AutoDock4.2.6 software [72]. The genetic algorithm number (*GA*) was adjusted to 250. The maximum number of energy evaluations (*eval*) was set to 25,000,000. Other docking parameters were set to default values. The grid box was tailored to involve the binding pocket of the α-amylase enzyme, with a grid size of 60 Å × 60 Å × 60 Å. The coordinates of the grid center were *x* = 32.644, *y* = 38.464, and *z* = −3.166. The AutoGrid program was employed to extract the grid maps with a spacing of 0.375 Å. All molecular interactions were represented using BIOVIA Materials Studio [73]. 

## 4. Conclusions

An efficient, multistep synthetic approach was followed to synthesize good yields of 4-(5-((1,3-dioxoisoindolin-2-yl)methylthio)-1,3,4-oxadiazol-2-yl)-N,N-dipropylbenzene-sulfonamide (**PESMP**). Its synthesis was confirmed via structural validation using NMR (^1^H, ^13^C), FT-IR, and single-crystal X-ray diffraction analyses. The purity of **PESMP** was established on the basis of thin-layer chromatography. To obtain further insight into the features of the entitled compound, various DFT calculations were executed accordingly. The band gap value of the **PESMP** compound (0.1732 a.u.) for the HOMO-LUMO orbital was smaller than the band gap values of HOMO-LUMO ±1 and HOMO-LUMO ±2. The testing of **PESMP** against α-amylase revealed an IC_50_ = 10.60 ±0.16 μg/mL using acarbose (IC_50_= 8.80 ±0.21 μg/mL) as a standard drug. Molecular docking revealed the binding mode of the **PESMP** compound against the active site of α-amylase. **PESMP** and acarbose showed docking scores of −7.4 and −9.4 kcal/mol against the α-amylase enzyme, respectively. These findings shine new light on the potential of the **PESMP** compound as an α-amylase inhibitor.

## Figures and Tables

**Table 1:** Single-crystal X-ray diffraction data of **PESMP** compound.

Crystal Data	PESMP
CCDC	2184321
Chemical formula	C_23_H_24_N_4_O_5_S_2_
*M*_r_	500.58
Crystal system, space group	Triclinic $P\bar{1}$
Temperature (K)	293
a, b, c (Å)	7.9647 (1), 11.4141 (1), 13.5283 (2)
α, *β*, γ°	96.606 (1), 98.617 (1), 100.899 (1)
V (Å^3^)	1180.95 (3)
*Z*	2
Density (calculated)g/cm^−3^	1.408
F(000)	524
Radiation type	Cu Kα
Wavelength (λ)	1.54184
*µ* (mm^−1^)	2.411
Crystal size (mm)	0.08 × 0.06 × 0.05
Data Collection	
Diffractometer	Bruker Kappa APEXII CCD
Absorption correction	Multi-scan (SADABS; Bruker, 2007)
No. of measured, independent and observed [*I* > 2σ(*I*)] reflections	10075, 4146, 3939
*R*_int_	0.021
Theta range for data collection (°)	3.341 to 67.404
Index ranges	−4 ≤ h ≤ 8, −13 ≤ k ≤ 11, −16 ≤ l ≤ 16
(sin *θ*/λ)_max_ (Å^−1^)	0.599
Data Refinement	
*R*[F^2^ > 2σ(F^2^)], *wR*(F^2^), *S*	0.030, 0.079, 1.06
No. of reflections	4146
No. of parameters	309
H-atom treatment	H-atom parameters constrained
Δ*ρ*_max_, Δ*ρ*_min_ (e Å^−3^)	0.25, −0.39

**Table 2:** Geometry of hydrogen bond (bond length in Å and angle in °) for **PESMP**.

*D*—H···*A*	*D*—H	H···*A*	*D*···*A*	<(*D*—H···*A*)°
C9—H9A···O1i	0.97	2.44	3.1659 (18)	132
C16—H16···O4ii	0.93	2.53	3.4137 (17)	160
C18—H18A···O5	0.97	2.41	2.9028 (19)	111
C18—H18B···N2iii	0.97	2.62	3.5809 (19)	173
C21—H21A···O4	0.97	2.39	2.8934 (18)	112
C—H···π	C—H	H···π	C···π	<(C—H···π)°
C19—H19A···Cg1iv	0.97	2.84	3.6326 (18)	140
C—O···π	C—O	O···π	C···π	<(C—O···π)°
C8—O2···Cg2	1.2093 (17)	3.2430 (12)	3.4758 (15)	90.74 (9)
C8—O2···Cg3v	1.2093 (17)	3.2417 (12)	3.2765 (15)	80.93 (8)

**Table 3:** Enrichment ratio relevant to the pair of chemical species in **PESMP**. For contacts with less than 0.9% random contact, the enrichment ratio is not determined.

Contact %	Atom	H	C	N	O	S
	H	35.3	14	6.6	28.1	6.1
	C	14	0.2	4.7	1.7	0.1
	N	6.6	4.7	0.3	0.7	1
	O	28.1	1.7	0.7	0.4	0.9
	S	6.1	0.1	1	0.9	0
Surface%		62.7	10.45	6.8	16.1	4.05
Random Contacts %	Atom	H	C	N	O	S
	H	39.31				
	C	13.10	1.09			
	N	8.53	1.42	0.46		
	O	20.19	3.36	2.19	2.59	
	S	5.08	0.85	0.55	1.30	0.16
Enrichment ratio	Atom	H	C	N	O	S
	H	0.90				
	C	1.07	0.18			
	N	0.77	3.31			
	O	1.39	0.51	0.32	0.15	
	S	1.20			0.69	

**Table 4:** The *E*_HOMO_, *E*_LUMO_, and Δ*E* of **PESMP** are in a.u.

MOs	*E*	Δ*E*
LUMO	−0.0946	0.1732
HOMO	−0.2678	
LUMO + 1	−0.0822	0.1987
HOMO − 1	−0.2808	
LUMO + 2	−0.0562	0.2335
HOMO − 2	−0.2898	

**Table 5:** α-Amylase inhibition findings against **PESMP** compound.

Compound	Concentration (μg/mL)	% of Inhibition	IC_50_ Value (μg/mL)
PESMP	10 50 100 150 200	48.00 57.00 68.68 75.49 81.06	10.60 ± 0.16
Acarbose	10 100 200	55.21 73.83 82.55	8.80 ± 0.21

## Data Availability

Data is contained within the article and Supplementary Materials.

## Supplementary Materials

The following supporting information can be downloaded at: https://www.mdpi.com/article/10.3390/ph16030424/s1, Figure S1: ^1^H NMR spectrum of **PESMP**; Figure S2: ^13^C NMR spectrum of **PESMP**; Figure S3: 2D fingerprint plots of the remaining contacts; Figure S4: Graphical representation of voids, (a) 1st view, and (b) 2nd view along the a-axis; Figure S5: 3D superimposition of the resolved experimental structure (in gray) and the portended binding mode (in cyan) of acarbose complexed with α-amylase enzyme; Table S1: Selected bond lengths and bond angles in the **PESMP** compound; Table S2: Interaction energies (kJ/mol); Table S3: Cartesian coordinates of the selected atoms that are involved in interaction energy calculations.
