# Supplementary material for: Exploring Probenecid Derived 1,3,4-Oxadiazole-Phthalimide Hybrid as α-Amylase Inhibitor: Synthesis, Structural Investigation, and Molecular Modeling
Source: Pharmaceuticals (Basel). 2023 Mar 10;16(3):424. doi: 10.3390/ph16030424 (PMC10051969; doi:10.3390/ph16030424)
Supplement: Supplementary file 1 [file pharmaceuticals-16-00424-s001.zip › pharmaceuticals-2115937-supplementary.pdf]

^{1}H NMR (300 MHz, CHLOROFORM-*d*) δ ppm 0.89 (t, *J*=7.35 Hz, 6 H) 1.49 - 1.66 (m, 4 H) 3.00 - 3.22 (m, 4 H) 5.41 (s, 2 H) 7.71 - 7.83 (m, 2 H) 7.85 - 7.91 (m, 2 H) 7.92 - 7.97 (m, 2 H) 8.15 - 8.25 (m, 2 H)

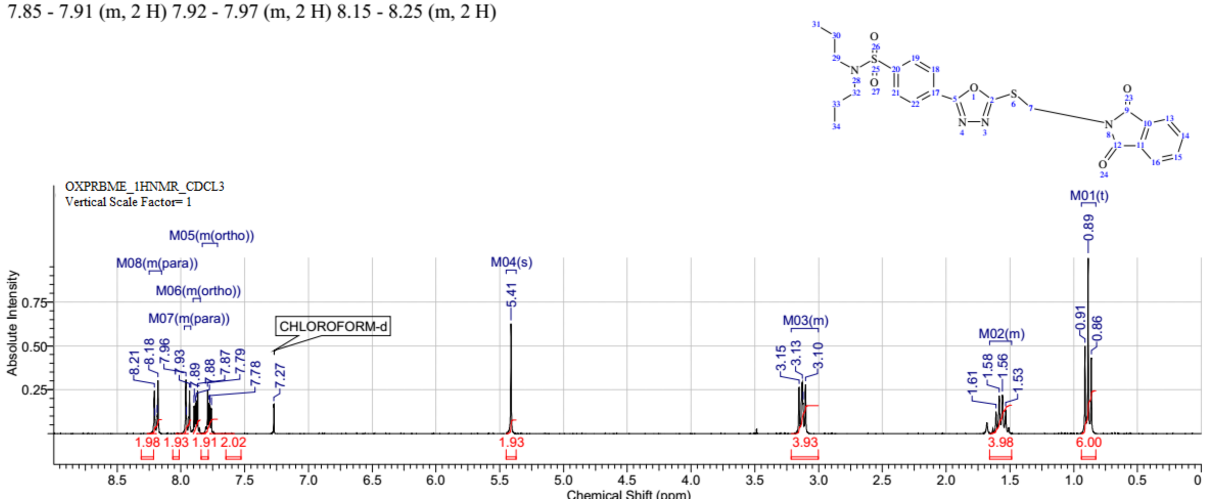

Figure S1: ^{1}H NMR spectrum of **PESMP**.

^{13}C NMR (75 MHz, CHLOROFORM-*d*) δ ppm 11.56 (s, 1 C) 22.34 (s, 1 C) 40.14 (s, 1 C) 50.32 (s, 1 C) 77.42 (s, 1 C) 77.84 (s, 1 C) 124.32 (s, 1 C) 127.12 (s, 1 C) 127.85 (s, 1 C) 128.10 (s, 1 C) 132.06 (s, 1 C) 135.10 (s, 1 C) 143.64 (s, 1 C) 166.81 (s, 1 C)

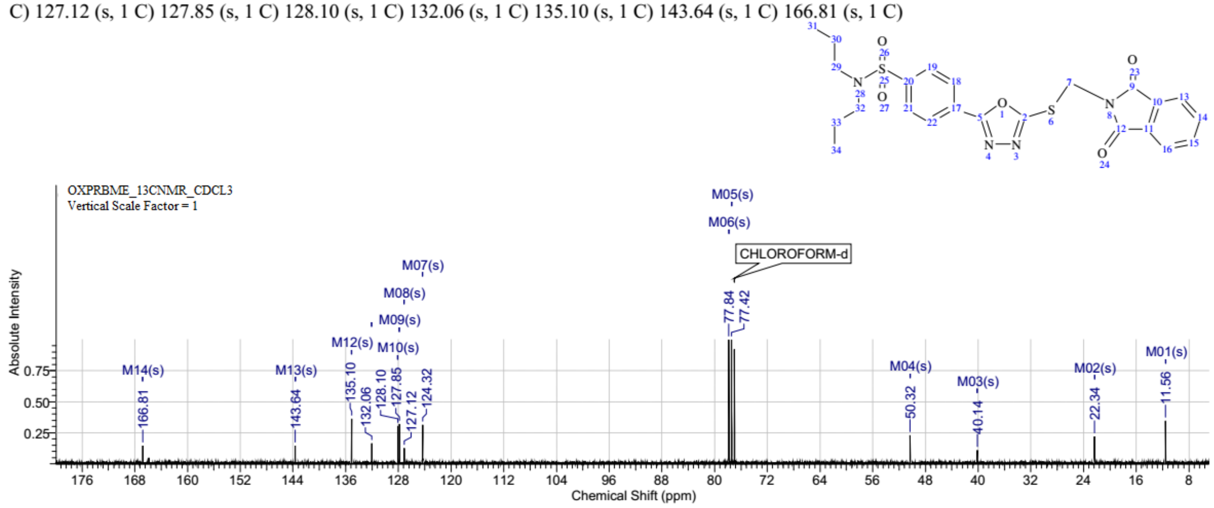

Figure S2: ^{13}C NMR spectrum of **PESMP**.

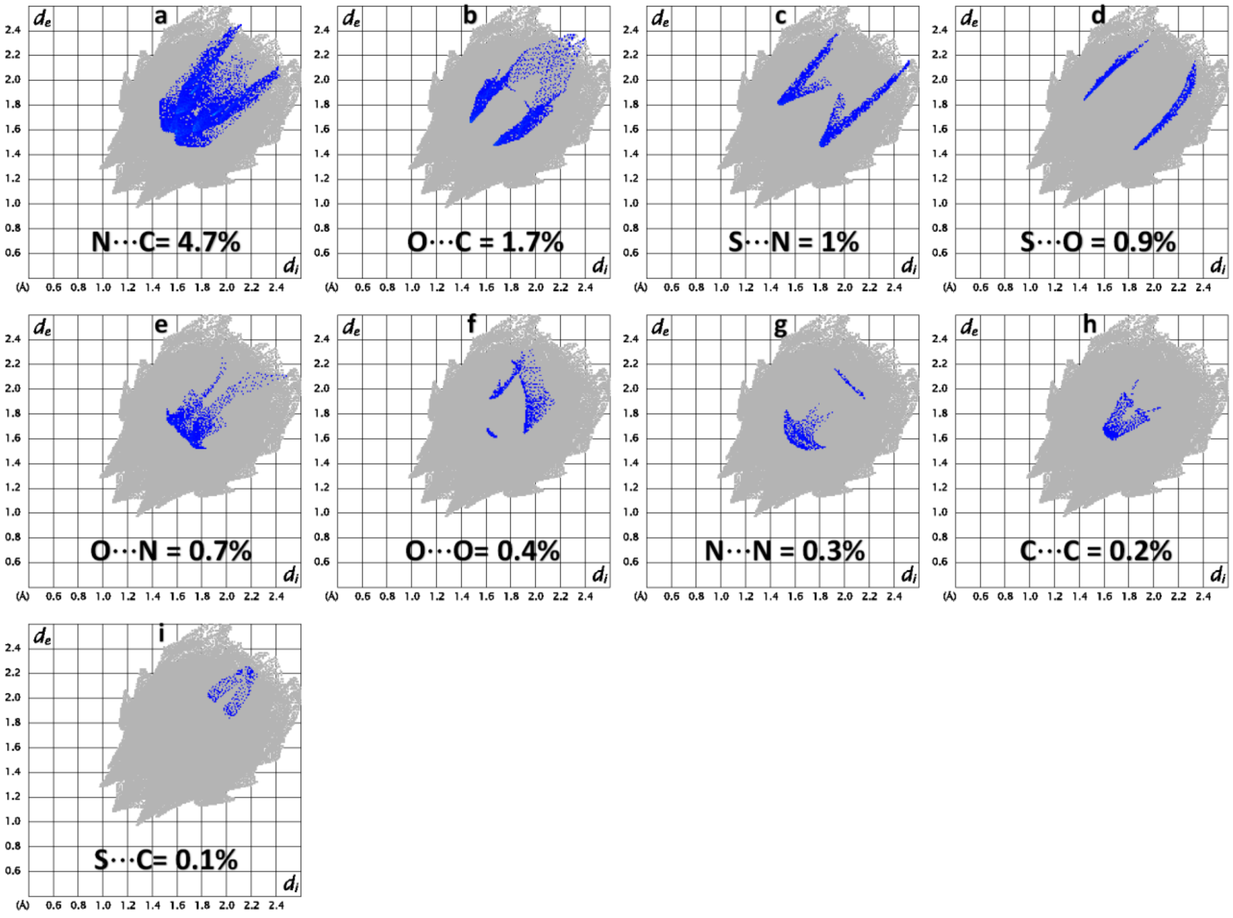

Figure S3: 2D fingerprint plots of the remaining contacts.

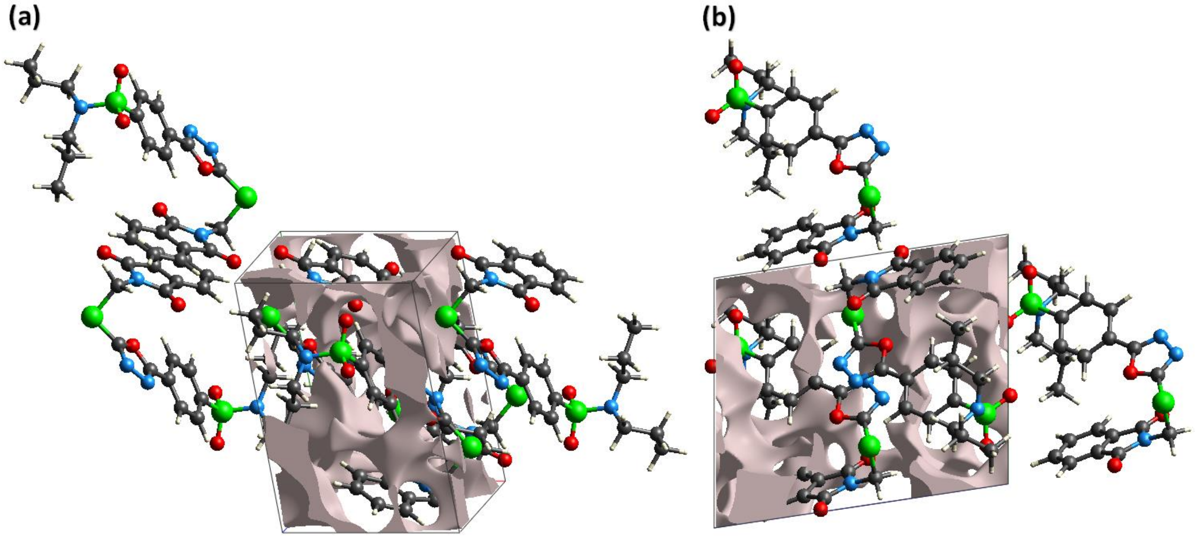

Figure S4: Graphical representation of voids, (**a**) 1st view, (**b**) 2nd view along the a-axis.

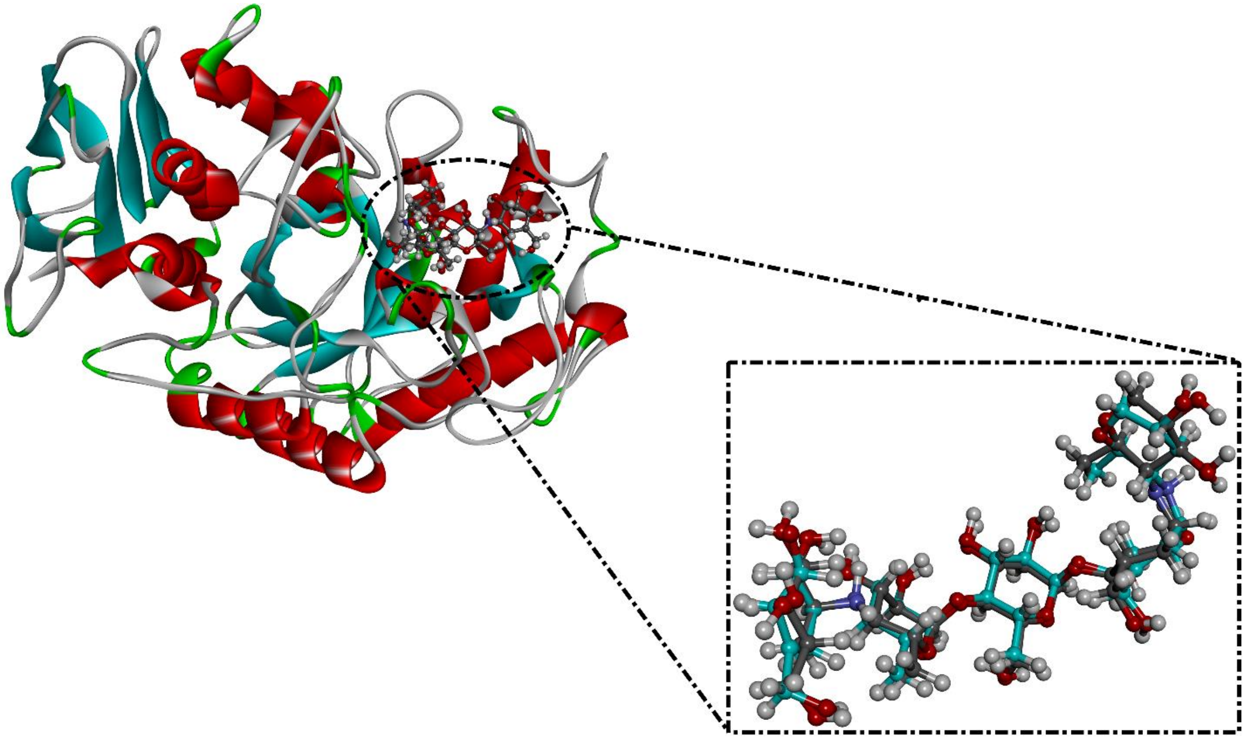

Figure S5. 3D superimposition of the resolved experimental structure (in gray) and the portended binding mode (in cyan) of acarbose complexed with α-amylase enzyme.

Table S1. Selected bond lengths and bond angles in the **PESMP** compound.

Chosen bond lengths (Å)		Chosen bond angles (⁰)	
**S1—C10**	**1.7407 (14)**	C10—S1—C9	98.07 (7)
S1—C9	1.8318 (14)	O4—S2—O5	119.32 (6)
S2—O4	1.4337 (10)	O4—S2—N4	107.66 (6)
S2—O5	1.4363 (10)	O5—S2—N4	107.58 (6)
S2—N4	1.6235 (12)	O4—S2—C15	107.26 (6)
S2—C15	1.7732 (14)	O5—S2—C15	106.87 (6)
O1—C1	1.2050 (18)	N4—S2—C15	107.65 (6)
O2—C8	1.2093 (17)	C11—O3—C10	102.21 (10)
O3—C11	1.3610 (16)	C8—N1—C1	111.86 (11)
O3—C10	1.3704 (16)	C8—N1—C9	123.34 (12)
N1—C8	1.4007 (19)	C1—N1—C9	124.36 (12)
N1—C1	1.4051 (18)	C10—N2—N3	106.39 (11)
N1—C9	1.4385 (18)	C11—N3—N2	106.03 (11)
N2—C10	1.2874 (18)	C21—N4—C18	117.38 (12)
N2—N3	1.4047 (16)	C21—N4—S2	118.09 (10)
N3—C11	1.2960 (18)	C18—N4—S2	117.26 (10)
N4—C21	1.4775 (19)	O1—C1—N1	124.49 (13)
N4—C18	1.4853 (18)	O1—C1—C2	129.96 (14)

Table S2: Interaction Energies (kJ/mol). *R* is the distance between molecular centroids (mean atomic position) in Å. Total energies, only reported for two benchmarked energy models, are the sum of the four energy components, scaled appropriately (see the scale factor table below)

	N	Symop	R	Electron Density	E_ele	E_pol	E_dis	E_rep	E_tot
	1	-x, -y, -z	8.14	B3LYP/6-31G(d,p)	-53.3	-18.5	-68.9	60.4	-92.7
	1	-x, -y, -z	9.69	B3LYP/6-31G(d,p)	-20.2	-3.1	-40.5	45.4	-30.9
	2	x, y, z	7.96	B3LYP/6-31G(d,p)	-5.7	-3.3	-53.5	26.2	-38.8
	1	-x, -y, -z	6.24	B3LYP/6-31G(d,p)	-43.2	-15.9	-74.0	68.9	-79.3
	1	-x, -y, -z	13.90	B3LYP/6-31G(d,p)	-24.6	-4.2	-17.6	0.0	-44.4
	2	x, y, z	15.10	B3LYP/6-31G(d,p)	-3.2	-0.7	-5.0	0.0	-8.3
	1	-x, -y, -z	15.04	B3LYP/6-31G(d,p)	-2.6	-1.6	-4.9	0.0	-8.2
	1	-x, -y, -z	19.10	B3LYP/6-31G(d,p)	-2.5	-0.1	-5.0	0.0	-7.1
	2	x, y, z	11.41	B3LYP/6-31G(d,p)	-7.5	-3.0	-21.0	10.8	-21.7
	1	-x, -y, -z	13.05	B3LYP/6-31G(d,p)	-0.8	-1.3	-17.5	0.0	-17.1
	1	-x, -y, -z	12.91	B3LYP/6-31G(d,p)	2.8	-0.6	-2.6	0.0	0.2
	1	-x, -y, -z	10.20	B3LYP/6-31G(d,p)	-7.7	-3.5	-57.6	40.6	-35.8

Scale factors for benchmarked energy models

See Mackenzie et al. IUCrJ (2017)

Energy Model	k_ele	k_pol	k_disp	k_rep
CE-HF ... HF/3-21G electron densities	1.019	0.651	0.901	0.811
CE-B3LYP ... B3LYP/6-31G(d,p) electron densities	1.057	0.740	0.871	0.618

Table: S3 Cartesian coordinates of the selected Atoms, (58 atoms), that are involved in interaction energy calcuations.
Label Symbol x y z Occ.

O4 O 2.8679 3.9589 -0.0881 1.000
H20A H 6.6633 2.1334 -0.0233 1.000
S1 S -2.6479 7.5548 6.9866 1.000
S2 S 3.1876 3.1363 1.0418 1.000
O1 O -2.1125 10.3789 4.7962 1.000
O2 O 1.2224 8.0972 6.9267 1.000
O3 O -0.8332 6.2202 5.5179 1.000
O5 O 2.9575 1.7204 0.9688 1.000
N1 N -0.6356 9.2183 6.1435 1.000
N2 N -1.0807 5.3913 7.5539 1.000
N3 N -0.1439 4.6047 6.8634 1.000
N4 N 4.7584 3.3607 1.3856 1.000
C1 C -1.0325 9.8720 4.9647 1.000
C2 C 0.1316 9.7761 4.0448 1.000
C3 C 0.2958 10.2617 2.7609 1.000
H3 H -0.4829 10.8301 2.2676 1.000
C4 C 1.5077 9.9882 2.1284 1.000
H4 H 1.6751 10.3508 1.1217 1.000
C5 C 2.5016 9.2624 2.7615 1.000
H5 H 3.4275 9.0625 2.2366 1.000
C6 C 2.3375 8.7825 4.0616 1.000
H6 H 3.1169 8.2198 4.5603 1.000
C7 C 1.1354 9.0586 4.6828 1.000
C8 C 0.6606 8.6975 6.0398 1.000
C9 C -1.5046 8.9595 7.2603 1.000
H9A H -2.0864 9.8498 7.4648 1.000
H9B H -0.8994 8.7535 8.1345 1.000
C10 C -1.4539 6.3131 6.7363 1.000
C11 C -0.0334 5.1317 5.6846 1.000
C12 C 0.7789 4.6788 4.5605 1.000
C13 C 1.3458 3.4060 4.6125 1.000
H13 H 1.2157 2.7899 5.4937 1.000
C14 C 2.0744 2.9315 3.5377 1.000
H14 H 2.5087 1.9398 3.5671 1.000
C15 C 2.2447 3.7409 2.4164 1.000
C16 C 1.7133 5.0238 2.3682 1.000
H16 H 1.8770 5.6514 1.5011 1.000
C17 C 0.9726 5.4910 3.4398 1.000
H17 H 0.5436 6.4849 3.4099 1.000
C18 C 5.3652 2.4691 2.4066 1.000
H18A H 4.8115 1.5391 2.4435 1.000
H18B H 5.2934 2.9391 3.3796 1.000
C19 C 6.8199 2.1726 2.1013 1.000
C20 C 7.0327 1.4948 0.7697 1.000
H20B H 6.4969 0.5537 0.7541 1.000
C21 C 5.3095 4.7260 1.2615 1.000
H21A H 4.7317 5.2582 0.5159 1.000
H21B H 6.3238 4.6492 0.8899 1.000
C22 C 5.3306 5.5598 2.5306 1.000
H22A H 5.9859 5.0976 3.2584 1.000

H22B H 4.3345 5.6030 2.9534 1.000
C23 C 5.8189 6.9719 2.2263 1.000
H23A H 5.1582 7.4342 1.5035 1.000
H23C H 5.8224 7.5568 3.1378 1.000
H19A H 7.3763 3.1016 2.1136 1.000
H19B H 7.2195 1.5393 2.8838 1.000
H20C H 8.0898 1.3111 0.6227 1.000
H23B H 6.8224 6.9275 1.8216 1.000

Unselected Atoms
870 atoms, Cartesian coordinates
Label Symbol x y z Occ.

H20C H -2.2833 9.8971 -0.6227 1.000
S1 S -1.5372 1.6782 6.2423 1.000
O3 O -3.3519 3.0129 7.7110 1.000
N2 N -3.1044 3.8417 5.6751 1.000
N3 N -4.0412 4.6284 6.3655 1.000
H5 H -4.5372 9.0625 2.2366 1.000
H6 H -4.8478 8.2198 4.5603 1.000
C10 C -2.7312 2.9200 6.4926 1.000
C11 C -4.1517 4.1013 7.5443 1.000
C13 C -5.5309 5.8270 8.6164 1.000
H13 H -5.4008 6.4431 7.7352 1.000
C19 C -1.1448 2.1726 2.1013 1.000
H19B H -3.4398 7.6938 10.3452 1.000
H21B H -1.6409 4.6492 0.8899 1.000
C22 C -2.6341 5.5598 2.5306 1.000
H22A H -1.9788 5.0976 3.2584 1.000
C23 C -2.1458 6.9719 2.2263 1.000
H23C H -2.1423 7.5568 3.1378 1.000
S1 S -3.6954 12.8865 6.2423 1.000
O1 O -4.2307 10.0623 8.4328 1.000
C1 C -5.3108 10.5692 8.2643 1.000
C9 C -4.8386 11.4817 5.9686 1.000
H9A H -4.2569 10.5915 5.7641 1.000
H9B H -5.4438 11.6878 5.0945 1.000
C20 C -3.0902 12.7030 0.7697 1.000
H20B H -3.6259 11.7620 0.7541 1.000
O5 O 5.0072 -1.7204 -0.9688 1.000
H20B H 1.4678 -0.5537 -0.7540 1.000
H20C H 7.8396 -1.3111 -0.6227 1.000
C2 C 2.2898 -1.4321 4.0448 1.000
C3 C 2.4540 -0.9465 2.7609 1.000
H3 H 1.6753 -0.3781 2.2676 1.000
C4 C 3.6659 -1.2200 2.1284 1.000
H4 H 3.8333 -0.8575 1.1217 1.000
C5 C 4.6597 -1.9458 2.7615 1.000
H5 H 5.5857 -2.1458 2.2366 1.000
S2 S 2.6189 8.0719 -1.0418 1.000
O5 O 2.8490 9.4878 -0.9688 1.000
N4 N 1.0481 7.8475 -1.3856 1.000

C3 C 5.5107 0.9465 -2.7609 1.000
H3 H 6.2894 0.3781 -2.2676 1.000
C4 C 4.2988 1.2200 -2.1284 1.000
H4 H 4.1314 0.8575 -1.1217 1.000
C5 C 3.3050 1.9458 -2.7615 1.000
H5 H 2.3790 2.1458 -2.2366 1.000
C16 C 4.0933 6.1844 -2.3682 1.000
H16 H 3.9295 5.5568 -1.5011 1.000
H18A H 0.9950 9.6691 -2.4435 1.000
C20 C -1.2261 9.7134 -0.7697 1.000
H20B H -0.6904 10.6545 -0.7540 1.000
H20C H 5.6814 9.8971 -0.6227 1.000
C21 C 0.4971 6.4822 -1.2615 1.000
H21A H 1.0749 5.9500 -0.5159 1.000
H21B H -0.5173 6.5590 -0.8899 1.000
H22B H 1.4721 5.6053 -2.9534 1.000
C23 C -0.0124 4.2363 -2.2263 1.000
H23A H 0.6483 3.7740 -1.5035 1.000
H23B H 6.9488 4.2807 -1.8216 1.000
S1 S 6.4275 1.6782 6.2423 1.000
O2 O 2.5573 1.1358 6.3023 1.000
O4 O 2.9387 7.2493 0.0881 1.000
N2 N 4.8603 3.8417 5.6751 1.000
N3 N 3.9235 4.6284 6.3655 1.000
H6 H 0.6627 1.0132 8.6687 1.000
C8 C 3.1190 0.5355 7.1891 1.000
C9 C 5.2842 0.2735 5.9686 1.000
H9B H 4.6791 0.4795 5.0945 1.000
C10 C 5.2335 2.9200 6.4926 1.000
C11 C 3.8130 4.1013 7.5443 1.000
C12 C 3.0007 4.5542 8.6684 1.000
C13 C 2.4338 5.8270 8.6164 1.000
H13 H 2.5639 6.4431 7.7352 1.000
C14 C 1.7053 6.3015 9.6913 1.000
H14 H 1.2709 7.2932 9.6619 1.000
C18 C -1.5856 6.7640 10.8223 1.000
H18A H -1.0319 7.6939 10.7854 1.000
H18B H -1.5138 6.2939 9.8493 1.000
H19A H -0.5884 3.1016 2.1136 1.000
H19B H -0.7452 1.5393 2.8838 1.000
H20A H -0.8568 9.0748 0.0233 1.000
H20C H 0.1251 1.3111 0.6227 1.000
C22 C -1.5509 3.6732 10.6984 1.000
H22A H -2.2063 4.1354 9.9705 1.000
H22B H -0.5549 3.6301 10.2756 1.000
H23B H -1.1423 6.9275 1.8216 1.000
H4 H 1.9733 12.0657 -1.1217 1.000
O1 O 3.7340 10.0623 8.4328 1.000
O2 O 0.3991 12.3440 6.3023 1.000
O5 O 0.7993 12.9286 0.9688 1.000
N1 N 2.2570 11.2229 7.0854 1.000
C1 C 2.6539 10.5692 8.2643 1.000
C2 C 1.4898 10.6651 9.1842 1.000

C3 C 1.3256 10.1795 10.4681 1.000
C4 C 0.1137 10.4531 11.1005 1.000
C5 C -0.8801 11.1789 10.4674 1.000
C6 C -0.7161 11.6588 9.1674 1.000
H6 H -1.4955 12.2215 8.6687 1.000
C7 C 0.4860 11.3827 8.5462 1.000
C8 C 0.9608 11.7438 7.1891 1.000
C9 C 3.1261 11.4817 5.9686 1.000
H9A H 3.7078 10.5915 5.7641 1.000
H9B H 2.5209 11.6878 5.0945 1.000
C14 C -0.0838 14.1398 3.5377 1.000
H14 H 0.3505 13.1480 3.5671 1.000
H18A H 2.6534 12.7473 2.4435 1.000
H19B H -2.9034 12.7475 2.8838 1.000
H20B H 4.3388 11.7620 0.7541 1.000
H20C H -2.0331 12.5193 0.6227 1.000
C20 C 8.8967 -1.4948 -0.7697 1.000
H20B H 9.4325 -0.5537 -0.7540 1.000
O1 O 8.0104 -0.8293 4.7962 1.000
H3 H 9.6400 -0.3781 2.2676 1.000
H20A H 9.2661 -2.1334 0.0233 1.000
H5 H 10.3437 2.1458 -2.2366 1.000
C20 C 6.7386 9.7134 -0.7697 1.000
C21 C 8.4618 6.4822 -1.2615 1.000
H21A H 9.0396 5.9500 -0.5159 1.000
H21B H 7.4474 6.5590 -0.8899 1.000
C23 C 7.9523 4.2363 -2.2263 1.000
H23A H 8.6130 3.7740 -1.5035 1.000
H23C H 7.9489 3.6514 -3.1378 1.000
S1 S 5.3168 7.5548 6.9866 1.000
S2 S 11.1523 3.1363 1.0418 1.000
O1 O 5.8522 10.3789 4.7962 1.000
O3 O 7.1315 6.2202 5.5179 1.000
O5 O 10.9222 1.7204 0.9688 1.000
N1 N 7.3291 9.2183 6.1435 1.000
C1 C 6.9322 9.8720 4.9647 1.000
C2 C 8.0963 9.7761 4.0448 1.000
C3 C 8.2605 10.2617 2.7609 1.000
H3 H 7.4818 10.8301 2.2676 1.000
C10 C 6.5108 6.3131 6.7363 1.000
C11 C 7.9313 5.1317 5.6846 1.000
C12 C 8.7436 4.6788 4.5605 1.000
C13 C 9.3105 3.4060 4.6125 1.000
H13 H 9.1804 2.7899 5.4937 1.000
C14 C 10.0391 2.9315 3.5377 1.000
H14 H 10.4734 1.9398 3.5671 1.000
C15 C 10.2094 3.7409 2.4164 1.000
C16 C 9.6780 5.0238 2.3682 1.000
H16 H 9.8417 5.6514 1.5011 1.000
C17 C 8.9373 5.4910 3.4398 1.000
H17 H 8.5083 6.4849 3.4099 1.000
H20A H 7.1079 9.0748 0.0233 1.000
C9 C -2.6805 0.2735 5.9686 1.000

C5 C -5.4631 9.2624 2.7615 1.000
C6 C -5.6272 8.7825 4.0616 1.000
C12 C -4.9640 4.5542 8.6684 1.000
C14 C -6.2594 6.3015 9.6913 1.000
C18 C -2.5995 2.4691 2.4066 1.000
C20 C -0.9320 1.4948 0.7697 1.000
C19 C -3.0403 7.0604 11.1277 1.000
C21 C -2.6552 4.7260 1.2615 1.000
H22B H -3.6302 5.6030 2.9534 1.000
H23A H -2.8065 7.4342 1.5035 1.000
C10 C -4.8893 14.1282 6.4926 1.000
N1 N -5.7077 11.2229 7.0854 1.000
C2 C -6.4749 10.6651 9.1842 1.000
C19 C -3.3029 13.3808 2.1013 1.000
H20A H -3.4595 13.3416 -0.0233 1.000
S2 S 4.7771 -3.1363 -1.0418 1.000
C20 C 0.9320 -1.4948 -0.7697 1.000
C1 C 1.1257 -1.3362 4.9647 1.000
C7 C 3.2936 -2.1496 4.6828 1.000
C6 C 4.4957 -2.4257 4.0616 1.000
C15 C 3.5619 7.4674 -2.4164 1.000
C18 C 0.4413 8.7391 -2.4066 1.000
C2 C 5.6749 1.4321 -4.0448 1.000
C6 C 3.4690 2.4257 -4.0616 1.000
C17 C 4.8339 5.7172 -3.4398 1.000
C19 C -1.0134 9.0356 -2.1013 1.000
C22 C 0.4760 5.6484 -2.5306 1.000
H23B H -1.0159 4.2807 -1.8216 1.000
H23C H -0.0158 3.6514 -3.1378 1.000
C6 C 1.4421 0.4506 9.1674 1.000
N1 N 4.4152 0.0147 7.0854 1.000
C7 C 2.6442 0.1744 8.5462 1.000
H9A H 5.8660 -0.6167 5.7641 1.000
O3 O 4.6128 3.0129 7.7110 1.000
C17 C 2.8070 3.7420 9.7892 1.000
C15 C 1.5350 5.4922 10.8125 1.000
N4 N -0.9788 5.8723 11.8433 1.000
C21 C -1.5298 4.5070 11.9674 1.000
C23 C -2.0393 2.2611 11.0026 1.000
C4 C 2.1406 12.4283 -2.1284 1.000
S2 S 1.0294 14.3445 1.0418 1.000
H3 H 2.1044 9.6112 10.9614 1.000
H4 H -0.0537 10.0905 12.1073 1.000
H5 H -1.8061 11.3788 10.9923 1.000
S1 S 4.2693 12.8865 6.2423 1.000
C13 C -0.8123 14.6142 4.6125 1.000
C15 C 0.0865 14.9491 2.4164 1.000
C18 C 3.2071 13.6773 2.4066 1.000
C20 C 4.8745 12.7030 0.7697 1.000
C19 C 9.1095 -2.1726 -2.1013 1.000
C1 C 9.0904 -1.3362 4.9647 1.000
C3 C 10.4187 -0.9465 2.7609 1.000
C5 C 11.2697 1.9458 -2.7615 1.000

C19 C 6.9513 9.0356 -2.1013 1.000
H20B H 7.2743 10.6545 -0.7540 1.000
N4 N 9.0128 7.8475 -1.3856 1.000
C22 C 8.4407 5.6484 -2.5306 1.000
C9 C 6.4601 8.9595 7.2603 1.000
O4 O 10.8326 3.9589 -0.0881 1.000
N4 N 12.7231 3.3607 1.3856 1.000
C8 C 8.6253 8.6975 6.0398 1.000
C7 C 9.1001 9.0586 4.6828 1.000
C4 C 9.4724 9.9882 2.1284 1.000
N2 N 6.8840 5.3913 7.5539 1.000
N3 N 7.8208 4.6047 6.8634 1.000
N1 N -3.5495 0.0147 7.0854 1.000
H9A H -2.0987 -0.6167 5.7641 1.000
H9B H -3.2856 0.4795 5.0945 1.000
C4 C -6.4570 9.9882 2.1284 1.000
C7 C -6.8293 9.0586 4.6828 1.000
C17 C -5.1577 3.7420 9.7892 1.000
H14 H -6.6938 7.2932 9.6619 1.000
C15 C -6.4297 5.4922 10.8125 1.000
N4 N -3.2063 3.3607 1.3856 1.000
H18A H -3.1532 1.5391 2.4435 1.000
H18B H -2.6713 2.9391 3.3796 1.000
H20A H -1.3014 2.1334 -0.0233 1.000
H20B H -1.4678 0.5537 0.7541 1.000
H19A H -3.5967 6.1314 11.1154 1.000
C20 C -3.2530 7.7382 12.4593 1.000
H21A H -3.2330 5.2582 0.5159 1.000
O3 O -5.5100 14.2211 7.7110 1.000
N2 N -5.2625 15.0499 5.6751 1.000
C8 C -7.0039 11.7438 7.1891 1.000
C3 C -6.6391 10.1795 10.4681 1.000
C7 C -7.4787 11.3827 8.5462 1.000
C18 C -4.7576 13.6773 2.4066 1.000
H19A H -2.7465 14.3098 2.1136 1.000
O4 O 5.0968 -3.9589 0.0881 1.000
N4 N 3.2063 -3.3607 -1.3856 1.000
C15 C 5.7200 -3.7409 -2.4164 1.000
C19 C 1.1448 -2.1726 -2.1013 1.000
H20A H 1.3014 -2.1334 0.0233 1.000
H20C H -0.1251 -1.3111 -0.6227 1.000
O1 O 0.0457 -0.8293 4.7962 1.000
N1 N 1.5226 -1.9899 6.1435 1.000
C8 C 2.8188 -2.5107 6.0398 1.000
H6 H 5.2751 -2.9884 4.5603 1.000
C14 C 3.7322 8.2767 -3.5377 1.000
H18B H 0.5131 8.2691 -3.3796 1.000
C1 C 6.8390 1.3362 -4.9647 1.000
C7 C 4.6711 2.1496 -4.6828 1.000
H6 H 2.6896 2.9884 -4.5603 1.000
C12 C 5.0277 6.5294 -4.5605 1.000
H17 H 5.2630 4.7233 -3.4099 1.000
H19A H -1.5698 8.1066 -2.1136 1.000

H19B H -1.4129 9.6689 -2.8838 1.000
H22A H -0.1794 6.1106 -3.2584 1.000
C5 C 1.2780 -0.0294 10.4674 1.000
C1 C 4.8121 -0.6390 8.2643 1.000
C2 C 3.6480 -0.5431 9.1842 1.000
C16 C 2.0663 4.2093 10.8607 1.000
H17 H 3.2360 2.7481 9.8191 1.000
S2 S 0.5920 6.0967 12.1872 1.000
H21A H -0.9521 3.9748 12.7130 1.000
H21B H -2.5442 4.5838 12.3390 1.000
H23A H -1.3786 1.7988 11.7255 1.000
H23B H -3.0428 2.3055 11.4073 1.000
H23C H -2.0427 1.6762 10.0912 1.000
C3 C 3.3526 12.1547 -2.7609 1.000
C5 C 1.1468 13.1540 -2.7615 1.000
O4 O 0.7097 15.1671 -0.0881 1.000
N4 N 2.6002 14.5689 1.3856 1.000
C10 C 3.0754 14.1282 6.4926 1.000
C12 C -1.3793 15.8871 4.5605 1.000
H13 H -0.9424 13.9981 5.4937 1.000
C16 C -0.4449 16.2320 2.3682 1.000
H18B H 3.1353 14.1473 3.3796 1.000
C19 C 4.6618 13.3808 2.1013 1.000
H20A H 4.5052 13.3416 -0.0233 1.000
H20C H 5.9316 12.5193 0.6227 1.000
C18 C 10.5642 -2.4691 -2.4066 1.000
H19A H 8.5531 -3.1016 -2.1136 1.000
H19B H 8.7099 -1.5393 -2.8838 1.000
N1 N 9.4873 -1.9899 6.1435 1.000
C2 C 10.2545 -1.4321 4.0448 1.000
C4 C 11.6306 -1.2200 2.1284 1.000
C4 C 12.2635 1.2200 -2.1284 1.000
C6 C 11.4337 2.4257 -4.0616 1.000
C18 C 8.4060 8.7391 -2.4066 1.000
H19A H 6.3949 8.1066 -2.1136 1.000
H19B H 6.5518 9.6689 -2.8838 1.000
S2 S 10.5836 8.0719 -1.0418 1.000
H22A H 7.7853 6.1106 -3.2584 1.000
H22B H 9.4368 5.6053 -2.9534 1.000
H9A H 5.8783 9.8498 7.4648 1.000
H9B H 7.0653 8.7535 8.1345 1.000
C18 C 13.3299 2.4691 2.4066 1.000
C21 C 13.2742 4.7260 1.2615 1.000
O2 O 9.1871 8.0972 6.9267 1.000
C6 C 10.3022 8.7825 4.0616 1.000
H4 H 9.6398 10.3508 1.1217 1.000
C5 C 10.4663 9.2624 2.7615 1.000
C1 C -3.1526 -0.6390 8.2643 1.000
C8 C -4.8457 0.5355 7.1891 1.000
C3 C -7.6689 10.2617 2.7609 1.000
H4 H -6.2896 10.3508 1.1217 1.000
C2 C -7.8331 9.7761 4.0448 1.000
C8 C -7.3041 8.6975 6.0398 1.000

C16 C -5.8984 4.2093 10.8607 1.000
H17 H -4.7287 2.7481 9.8191 1.000
S2 S -7.3727 6.0967 12.1872 1.000
S2 S -4.7771 3.1363 1.0418 1.000
H20A H -2.8837 7.0997 13.2522 1.000
H20B H -2.7173 8.6793 12.4749 1.000
H20C H -4.3102 7.9219 12.6063 1.000
C11 C -6.3099 15.3095 7.5443 1.000
N3 N -6.1993 15.8366 6.3655 1.000
O2 O -7.5656 12.3440 6.3023 1.000
H3 H -5.8603 9.6112 10.9614 1.000
C4 C -7.8510 10.4531 11.1005 1.000
C6 C -8.6808 11.6588 9.1674 1.000
N4 N -5.3645 14.5689 1.3856 1.000
H18A H -5.3113 12.7473 2.4435 1.000
H18B H -4.8294 14.1473 3.3796 1.000
C18 C 2.5995 -2.4691 -2.4066 1.000
C21 C 2.6552 -4.7260 -1.2615 1.000
C14 C 5.8903 -2.9315 -3.5377 1.000
C16 C 6.2514 -5.0238 -2.3682 1.000
H19A H 0.5884 -3.1016 -2.1136 1.000
H19B H 0.7452 -1.5393 -2.8838 1.000
C9 C 0.6536 -2.2487 7.2603 1.000
O2 O 3.3805 -3.1110 6.9267 1.000
C13 C 4.4607 7.8022 -4.6125 1.000
H14 H 3.2978 9.2684 -3.5671 1.000
O1 O 7.9190 0.8293 -4.7962 1.000
N1 N 6.4421 1.9899 -6.1435 1.000
C8 C 5.1459 2.5107 -6.0398 1.000
C11 C 5.8399 6.0765 -5.6846 1.000
C4 C 2.2719 -0.7551 11.1005 1.000
H5 H 0.3521 0.1706 10.9923 1.000
O1 O 5.8921 -1.1459 8.4328 1.000
C3 C 3.4838 -1.0287 10.4681 1.000
H16 H 1.9026 3.5816 11.7279 1.000
O4 O 0.9117 5.2741 13.3171 1.000
O5 O 0.8221 7.5126 12.2602 1.000
C2 C 3.5168 12.6403 -4.0448 1.000
H3 H 4.1313 11.5863 -2.2676 1.000
H5 H 0.2209 13.3540 -2.2366 1.000
C6 C 1.3109 13.6340 -4.0616 1.000
C21 C 3.1513 15.9342 1.2615 1.000
O3 O 2.4547 14.2211 7.7110 1.000
N2 N 2.7022 15.0499 5.6751 1.000
C11 C -2.1915 16.3399 5.6846 1.000
C17 C -1.1856 16.6992 3.4398 1.000
H16 H -0.2811 16.8597 1.5011 1.000
H19A H 5.2182 14.3098 2.1136 1.000
H19B H 5.0613 12.7475 2.8838 1.000
N4 N 11.1710 -3.3607 -1.3856 1.000
H18A H 11.1179 -1.5391 -2.4435 1.000
H18B H 10.6360 -2.9391 -3.3796 1.000
C8 C 10.7835 -2.5107 6.0398 1.000

C9 C 8.6183 -2.2487 7.2603 1.000
C7 C 11.2583 -2.1496 4.6828 1.000
H4 H 11.7980 -0.8575 1.1217 1.000
C5 C 12.6244 -1.9458 2.7615 1.000
C3 C 13.4754 0.9465 -2.7609 1.000
H4 H 12.0961 0.8575 -1.1217 1.000
H6 H 10.6543 2.9884 -4.5603 1.000
C7 C 12.6358 2.1496 -4.6828 1.000
H18A H 8.9597 9.6691 -2.4435 1.000
H18B H 8.4778 8.2691 -3.3796 1.000
O4 O 10.9034 7.2493 0.0881 1.000
O5 O 10.8137 9.4878 -0.9688 1.000
C15 C 11.5266 7.4674 -2.4164 1.000
H18A H 12.7762 1.5391 2.4435 1.000
H18B H 13.2581 2.9391 3.3796 1.000
C19 C 14.7846 2.1726 2.1013 1.000
H21A H 12.6964 5.2582 0.5159 1.000
H21B H 14.2885 4.6492 0.8899 1.000
C22 C 13.2953 5.5598 2.5306 1.000
H6 H 11.0816 8.2198 4.5603 1.000
H5 H 11.3922 9.0625 2.2366 1.000
O1 O -2.0726 -1.1459 8.4328 1.000
C2 C -4.3167 -0.5431 9.1842 1.000
O2 O -5.4074 1.1358 6.3023 1.000
C7 C -5.3205 0.1744 8.5462 1.000
H3 H -8.4476 10.8301 2.2676 1.000
C1 C -8.9972 9.8720 4.9647 1.000
O2 O -6.7423 8.0972 6.9267 1.000
N1 N -8.6003 9.2183 6.1435 1.000
H16 H -6.0621 3.5816 11.7279 1.000
O4 O -7.0530 5.2741 13.3171 1.000
O5 O -7.1426 7.5126 12.2602 1.000
N4 N -8.9435 5.8723 11.8433 1.000
O4 O -5.0968 3.9589 -0.0881 1.000
O5 O -5.0072 1.7204 0.9688 1.000
C15 C -5.7200 3.7409 2.4164 1.000
C12 C -7.1221 15.7624 8.6684 1.000
H4 H -8.0184 10.0905 12.1073 1.000
C5 C -8.8448 11.1789 10.4674 1.000
H6 H -9.4602 12.2215 8.6687 1.000
S2 S -6.9353 14.3445 1.0418 1.000
C21 C -4.8134 15.9342 1.2615 1.000
H18A H 3.1532 -1.5391 -2.4435 1.000
H18B H 2.6713 -2.9391 -3.3796 1.000
H21A H 3.2330 -5.2582 -0.5159 1.000
H21B H 1.6409 -4.6492 -0.8899 1.000
C22 C 2.6341 -5.5598 -2.5306 1.000
C13 C 6.6189 -3.4060 -4.6125 1.000
H14 H 5.4560 -1.9398 -3.5671 1.000
H16 H 6.0877 -5.6514 -1.5011 1.000
C17 C 6.9921 -5.4910 -3.4398 1.000
S1 S -0.4897 -3.6534 6.9866 1.000
H9A H 0.0718 -1.3585 7.4648 1.000

H9B H 1.2587 -2.4547 8.1345 1.000
H13 H 4.5908 8.4183 -5.4937 1.000
C9 C 7.3111 2.2487 -7.2603 1.000
O2 O 4.5842 3.1110 -6.9267 1.000
O3 O 6.6398 4.9881 -5.5179 1.000
N3 N 5.9505 6.6035 -6.8634 1.000
H4 H 2.1045 -1.1177 12.1073 1.000
H3 H 4.2625 -1.5971 10.9614 1.000
C1 C 4.6808 12.5444 -4.9647 1.000
C7 C 2.5130 13.3578 -4.6828 1.000
H6 H 0.5314 14.1966 -4.5603 1.000
H21A H 2.5735 16.4664 0.5159 1.000
H21B H 4.1657 15.8574 0.8899 1.000
C22 C 3.1724 16.7680 2.5306 1.000
C11 C 1.6548 15.3095 7.5443 1.000
N3 N 1.7654 15.8366 6.3655 1.000
O3 O -2.9914 17.4284 5.5179 1.000
N3 N -2.3021 15.8129 6.8634 1.000
H17 H -1.6146 17.6931 3.4099 1.000
S2 S 12.7418 -3.1363 -1.0418 1.000
C21 C 10.6199 -4.7260 -1.2615 1.000
O2 O 11.3452 -3.1110 6.9267 1.000
S1 S 7.4750 -3.6534 6.9866 1.000
H9A H 8.0365 -1.3585 7.4648 1.000
H9B H 9.2234 -2.4547 8.1345 1.000
C6 C 12.4604 -2.4257 4.0616 1.000
H5 H 13.5504 -2.1458 2.2366 1.000
C2 C 13.6396 1.4321 -4.0448 1.000
H3 H 14.2541 0.3781 -2.2676 1.000
C8 C 13.1106 2.5107 -6.0398 1.000
C14 C 11.6969 8.2767 -3.5377 1.000
C16 C 12.0580 6.1844 -2.3682 1.000
H19A H 15.3410 3.1016 2.1136 1.000
H19B H 15.1842 1.5393 2.8838 1.000
C20 C 14.9974 1.4948 0.7697 1.000
H22A H 13.9506 5.0976 3.2584 1.000
H22B H 12.2992 5.6030 2.9534 1.000
C23 C 13.7836 6.9719 2.2263 1.000
C3 C -4.4809 -1.0287 10.4681 1.000
C6 C -6.5226 0.4506 9.1674 1.000
O1 O -10.0772 10.3789 4.7962 1.000
C9 C -9.4693 8.9595 7.2603 1.000
C18 C -9.5503 6.7640 10.8223 1.000
C21 C -9.4945 4.5070 11.9674 1.000
C14 C -5.8903 2.9315 3.5377 1.000
C16 C -6.2514 5.0238 2.3682 1.000
C13 C -7.6891 17.0352 8.6164 1.000
C17 C -7.3158 14.9502 9.7892 1.000
H5 H -9.7708 11.3788 10.9923 1.000
O4 O -7.2550 15.1671 -0.0881 1.000
O5 O -7.1654 12.9286 0.9688 1.000
C15 C -7.8782 14.9491 2.4164 1.000
H21A H -5.3912 16.4664 0.5159 1.000

H21B H -3.7990 15.8574 0.8899 1.000
C22 C -4.7923 16.7680 2.5306 1.000
H22A H 1.9788 -5.0976 -3.2584 1.000
H22B H 3.6302 -5.6030 -2.9534 1.000
C23 C 2.1458 -6.9719 -2.2263 1.000
C12 C 7.1858 -4.6788 -4.5605 1.000
H13 H 6.7490 -2.7899 -5.4937 1.000
H17 H 7.4211 -6.4849 -3.4099 1.000
C10 C 0.7042 -4.8951 6.7363 1.000
S1 S 8.4544 3.6534 -6.9866 1.000
H9A H 7.8929 1.3585 -7.4648 1.000
H9B H 6.7060 2.4547 -8.1345 1.000
C10 C 7.2605 4.8951 -6.7363 1.000
N2 N 6.8873 5.8169 -7.5539 1.000
O1 O 5.7609 12.0375 -4.7962 1.000
N1 N 4.2840 13.1981 -6.1435 1.000
C8 C 2.9878 13.7189 -6.0398 1.000
H22A H 3.8278 16.3059 3.2584 1.000
H22B H 2.1763 16.8112 2.9534 1.000
C23 C 3.6607 18.1801 2.2263 1.000
C12 C 0.8426 15.7624 8.6684 1.000
C10 C -3.6121 17.5213 6.7363 1.000
N2 N -3.2389 16.5995 7.5539 1.000
O4 O 13.0615 -3.9589 0.0881 1.000
O5 O 12.9719 -1.7204 -0.9688 1.000
C15 C 13.6847 -3.7409 -2.4164 1.000
H21A H 11.1977 -5.2582 -0.5159 1.000
H21B H 9.6056 -4.6492 -0.8899 1.000
C22 C 10.5988 -5.5598 -2.5306 1.000
C10 C 8.6689 -4.8951 6.7363 1.000
H6 H 13.2398 -2.9884 4.5603 1.000
C1 C 14.8037 1.3362 -4.9647 1.000
O2 O 12.5489 3.1110 -6.9267 1.000
N1 N 14.4068 1.9899 -6.1435 1.000
C13 C 12.4254 7.8022 -4.6125 1.000
H14 H 11.2625 9.2684 -3.5671 1.000
H16 H 11.8942 5.5568 -1.5011 1.000
C17 C 12.7986 5.7172 -3.4398 1.000
H20A H 14.6280 2.1334 -0.0233 1.000
H20B H 14.4616 0.5537 0.7541 1.000
H20C H 16.0545 1.3111 0.6227 1.000
H23A H 13.1229 7.4342 1.5035 1.000
H23B H 14.7871 6.9275 1.8216 1.000
H23C H 13.7871 7.5568 3.1378 1.000
H3 H -3.7022 -1.5971 10.9614 1.000
C4 C -5.6928 -0.7551 11.1005 1.000
C5 C -6.6867 -0.0294 10.4674 1.000
H6 H -7.3020 1.0132 8.6687 1.000
S1 S -10.6126 7.5548 6.9866 1.000
H9A H -10.0511 9.8498 7.4648 1.000
H9B H -8.8641 8.7535 8.1345 1.000
H18A H -8.9966 7.6939 10.7854 1.000
H18B H -9.4785 6.2939 9.8493 1.000

C19 C -11.0050 7.0604 11.1277 1.000
H21A H -8.9168 3.9748 12.7130 1.000
H21B H -10.5089 4.5838 12.3390 1.000
C22 C -9.5156 3.6732 10.6984 1.000
C13 C -6.6189 3.4060 4.6125 1.000
H14 H -5.4560 1.9398 3.5671 1.000
H16 H -6.0877 5.6514 1.5011 1.000
C17 C -6.9921 5.4910 3.4398 1.000
H13 H -7.5590 17.6513 7.7352 1.000
C14 C -8.4176 17.5097 9.6913 1.000
C16 C -8.0565 15.4175 10.8607 1.000
H17 H -6.8868 13.9563 9.8191 1.000
C14 C -8.0485 14.1398 3.5377 1.000
C16 C -8.4096 16.2320 2.3682 1.000
H22A H -4.1369 16.3059 3.2584 1.000
H22B H -5.7884 16.8112 2.9534 1.000
C23 C -4.3040 18.1801 2.2263 1.000
H23A H 2.8065 -7.4342 -1.5035 1.000
H23B H 1.1423 -6.9275 -1.8216 1.000
H23C H 2.1423 -7.5568 -3.1378 1.000
C11 C 7.9981 -5.1317 -5.6846 1.000
O3 O 1.3249 -4.9881 5.5179 1.000
N2 N 1.0774 -5.8169 7.5539 1.000
C9 C 5.1530 13.4569 -7.2603 1.000
O2 O 2.4260 14.3192 -6.9267 1.000
H23A H 3.0001 18.6424 1.5035 1.000
H23B H 4.6643 18.1357 1.8216 1.000
H23C H 3.6642 18.7650 3.1378 1.000
C13 C 0.2756 17.0352 8.6164 1.000
C17 C 0.6489 14.9502 9.7892 1.000
S1 S -4.8061 18.7630 6.9866 1.000
C14 C 13.8551 -2.9315 -3.5377 1.000
C16 C 14.2161 -5.0238 -2.3682 1.000
H22A H 9.9435 -5.0976 -3.2584 1.000
H22B H 11.5949 -5.6030 -2.9534 1.000
C23 C 10.1105 -6.9719 -2.2263 1.000
O3 O 9.2896 -4.9881 5.5179 1.000
N2 N 9.0421 -5.8169 7.5539 1.000
O1 O 15.8837 0.8293 -4.7962 1.000
C9 C 15.2758 2.2487 -7.2603 1.000
C12 C 12.9924 6.5294 -4.5605 1.000
H13 H 12.5555 8.4183 -5.4937 1.000
H17 H 13.2277 4.7233 -3.4099 1.000
H4 H -5.8602 -1.1177 12.1073 1.000
H5 H -7.6126 0.1706 10.9923 1.000
C10 C -9.4186 6.3131 6.7363 1.000
H19A H -11.5614 6.1314 11.1154 1.000
H19B H -11.4045 7.6938 10.3452 1.000
C20 C -11.2177 7.7382 12.4593 1.000
H22A H -10.1710 4.1354 9.9705 1.000
H22B H -8.5196 3.6301 10.2756 1.000
C23 C -10.0040 2.2611 11.0026 1.000
C12 C -7.1858 4.6788 4.5605 1.000

H13 H -6.7490 2.7899 5.4937 1.000
H17 H -7.4211 6.4849 3.4099 1.000
H14 H -8.8519 18.5014 9.6619 1.000
C15 C -8.5879 16.7004 10.8125 1.000
H16 H -8.2203 14.7898 11.7279 1.000
C13 C -8.7770 14.6142 4.6125 1.000
H14 H -7.6142 13.1480 3.5671 1.000
H16 H -8.2458 16.8597 1.5011 1.000
C17 C -9.1503 16.6992 3.4398 1.000
H23A H -4.9646 18.6424 1.5035 1.000
H23B H -3.3004 18.1357 1.8216 1.000
H23C H -4.3005 18.7650 3.1378 1.000
O3 O 8.7979 -6.2202 -5.5179 1.000
N3 N 8.1086 -4.6047 -6.8634 1.000
C11 C 2.1248 -6.0765 5.6846 1.000
N3 N 2.0142 -6.6035 6.8634 1.000
S1 S 6.2963 14.8617 -6.9866 1.000
H9A H 5.7348 12.5667 -7.4648 1.000
H9B H 4.5478 13.6629 -8.1345 1.000
H13 H 0.4057 17.6513 7.7352 1.000
C14 C -0.4529 17.5097 9.6913 1.000
C16 C -0.0918 15.4175 10.8607 1.000
H17 H 1.0779 13.9563 9.8191 1.000
C9 C -3.6628 20.1678 7.2603 1.000
C13 C 14.5836 -3.4060 -4.6125 1.000
H14 H 13.4207 -1.9398 -3.5671 1.000
H16 H 14.0524 -5.6514 -1.5011 1.000
C17 C 14.9568 -5.4910 -3.4398 1.000
H23A H 10.7712 -7.4342 -1.5035 1.000
H23B H 9.1070 -6.9275 -1.8216 1.000
H23C H 10.1070 -7.5568 -3.1378 1.000
C11 C 10.0895 -6.0765 5.6846 1.000
N3 N 9.9789 -6.6035 6.8634 1.000
S1 S 16.4191 3.6534 -6.9866 1.000
H9A H 15.8576 1.3585 -7.4648 1.000
H9B H 14.6707 2.4547 -8.1345 1.000
C11 C 13.8046 6.0765 -5.6846 1.000
O3 O -8.7979 6.2202 5.5179 1.000
N2 N -9.0454 5.3913 7.5539 1.000
H20A H -10.8484 7.0997 13.2522 1.000
H20B H -10.6820 8.6793 12.4749 1.000
H20C H -12.2749 7.9219 12.6063 1.000
H23A H -9.3433 1.7988 11.7255 1.000
H23B H -11.0075 2.3055 11.4073 1.000
H23C H -10.0075 1.6762 10.0912 1.000
C11 C -7.9981 5.1317 5.6846 1.000
S2 S -9.5308 17.3049 12.1872 1.000
C12 C -9.3440 15.8871 4.5605 1.000
H13 H -8.9071 13.9981 5.4937 1.000
H17 H -9.5793 17.6931 3.4099 1.000
C10 C 9.4186 -6.3131 -6.7363 1.000
N2 N 9.0454 -5.3913 -7.5539 1.000
C12 C 2.9370 -6.5294 4.5605 1.000

C10 C 5.1023 16.1034 -6.7363 1.000
H14 H -0.8872 18.5014 9.6619 1.000
C15 C -0.6232 16.7004 10.8125 1.000
H16 H -0.2556 14.7898 11.7279 1.000
N1 N -2.7937 20.4265 6.1435 1.000
H9A H -4.2445 21.0580 7.4648 1.000
H9B H -3.0576 19.9617 8.1345 1.000
C12 C 15.1505 -4.6788 -4.5605 1.000
H13 H 14.7137 -2.7899 -5.4937 1.000
H17 H 15.3858 -6.4849 -3.4099 1.000
C12 C 10.9017 -6.5294 4.5605 1.000
C10 C 15.2252 4.8951 -6.7363 1.000
O3 O 14.6045 4.9881 -5.5179 1.000
N3 N 13.9152 6.6035 -6.8634 1.000
N3 N -8.1086 4.6047 6.8634 1.000
O4 O -9.2111 16.4823 13.3171 1.000
O5 O -9.3007 18.7208 12.2602 1.000
N4 N -11.1016 17.0805 11.8433 1.000
C11 C -10.1562 16.3399 5.6846 1.000
S1 S 10.6126 -7.5548 -6.9866 1.000
C13 C 3.5040 -7.8022 4.6125 1.000
C17 C 3.1308 -5.7172 3.4398 1.000
O3 O 4.4816 16.1963 -5.5179 1.000
N2 N 4.7291 17.0251 -7.5539 1.000
S2 S -1.5661 17.3049 12.1872 1.000
C1 C -3.1906 21.0802 4.9647 1.000
C8 C -1.4975 19.9057 6.0398 1.000
C11 C 15.9628 -5.1317 -5.6846 1.000
C13 C 11.4687 -7.8022 4.6125 1.000
C17 C 11.0955 -5.7172 3.4398 1.000
N2 N 14.8520 5.8169 -7.5539 1.000
C18 C -11.7085 17.9722 10.8223 1.000
C21 C -11.6527 15.7152 11.9674 1.000
O3 O -10.9561 17.4284 5.5179 1.000
N3 N -10.2668 15.8129 6.8634 1.000
C9 C 9.4693 -8.9595 -7.2603 1.000
H13 H 3.3739 -8.4183 5.4937 1.000
C14 C 4.2325 -8.2767 3.5377 1.000
C16 C 3.8714 -6.1844 2.3682 1.000
H17 H 2.7017 -4.7233 3.4099 1.000
C11 C 3.6817 17.2847 -5.6846 1.000
N3 N 3.7923 17.8118 -6.8634 1.000
O4 O -1.2464 16.4823 13.3171 1.000
O5 O -1.3360 18.7208 12.2602 1.000
N4 N -3.1369 17.0805 11.8433 1.000
O1 O -4.2707 21.5871 4.7962 1.000
C2 C -2.0265 20.9843 4.0448 1.000
O2 O -0.9358 19.3054 6.9267 1.000
C7 C -1.0227 20.2668 4.6828 1.000
O3 O 16.7626 -6.2202 -5.5179 1.000
N3 N 16.0733 -4.6047 -6.8634 1.000
H13 H 11.3386 -8.4183 5.4937 1.000
C14 C 12.1972 -8.2767 3.5377 1.000

C16 C 11.8361 -6.1844 2.3682 1.000
H17 H 10.6664 -4.7233 3.4099 1.000
H18A H -11.1548 18.9021 10.7854 1.000
H18B H -11.6367 17.5021 9.8493 1.000
C19 C -13.1632 18.2687 11.1277 1.000
H21A H -11.0749 15.1830 12.7130 1.000
H21B H -12.6671 15.7920 12.3390 1.000
C22 C -11.6738 14.8814 10.6984 1.000
C10 C -11.5768 17.5213 6.7363 1.000
N2 N -11.2036 16.5995 7.5539 1.000
N1 N 8.6003 -9.2183 -6.1435 1.000
H9A H 10.0511 -9.8498 -7.4648 1.000
H9B H 8.8641 -8.7535 -8.1345 1.000
H14 H 4.6669 -9.2684 3.5671 1.000
C15 C 4.4028 -7.4674 2.4164 1.000
H16 H 4.0352 -5.5568 1.5011 1.000
C12 C 2.8695 17.7376 -4.5605 1.000
C18 C -3.7438 17.9722 10.8223 1.000
C21 C -3.6880 15.7152 11.9674 1.000
C3 C -1.8624 21.4699 2.7609 1.000
C6 C 0.1794 19.9907 4.0616 1.000
C10 C 17.3833 -6.3131 -6.7363 1.000
N2 N 17.0101 -5.3913 -7.5539 1.000
H14 H 12.6316 -9.2684 3.5671 1.000
C15 C 12.3675 -7.4674 2.4164 1.000
H16 H 11.9999 -5.5568 1.5011 1.000
H19A H -13.7196 17.3397 11.1154 1.000
H19B H -13.5627 18.9020 10.3452 1.000
C20 C -13.3759 18.9464 12.4593 1.000
H22A H -12.3292 15.3436 9.9705 1.000
H22B H -10.6777 14.8383 10.2756 1.000
C23 C -12.1621 13.4694 11.0026 1.000
S1 S -12.7708 18.7630 6.9866 1.000
C1 C 8.9972 -9.8720 -4.9647 1.000
C8 C 7.3041 -8.6975 -6.0398 1.000
S2 S 5.3458 -8.0719 1.0418 1.000
C13 C 2.3025 19.0104 -4.6125 1.000
C17 C 2.6758 16.9254 -3.4398 1.000
H18A H -3.1901 18.9021 10.7854 1.000
H18B H -3.6720 17.5021 9.8493 1.000
C19 C -5.1985 18.2687 11.1277 1.000
H21A H -3.1102 15.1830 12.7130 1.000
H21B H -4.7024 15.7920 12.3390 1.000
C22 C -3.7091 14.8814 10.6984 1.000
H3 H -2.6411 22.0383 2.2676 1.000
C4 C -0.6504 21.1964 2.1284 1.000
C5 C 0.3434 20.4706 2.7615 1.000
H6 H 0.9588 19.4280 4.5603 1.000
S1 S 18.5773 -7.5548 -6.9866 1.000
S2 S 13.3105 -8.0719 1.0418 1.000
H20A H -13.0066 18.3079 13.2522 1.000
H20B H -12.8402 19.8875 12.4749 1.000
H20C H -14.4331 19.1302 12.6063 1.000

H23A H -11.5015 13.0070 11.7255 1.000
H23B H -13.1657 13.5137 11.4073 1.000
H23C H -12.1656 12.8844 10.0912 1.000
C9 C -11.6275 20.1678 7.2603 1.000
O1 O 10.0772 -10.3789 -4.7962 1.000
C2 C 7.8331 -9.7761 -4.0448 1.000
O2 O 6.7423 -8.0972 -6.9267 1.000
C7 C 6.8293 -9.0586 -4.6828 1.000
O4 O 5.0260 -7.2493 -0.0881 1.000
O5 O 5.1157 -9.4878 0.9688 1.000
N4 N 6.9166 -7.8475 1.3856 1.000
H13 H 2.4327 19.6265 -5.4937 1.000
C14 C 1.5740 19.4849 -3.5377 1.000
C16 C 1.9351 17.3927 -2.3682 1.000
H17 H 3.1048 15.9315 -3.4099 1.000
H19A H -5.7549 17.3397 11.1154 1.000
H19B H -5.5980 18.9020 10.3452 1.000
C20 C -5.4112 18.9464 12.4593 1.000
H22A H -4.3645 15.3436 9.9705 1.000
H22B H -2.7130 14.8383 10.2756 1.000
C23 C -4.1974 13.4694 11.0026 1.000
H4 H -0.4830 21.5590 1.1217 1.000
H5 H 1.2694 20.2707 2.2366 1.000
C9 C 17.4340 -8.9595 -7.2603 1.000
O4 O 12.9907 -7.2493 -0.0881 1.000
O5 O 13.0804 -9.4878 0.9688 1.000
N4 N 14.8813 -7.8475 1.3856 1.000
N1 N -10.7584 20.4265 6.1435 1.000
H9A H -12.2092 21.0580 7.4648 1.000
H9B H -11.0223 19.9617 8.1345 1.000
C3 C 7.6689 -10.2617 -2.7609 1.000
C6 C 5.6272 -8.7825 -4.0616 1.000
C18 C 7.5234 -8.7391 2.4066 1.000
C21 C 7.4676 -6.4822 1.2615 1.000
H14 H 1.1397 20.4766 -3.5671 1.000
C15 C 1.4037 18.6756 -2.4164 1.000
H16 H 1.7714 16.7650 -1.5011 1.000
H20A H -5.0419 18.3079 13.2522 1.000
H20B H -4.8755 19.8875 12.4749 1.000
H20C H -6.4683 19.1302 12.6063 1.000
H23A H -3.5368 13.0070 11.7255 1.000
H23B H -5.2010 13.5137 11.4073 1.000
H23C H -4.2009 12.8844 10.0912 1.000
N1 N 16.5650 -9.2183 -6.1435 1.000
H9A H 18.0158 -9.8498 -7.4648 1.000
H9B H 16.8288 -8.7535 -8.1345 1.000
C18 C 15.4881 -8.7391 2.4066 1.000
C21 C 15.4323 -6.4822 1.2615 1.000
C1 C -11.1553 21.0802 4.9647 1.000
C8 C -9.4622 19.9057 6.0398 1.000
H3 H 8.4476 -10.8301 -2.2676 1.000
C4 C 6.4570 -9.9882 -2.1284 1.000
C5 C 5.4631 -9.2624 -2.7615 1.000

H6 H 4.8478 -8.2198 -4.5603 1.000
H18A H 6.9697 -9.6691 2.4435 1.000
H18B H 7.4516 -8.2691 3.3796 1.000
C19 C 8.9781 -9.0356 2.1013 1.000
H21A H 6.8898 -5.9500 0.5159 1.000
H21B H 8.4820 -6.5590 0.8899 1.000
C22 C 7.4887 -5.6484 2.5306 1.000
S2 S 0.4608 19.2801 -1.0418 1.000
C1 C 16.9619 -9.8720 -4.9647 1.000
C8 C 15.2688 -8.6975 -6.0398 1.000
H18A H 14.9344 -9.6691 2.4435 1.000
H18B H 15.4163 -8.2691 3.3796 1.000
C19 C 16.9428 -9.0356 2.1013 1.000
H21A H 14.8545 -5.9500 0.5159 1.000
H21B H 16.4467 -6.5590 0.8899 1.000
C22 C 15.4534 -5.6484 2.5306 1.000
O1 O -12.2354 21.5871 4.7962 1.000
C2 C -9.9912 20.9843 4.0448 1.000
O2 O -8.9005 19.3054 6.9267 1.000
C7 C -8.9874 20.2668 4.6828 1.000
H4 H 6.2896 -10.3508 -1.1217 1.000
H5 H 4.5372 -9.0625 -2.2366 1.000
H19A H 9.5345 -8.1066 2.1136 1.000
H19B H 9.3776 -9.6689 2.8838 1.000
C20 C 9.1908 -9.7134 0.7697 1.000
H22A H 8.1441 -6.1106 3.2584 1.000
H22B H 6.4926 -5.6053 2.9534 1.000
C23 C 7.9771 -4.2363 2.2263 1.000
O4 O 0.7805 18.4575 0.0881 1.000
O5 O 0.6909 20.6960 -0.9688 1.000
N4 N -1.1100 19.0557 -1.3856 1.000
O1 O 18.0419 -10.3789 -4.7962 1.000
C2 C 15.7978 -9.7761 -4.0448 1.000
O2 O 14.7070 -8.0972 -6.9267 1.000
C7 C 14.7940 -9.0586 -4.6828 1.000
H19A H 17.4992 -8.1066 2.1136 1.000
H19B H 17.3423 -9.6689 2.8838 1.000
C20 C 17.1555 -9.7134 0.7697 1.000
H22A H 16.1088 -6.1106 3.2584 1.000
H22B H 14.4573 -5.6053 2.9534 1.000
C23 C 15.9418 -4.2363 2.2263 1.000
C3 C -9.8271 21.4699 2.7609 1.000
C6 C -7.7853 19.9907 4.0616 1.000
H20A H 8.8215 -9.0748 -0.0233 1.000
H20B H 8.6551 -10.6545 0.7541 1.000
H20C H 10.2480 -9.8971 0.6227 1.000
H23A H 7.3164 -3.7740 1.5035 1.000
H23B H 8.9806 -4.2807 1.8216 1.000
H23C H 7.9805 -3.6514 3.1378 1.000
C18 C -1.7168 19.9474 -2.4066 1.000
C21 C -1.6611 17.6904 -1.2615 1.000
C3 C 15.6336 -10.2617 -2.7609 1.000
C6 C 13.5919 -8.7825 -4.0616 1.000

H20A H 16.7862 -9.0748 -0.0233 1.000
H20B H 16.6198 -10.6545 0.7541 1.000
H20C H 18.2127 -9.8971 0.6227 1.000
H23A H 15.2811 -3.7740 1.5035 1.000
H23B H 16.9453 -4.2807 1.8216 1.000
H23C H 15.9452 -3.6514 3.1378 1.000
H3 H -10.6058 22.0383 2.2676 1.000
C4 C -8.6151 21.1964 2.1284 1.000
C5 C -7.6213 20.4706 2.7615 1.000
H6 H -7.0059 19.4280 4.5603 1.000
H18A H -1.1631 20.8773 -2.4435 1.000
H18B H -1.6451 19.4773 -3.3796 1.000
C19 C -3.1715 20.2438 -2.1013 1.000
H21A H -1.0833 17.1582 -0.5159 1.000
H21B H -2.6754 17.7672 -0.8899 1.000
C22 C -1.6822 16.8566 -2.5306 1.000
H3 H 16.4123 -10.8301 -2.2676 1.000
C4 C 14.4217 -9.9882 -2.1284 1.000
C5 C 13.4278 -9.2624 -2.7615 1.000
H6 H 12.8125 -8.2198 -4.5603 1.000
H4 H -8.4477 21.5590 1.1217 1.000
H5 H -6.6953 20.2707 2.2366 1.000
H19A H -3.7280 19.3148 -2.1136 1.000
H19B H -3.5711 20.8772 -2.8838 1.000
C20 C -3.3843 20.9216 -0.7697 1.000
H22A H -2.3375 17.3188 -3.2584 1.000
H22B H -0.6861 16.8135 -2.9534 1.000
C23 C -2.1705 15.4445 -2.2263 1.000
H4 H 14.2543 -10.3508 -1.1217 1.000
H5 H 12.5019 -9.0625 -2.2366 1.000
H20A H -3.0149 20.2831 0.0233 1.000
H20B H -2.8485 21.8627 -0.7540 1.000
H20C H -4.4414 21.1053 -0.6227 1.000
H23A H -1.5098 14.9822 -1.5035 1.000
H23B H -3.1741 15.4889 -1.8216 1.000
H23C H -2.1740 14.8596 -3.1378 1.000

All Atoms [16 molecules]
928 atoms, Cartesian coordinates
Label Symbol x y z Occ.

[x, y, z]
H4 H 1.9733 12.0657 -1.1217 1.000
C4 C 2.1406 12.4283 -2.1284 1.000
C3 C 3.3526 12.1547 -2.7609 1.000
C5 C 1.1468 13.1540 -2.7615 1.000
C2 C 3.5168 12.6403 -4.0448 1.000
H3 H 4.1313 11.5863 -2.2676 1.000
H5 H 0.2209 13.3540 -2.2366 1.000
C6 C 1.3109 13.6340 -4.0616 1.000
C1 C 4.6808 12.5444 -4.9647 1.000
C7 C 2.5130 13.3578 -4.6828 1.000

H6 H 0.5314 14.1966 -4.5603 1.000
O1 O 5.7609 12.0375 -4.7962 1.000
N1 N 4.2840 13.1981 -6.1435 1.000
C8 C 2.9878 13.7189 -6.0398 1.000
C9 C 5.1530 13.4569 -7.2603 1.000
O2 O 2.4260 14.3192 -6.9267 1.000
S1 S 6.2963 14.8617 -6.9866 1.000
H9A H 5.7348 12.5667 -7.4648 1.000
H9B H 4.5478 13.6629 -8.1345 1.000
C10 C 5.1023 16.1034 -6.7363 1.000
O3 O 4.4816 16.1963 -5.5179 1.000
N2 N 4.7291 17.0251 -7.5539 1.000
C11 C 3.6817 17.2847 -5.6846 1.000
N3 N 3.7923 17.8118 -6.8634 1.000
C12 C 2.8695 17.7376 -4.5605 1.000
C13 C 2.3025 19.0104 -4.6125 1.000
C17 C 2.6758 16.9254 -3.4398 1.000
H13 H 2.4327 19.6265 -5.4937 1.000
C14 C 1.5740 19.4849 -3.5377 1.000
C16 C 1.9351 17.3927 -2.3682 1.000
H17 H 3.1048 15.9315 -3.4099 1.000
H14 H 1.1397 20.4766 -3.5671 1.000
C15 C 1.4037 18.6756 -2.4164 1.000
H16 H 1.7714 16.7650 -1.5011 1.000
S2 S 0.4608 19.2801 -1.0418 1.000
O4 O 0.7805 18.4575 0.0881 1.000
O5 O 0.6909 20.6960 -0.9688 1.000
N4 N -1.1100 19.0557 -1.3856 1.000
C18 C -1.7168 19.9474 -2.4066 1.000
C21 C -1.6611 17.6904 -1.2615 1.000
H18A H -1.1631 20.8773 -2.4435 1.000
H18B H -1.6451 19.4773 -3.3796 1.000
C19 C -3.1715 20.2438 -2.1013 1.000
H21A H -1.0833 17.1582 -0.5159 1.000
H21B H -2.6754 17.7672 -0.8899 1.000
C22 C -1.6822 16.8566 -2.5306 1.000
H19A H -3.7280 19.3148 -2.1136 1.000
H19B H -3.5711 20.8772 -2.8838 1.000
C20 C -3.3843 20.9216 -0.7697 1.000
H22A H -2.3375 17.3188 -3.2584 1.000
H22B H -0.6861 16.8135 -2.9534 1.000
C23 C -2.1705 15.4445 -2.2263 1.000
H20A H -3.0149 20.2831 0.0233 1.000
H20B H -2.8485 21.8627 -0.7540 1.000
H20C H -4.4414 21.1053 -0.6227 1.000
H23A H -1.5098 14.9822 -1.5035 1.000
H23B H -3.1741 15.4889 -1.8216 1.000
H23C H -2.1740 14.8596 -3.1378 1.000

[x, y, z]
H20C H 5.6814 9.8971 -0.6227 1.000
C20 C 6.7386 9.7134 -0.7697 1.000
H20A H 7.1079 9.0748 0.0233 1.000

C19 C 6.9513 9.0356 -2.1013 1.000
H20B H 7.2743 10.6545 -0.7540 1.000
C18 C 8.4060 8.7391 -2.4066 1.000
H19A H 6.3949 8.1066 -2.1136 1.000
H19B H 6.5518 9.6689 -2.8838 1.000
N4 N 9.0128 7.8475 -1.3856 1.000
H18A H 8.9597 9.6691 -2.4435 1.000
H18B H 8.4778 8.2691 -3.3796 1.000
C21 C 8.4618 6.4822 -1.2615 1.000
S2 S 10.5836 8.0719 -1.0418 1.000
H21A H 9.0396 5.9500 -0.5159 1.000
H21B H 7.4474 6.5590 -0.8899 1.000
C22 C 8.4407 5.6484 -2.5306 1.000
O4 O 10.9034 7.2493 0.0881 1.000
O5 O 10.8137 9.4878 -0.9688 1.000
C15 C 11.5266 7.4674 -2.4164 1.000
C23 C 7.9523 4.2363 -2.2263 1.000
H22A H 7.7853 6.1106 -3.2584 1.000
H22B H 9.4368 5.6053 -2.9534 1.000
C14 C 11.6969 8.2767 -3.5377 1.000
C16 C 12.0580 6.1844 -2.3682 1.000
H23B H 6.9488 4.2807 -1.8216 1.000
H23A H 8.6130 3.7740 -1.5035 1.000
H23C H 7.9489 3.6514 -3.1378 1.000
C13 C 12.4254 7.8022 -4.6125 1.000
H14 H 11.2625 9.2684 -3.5671 1.000
H16 H 11.8942 5.5568 -1.5011 1.000
C17 C 12.7986 5.7172 -3.4398 1.000
C12 C 12.9924 6.5294 -4.5605 1.000
H13 H 12.5555 8.4183 -5.4937 1.000
H17 H 13.2277 4.7233 -3.4099 1.000
C11 C 13.8046 6.0765 -5.6846 1.000
O3 O 14.6045 4.9881 -5.5179 1.000
N3 N 13.9152 6.6035 -6.8634 1.000
C10 C 15.2252 4.8951 -6.7363 1.000
N2 N 14.8520 5.8169 -7.5539 1.000
S1 S 16.4191 3.6534 -6.9866 1.000
C9 C 15.2758 2.2487 -7.2603 1.000
N1 N 14.4068 1.9899 -6.1435 1.000
H9A H 15.8576 1.3585 -7.4648 1.000
H9B H 14.6707 2.4547 -8.1345 1.000
C8 C 13.1106 2.5107 -6.0398 1.000
C1 C 14.8037 1.3362 -4.9647 1.000
C7 C 12.6358 2.1496 -4.6828 1.000
O2 O 12.5489 3.1110 -6.9267 1.000
C2 C 13.6396 1.4321 -4.0448 1.000
O1 O 15.8837 0.8293 -4.7962 1.000
C6 C 11.4337 2.4257 -4.0616 1.000
C3 C 13.4754 0.9465 -2.7609 1.000
C5 C 11.2697 1.9458 -2.7615 1.000
H6 H 10.6543 2.9884 -4.5603 1.000
C4 C 12.2635 1.2200 -2.1284 1.000
H3 H 14.2541 0.3781 -2.2676 1.000

H5 H 10.3437 2.1458 -2.2366 1.000
H4 H 12.0961 0.8575 -1.1217 1.000

[-x, -y, -z]
O4 O 2.8679 3.9589 -0.0881 1.000
S2 S 3.1876 3.1363 1.0418 1.000
O5 O 2.9575 1.7204 0.9688 1.000
N4 N 4.7584 3.3607 1.3856 1.000
C15 C 2.2447 3.7409 2.4164 1.000
C18 C 5.3652 2.4691 2.4066 1.000
C21 C 5.3095 4.7260 1.2615 1.000
C14 C 2.0744 2.9315 3.5377 1.000
C16 C 1.7133 5.0238 2.3682 1.000
H18A H 4.8115 1.5391 2.4435 1.000
H18B H 5.2934 2.9391 3.3796 1.000
C19 C 6.8199 2.1726 2.1013 1.000
H21A H 4.7317 5.2582 0.5159 1.000
H21B H 6.3238 4.6492 0.8899 1.000
C22 C 5.3306 5.5598 2.5306 1.000
C13 C 1.3458 3.4060 4.6125 1.000
H14 H 2.5087 1.9398 3.5671 1.000
H16 H 1.8770 5.6514 1.5011 1.000
C17 C 0.9726 5.4910 3.4398 1.000
C20 C 7.0327 1.4948 0.7697 1.000
H19A H 7.3763 3.1016 2.1136 1.000
H19B H 7.2195 1.5393 2.8838 1.000
H22A H 5.9859 5.0976 3.2584 1.000
H22B H 4.3345 5.6030 2.9534 1.000
C23 C 5.8189 6.9719 2.2263 1.000
C12 C 0.7789 4.6788 4.5605 1.000
H13 H 1.2157 2.7899 5.4937 1.000
H17 H 0.5436 6.4849 3.4099 1.000
H20A H 6.6633 2.1334 -0.0233 1.000
H20B H 6.4969 0.5537 0.7541 1.000
H20C H 8.0898 1.3111 0.6227 1.000
H23A H 5.1582 7.4342 1.5035 1.000
H23C H 5.8224 7.5568 3.1378 1.000
H23B H 6.8224 6.9275 1.8216 1.000
C11 C -0.0334 5.1317 5.6846 1.000
O3 O -0.8332 6.2202 5.5179 1.000
N3 N -0.1439 4.6047 6.8634 1.000
C10 C -1.4539 6.3131 6.7363 1.000
N2 N -1.0807 5.3913 7.5539 1.000
S1 S -2.6479 7.5548 6.9866 1.000
C9 C -1.5046 8.9595 7.2603 1.000
N1 N -0.6356 9.2183 6.1435 1.000
H9A H -2.0864 9.8498 7.4648 1.000
H9B H -0.8994 8.7535 8.1345 1.000
C1 C -1.0325 9.8720 4.9647 1.000
C8 C 0.6606 8.6975 6.0398 1.000
O1 O -2.1125 10.3789 4.7962 1.000
C2 C 0.1316 9.7761 4.0448 1.000
O2 O 1.2224 8.0972 6.9267 1.000

C7 C 1.1354 9.0586 4.6828 1.000
C3 C 0.2958 10.2617 2.7609 1.000
C6 C 2.3375 8.7825 4.0616 1.000
H3 H -0.4829 10.8301 2.2676 1.000
C4 C 1.5077 9.9882 2.1284 1.000
C5 C 2.5016 9.2624 2.7615 1.000
H6 H 3.1169 8.2198 4.5603 1.000
H4 H 1.6751 10.3508 1.1217 1.000
H5 H 3.4275 9.0625 2.2366 1.000

[-x, -y, -z]
C2 C 2.2898 -1.4321 4.0448 1.000
C3 C 2.4540 -0.9465 2.7609 1.000
C1 C 1.1257 -1.3362 4.9647 1.000
C7 C 3.2936 -2.1496 4.6828 1.000
H3 H 1.6753 -0.3781 2.2676 1.000
C4 C 3.6659 -1.2200 2.1284 1.000
O1 O 0.0457 -0.8293 4.7962 1.000
N1 N 1.5226 -1.9899 6.1435 1.000
C6 C 4.4957 -2.4257 4.0616 1.000
C8 C 2.8188 -2.5107 6.0398 1.000
H4 H 3.8333 -0.8575 1.1217 1.000
C5 C 4.6597 -1.9458 2.7615 1.000
C9 C 0.6536 -2.2487 7.2603 1.000
H6 H 5.2751 -2.9884 4.5603 1.000
O2 O 3.3805 -3.1110 6.9267 1.000
H5 H 5.5857 -2.1458 2.2366 1.000
S1 S -0.4897 -3.6534 6.9866 1.000
H9A H 0.0718 -1.3585 7.4648 1.000
H9B H 1.2587 -2.4547 8.1345 1.000
C10 C 0.7042 -4.8951 6.7363 1.000
O3 O 1.3249 -4.9881 5.5179 1.000
N2 N 1.0774 -5.8169 7.5539 1.000
C11 C 2.1248 -6.0765 5.6846 1.000
N3 N 2.0142 -6.6035 6.8634 1.000
C12 C 2.9370 -6.5294 4.5605 1.000
C13 C 3.5040 -7.8022 4.6125 1.000
C17 C 3.1308 -5.7172 3.4398 1.000
H13 H 3.3739 -8.4183 5.4937 1.000
C14 C 4.2325 -8.2767 3.5377 1.000
C16 C 3.8714 -6.1844 2.3682 1.000
H17 H 2.7017 -4.7233 3.4099 1.000
H14 H 4.6669 -9.2684 3.5671 1.000
C15 C 4.4028 -7.4674 2.4164 1.000
H16 H 4.0352 -5.5568 1.5011 1.000
S2 S 5.3458 -8.0719 1.0418 1.000
O4 O 5.0260 -7.2493 -0.0881 1.000
O5 O 5.1157 -9.4878 0.9688 1.000
N4 N 6.9166 -7.8475 1.3856 1.000
C18 C 7.5234 -8.7391 2.4066 1.000
C21 C 7.4676 -6.4822 1.2615 1.000
H18A H 6.9697 -9.6691 2.4435 1.000
H18B H 7.4516 -8.2691 3.3796 1.000

C19 C 8.9781 -9.0356 2.1013 1.000
H21A H 6.8898 -5.9500 0.5159 1.000
H21B H 8.4820 -6.5590 0.8899 1.000
C22 C 7.4887 -5.6484 2.5306 1.000
H19A H 9.5345 -8.1066 2.1136 1.000
H19B H 9.3776 -9.6689 2.8838 1.000
C20 C 9.1908 -9.7134 0.7697 1.000
H22A H 8.1441 -6.1106 3.2584 1.000
H22B H 6.4926 -5.6053 2.9534 1.000
C23 C 7.9771 -4.2363 2.2263 1.000
H20A H 8.8215 -9.0748 -0.0233 1.000
H20B H 8.6551 -10.6545 0.7541 1.000
H20C H 10.2480 -9.8971 0.6227 1.000
H23A H 7.3164 -3.7740 1.5035 1.000
H23B H 8.9806 -4.2807 1.8216 1.000
H23C H 7.9805 -3.6514 3.1378 1.000

[-x, -y, -z]
S1 S 5.3168 7.5548 6.9866 1.000
C10 C 6.5108 6.3131 6.7363 1.000
C9 C 6.4601 8.9595 7.2603 1.000
O3 O 7.1315 6.2202 5.5179 1.000
N2 N 6.8840 5.3913 7.5539 1.000
N1 N 7.3291 9.2183 6.1435 1.000
H9A H 5.8783 9.8498 7.4648 1.000
H9B H 7.0653 8.7535 8.1345 1.000
C11 C 7.9313 5.1317 5.6846 1.000
N3 N 7.8208 4.6047 6.8634 1.000
C1 C 6.9322 9.8720 4.9647 1.000
C8 C 8.6253 8.6975 6.0398 1.000
C12 C 8.7436 4.6788 4.5605 1.000
O1 O 5.8522 10.3789 4.7962 1.000
C2 C 8.0963 9.7761 4.0448 1.000
C7 C 9.1001 9.0586 4.6828 1.000
O2 O 9.1871 8.0972 6.9267 1.000
C13 C 9.3105 3.4060 4.6125 1.000
C17 C 8.9373 5.4910 3.4398 1.000
C3 C 8.2605 10.2617 2.7609 1.000
C6 C 10.3022 8.7825 4.0616 1.000
H13 H 9.1804 2.7899 5.4937 1.000
C14 C 10.0391 2.9315 3.5377 1.000
C16 C 9.6780 5.0238 2.3682 1.000
H17 H 8.5083 6.4849 3.4099 1.000
H3 H 7.4818 10.8301 2.2676 1.000
C4 C 9.4724 9.9882 2.1284 1.000
C5 C 10.4663 9.2624 2.7615 1.000
H6 H 11.0816 8.2198 4.5603 1.000
H14 H 10.4734 1.9398 3.5671 1.000
C15 C 10.2094 3.7409 2.4164 1.000
H16 H 9.8417 5.6514 1.5011 1.000
H4 H 9.6398 10.3508 1.1217 1.000
H5 H 11.3922 9.0625 2.2366 1.000
S2 S 11.1523 3.1363 1.0418 1.000

O5 O 10.9222 1.7204 0.9688 1.000
O4 O 10.8326 3.9589 -0.0881 1.000
N4 N 12.7231 3.3607 1.3856 1.000
C18 C 13.3299 2.4691 2.4066 1.000
C21 C 13.2742 4.7260 1.2615 1.000
H18A H 12.7762 1.5391 2.4435 1.000
H18B H 13.2581 2.9391 3.3796 1.000
C19 C 14.7846 2.1726 2.1013 1.000
H21A H 12.6964 5.2582 0.5159 1.000
H21B H 14.2885 4.6492 0.8899 1.000
C22 C 13.2953 5.5598 2.5306 1.000
H19A H 15.3410 3.1016 2.1136 1.000
H19B H 15.1842 1.5393 2.8838 1.000
C20 C 14.9974 1.4948 0.7697 1.000
H22A H 13.9506 5.0976 3.2584 1.000
H22B H 12.2992 5.6030 2.9534 1.000
C23 C 13.7836 6.9719 2.2263 1.000
H20A H 14.6280 2.1334 -0.0233 1.000
H20B H 14.4616 0.5537 0.7541 1.000
H20C H 16.0545 1.3111 0.6227 1.000
H23A H 13.1229 7.4342 1.5035 1.000
H23B H 14.7871 6.9275 1.8216 1.000
H23C H 13.7871 7.5568 3.1378 1.000

[-x, -y, -z]
O1 O 8.0104 -0.8293 4.7962 1.000
C1 C 9.0904 -1.3362 4.9647 1.000
N1 N 9.4873 -1.9899 6.1435 1.000
C2 C 10.2545 -1.4321 4.0448 1.000
C8 C 10.7835 -2.5107 6.0398 1.000
C9 C 8.6183 -2.2487 7.2603 1.000
C3 C 10.4187 -0.9465 2.7609 1.000
C7 C 11.2583 -2.1496 4.6828 1.000
O2 O 11.3452 -3.1110 6.9267 1.000
S1 S 7.4750 -3.6534 6.9866 1.000
H9A H 8.0365 -1.3585 7.4648 1.000
H9B H 9.2234 -2.4547 8.1345 1.000
H3 H 9.6400 -0.3781 2.2676 1.000
C4 C 11.6306 -1.2200 2.1284 1.000
C6 C 12.4604 -2.4257 4.0616 1.000
C10 C 8.6689 -4.8951 6.7363 1.000
H4 H 11.7980 -0.8575 1.1217 1.000
C5 C 12.6244 -1.9458 2.7615 1.000
H6 H 13.2398 -2.9884 4.5603 1.000
O3 O 9.2896 -4.9881 5.5179 1.000
N2 N 9.0421 -5.8169 7.5539 1.000
H5 H 13.5504 -2.1458 2.2366 1.000
C11 C 10.0895 -6.0765 5.6846 1.000
N3 N 9.9789 -6.6035 6.8634 1.000
C12 C 10.9017 -6.5294 4.5605 1.000
C13 C 11.4687 -7.8022 4.6125 1.000
C17 C 11.0955 -5.7172 3.4398 1.000
H13 H 11.3386 -8.4183 5.4937 1.000

C14 C 12.1972 -8.2767 3.5377 1.000
C16 C 11.8361 -6.1844 2.3682 1.000
H17 H 10.6664 -4.7233 3.4099 1.000
H14 H 12.6316 -9.2684 3.5671 1.000
C15 C 12.3675 -7.4674 2.4164 1.000
H16 H 11.9999 -5.5568 1.5011 1.000
S2 S 13.3105 -8.0719 1.0418 1.000
O4 O 12.9907 -7.2493 -0.0881 1.000
O5 O 13.0804 -9.4878 0.9688 1.000
N4 N 14.8813 -7.8475 1.3856 1.000
C18 C 15.4881 -8.7391 2.4066 1.000
C21 C 15.4323 -6.4822 1.2615 1.000
H18A H 14.9344 -9.6691 2.4435 1.000
H18B H 15.4163 -8.2691 3.3796 1.000
C19 C 16.9428 -9.0356 2.1013 1.000
H21A H 14.8545 -5.9500 0.5159 1.000
H21B H 16.4467 -6.5590 0.8899 1.000
C22 C 15.4534 -5.6484 2.5306 1.000
H19A H 17.4992 -8.1066 2.1136 1.000
H19B H 17.3423 -9.6689 2.8838 1.000
C20 C 17.1555 -9.7134 0.7697 1.000
H22A H 16.1088 -6.1106 3.2584 1.000
H22B H 14.4573 -5.6053 2.9534 1.000
C23 C 15.9418 -4.2363 2.2263 1.000
H20A H 16.7862 -9.0748 -0.0233 1.000
H20B H 16.6198 -10.6545 0.7541 1.000
H20C H 18.2127 -9.8971 0.6227 1.000
H23A H 15.2811 -3.7740 1.5035 1.000
H23B H 16.9453 -4.2807 1.8216 1.000
H23C H 15.9452 -3.6514 3.1378 1.000

[-x, -y, -z]
O5 O 0.7993 12.9286 0.9688 1.000
S2 S 1.0294 14.3445 1.0418 1.000
C15 C 0.0865 14.9491 2.4164 1.000
O4 O 0.7097 15.1671 -0.0881 1.000
N4 N 2.6002 14.5689 1.3856 1.000
C14 C -0.0838 14.1398 3.5377 1.000
C16 C -0.4449 16.2320 2.3682 1.000
C18 C 3.2071 13.6773 2.4066 1.000
C21 C 3.1513 15.9342 1.2615 1.000
H14 H 0.3505 13.1480 3.5671 1.000
C13 C -0.8123 14.6142 4.6125 1.000
C17 C -1.1856 16.6992 3.4398 1.000
H16 H -0.2811 16.8597 1.5011 1.000
H18A H 2.6534 12.7473 2.4435 1.000
H18B H 3.1353 14.1473 3.3796 1.000
C19 C 4.6618 13.3808 2.1013 1.000
H21A H 2.5735 16.4664 0.5159 1.000
H21B H 4.1657 15.8574 0.8899 1.000
C22 C 3.1724 16.7680 2.5306 1.000
C12 C -1.3793 15.8871 4.5605 1.000
H13 H -0.9424 13.9981 5.4937 1.000

H17 H -1.6146 17.6931 3.4099 1.000
C20 C 4.8745 12.7030 0.7697 1.000
H19A H 5.2182 14.3098 2.1136 1.000
H19B H 5.0613 12.7475 2.8838 1.000
H22A H 3.8278 16.3059 3.2584 1.000
H22B H 2.1763 16.8112 2.9534 1.000
C23 C 3.6607 18.1801 2.2263 1.000
C11 C -2.1915 16.3399 5.6846 1.000
H20B H 4.3388 11.7620 0.7541 1.000
H20A H 4.5052 13.3416 -0.0233 1.000
H20C H 5.9316 12.5193 0.6227 1.000
H23A H 3.0001 18.6424 1.5035 1.000
H23B H 4.6643 18.1357 1.8216 1.000
H23C H 3.6642 18.7650 3.1378 1.000
O3 O -2.9914 17.4284 5.5179 1.000
N3 N -2.3021 15.8129 6.8634 1.000
C10 C -3.6121 17.5213 6.7363 1.000
N2 N -3.2389 16.5995 7.5539 1.000
S1 S -4.8061 18.7630 6.9866 1.000
C9 C -3.6628 20.1678 7.2603 1.000
N1 N -2.7937 20.4265 6.1435 1.000
H9A H -4.2445 21.0580 7.4648 1.000
H9B H -3.0576 19.9617 8.1345 1.000
C1 C -3.1906 21.0802 4.9647 1.000
C8 C -1.4975 19.9057 6.0398 1.000
O1 O -4.2707 21.5871 4.7962 1.000
C2 C -2.0265 20.9843 4.0448 1.000
O2 O -0.9358 19.3054 6.9267 1.000
C7 C -1.0227 20.2668 4.6828 1.000
C3 C -1.8624 21.4699 2.7609 1.000
C6 C 0.1794 19.9907 4.0616 1.000
H3 H -2.6411 22.0383 2.2676 1.000
C4 C -0.6504 21.1964 2.1284 1.000
C5 C 0.3434 20.4706 2.7615 1.000
H6 H 0.9588 19.4280 4.5603 1.000
H4 H -0.4830 21.5590 1.1217 1.000
H5 H 1.2694 20.2707 2.2366 1.000

[x, y, z]
O1 O 3.7340 10.0623 8.4328 1.000
C1 C 2.6539 10.5692 8.2643 1.000
N1 N 2.2570 11.2229 7.0854 1.000
C2 C 1.4898 10.6651 9.1842 1.000
C8 C 0.9608 11.7438 7.1891 1.000
C9 C 3.1261 11.4817 5.9686 1.000
C3 C 1.3256 10.1795 10.4681 1.000
C7 C 0.4860 11.3827 8.5462 1.000
O2 O 0.3991 12.3440 6.3023 1.000
H9A H 3.7078 10.5915 5.7641 1.000
H9B H 2.5209 11.6878 5.0945 1.000
S1 S 4.2693 12.8865 6.2423 1.000
C4 C 0.1137 10.4531 11.1005 1.000
H3 H 2.1044 9.6112 10.9614 1.000

C6 C -0.7161 11.6588 9.1674 1.000
C10 C 3.0754 14.1282 6.4926 1.000
C5 C -0.8801 11.1789 10.4674 1.000
H4 H -0.0537 10.0905 12.1073 1.000
H6 H -1.4955 12.2215 8.6687 1.000
O3 O 2.4547 14.2211 7.7110 1.000
N2 N 2.7022 15.0499 5.6751 1.000
H5 H -1.8061 11.3788 10.9923 1.000
C11 C 1.6548 15.3095 7.5443 1.000
N3 N 1.7654 15.8366 6.3655 1.000
C12 C 0.8426 15.7624 8.6684 1.000
C13 C 0.2756 17.0352 8.6164 1.000
C17 C 0.6489 14.9502 9.7892 1.000
H13 H 0.4057 17.6513 7.7352 1.000
C14 C -0.4529 17.5097 9.6913 1.000
C16 C -0.0918 15.4175 10.8607 1.000
H17 H 1.0779 13.9563 9.8191 1.000
H14 H -0.8872 18.5014 9.6619 1.000
C15 C -0.6232 16.7004 10.8125 1.000
H16 H -0.2556 14.7898 11.7279 1.000
S2 S -1.5661 17.3049 12.1872 1.000
O4 O -1.2464 16.4823 13.3171 1.000
O5 O -1.3360 18.7208 12.2602 1.000
N4 N -3.1369 17.0805 11.8433 1.000
C18 C -3.7438 17.9722 10.8223 1.000
C21 C -3.6880 15.7152 11.9674 1.000
H18A H -3.1901 18.9021 10.7854 1.000
H18B H -3.6720 17.5021 9.8493 1.000
C19 C -5.1985 18.2687 11.1277 1.000
H21A H -3.1102 15.1830 12.7130 1.000
H21B H -4.7024 15.7920 12.3390 1.000
C22 C -3.7091 14.8814 10.6984 1.000
H19A H -5.7549 17.3397 11.1154 1.000
H19B H -5.5980 18.9020 10.3452 1.000
C20 C -5.4112 18.9464 12.4593 1.000
H22A H -4.3645 15.3436 9.9705 1.000
H22B H -2.7130 14.8383 10.2756 1.000
C23 C -4.1974 13.4694 11.0026 1.000
H20A H -5.0419 18.3079 13.2522 1.000
H20B H -4.8755 19.8875 12.4749 1.000
H20C H -6.4683 19.1302 12.6063 1.000
H23A H -3.5368 13.0070 11.7255 1.000
H23B H -5.2010 13.5137 11.4073 1.000
H23C H -4.2009 12.8844 10.0912 1.000

[x, y, z]
H19B H -3.4398 7.6938 10.3452 1.000
C19 C -3.0403 7.0604 11.1277 1.000
C18 C -1.5856 6.7640 10.8223 1.000
H19A H -3.5967 6.1314 11.1154 1.000
C20 C -3.2530 7.7382 12.4593 1.000
H18A H -1.0319 7.6939 10.7854 1.000
H18B H -1.5138 6.2939 9.8493 1.000

N4 N -0.9788 5.8723 11.8433 1.000
H20A H -2.8837 7.0997 13.2522 1.000
H20B H -2.7173 8.6793 12.4749 1.000
H20C H -4.3102 7.9219 12.6063 1.000
C21 C -1.5298 4.5070 11.9674 1.000
S2 S 0.5920 6.0967 12.1872 1.000
C22 C -1.5509 3.6732 10.6984 1.000
H21A H -0.9521 3.9748 12.7130 1.000
H21B H -2.5442 4.5838 12.3390 1.000
C15 C 1.5350 5.4922 10.8125 1.000
O4 O 0.9117 5.2741 13.3171 1.000
O5 O 0.8221 7.5126 12.2602 1.000
H22A H -2.2063 4.1354 9.9705 1.000
H22B H -0.5549 3.6301 10.2756 1.000
C23 C -2.0393 2.2611 11.0026 1.000
C14 C 1.7053 6.3015 9.6913 1.000
C16 C 2.0663 4.2093 10.8607 1.000
H23A H -1.3786 1.7988 11.7255 1.000
H23B H -3.0428 2.3055 11.4073 1.000
H23C H -2.0427 1.6762 10.0912 1.000
C13 C 2.4338 5.8270 8.6164 1.000
H14 H 1.2709 7.2932 9.6619 1.000
C17 C 2.8070 3.7420 9.7892 1.000
H16 H 1.9026 3.5816 11.7279 1.000
C12 C 3.0007 4.5542 8.6684 1.000
H13 H 2.5639 6.4431 7.7352 1.000
H17 H 3.2360 2.7481 9.8191 1.000
C11 C 3.8130 4.1013 7.5443 1.000
N3 N 3.9235 4.6284 6.3655 1.000
O3 O 4.6128 3.0129 7.7110 1.000
N2 N 4.8603 3.8417 5.6751 1.000
C10 C 5.2335 2.9200 6.4926 1.000
S1 S 6.4275 1.6782 6.2423 1.000
C9 C 5.2842 0.2735 5.9686 1.000
H9B H 4.6791 0.4795 5.0945 1.000
N1 N 4.4152 0.0147 7.0854 1.000
H9A H 5.8660 -0.6167 5.7641 1.000
C8 C 3.1190 0.5355 7.1891 1.000
C1 C 4.8121 -0.6390 8.2643 1.000
O2 O 2.5573 1.1358 6.3023 1.000
C7 C 2.6442 0.1744 8.5462 1.000
C2 C 3.6480 -0.5431 9.1842 1.000
O1 O 5.8921 -1.1459 8.4328 1.000
C6 C 1.4421 0.4506 9.1674 1.000
C3 C 3.4838 -1.0287 10.4681 1.000
H6 H 0.6627 1.0132 8.6687 1.000
C5 C 1.2780 -0.0294 10.4674 1.000
C4 C 2.2719 -0.7551 11.1005 1.000
H3 H 4.2625 -1.5971 10.9614 1.000
H5 H 0.3521 0.1706 10.9923 1.000
H4 H 2.1045 -1.1177 12.1073 1.000

[-x, -y, -z]

H5 H -4.5372 9.0625 2.2366 1.000
C5 C -5.4631 9.2624 2.7615 1.000
C6 C -5.6272 8.7825 4.0616 1.000
C4 C -6.4570 9.9882 2.1284 1.000
H6 H -4.8478 8.2198 4.5603 1.000
C7 C -6.8293 9.0586 4.6828 1.000
C3 C -7.6689 10.2617 2.7609 1.000
H4 H -6.2896 10.3508 1.1217 1.000
C2 C -7.8331 9.7761 4.0448 1.000
C8 C -7.3041 8.6975 6.0398 1.000
H3 H -8.4476 10.8301 2.2676 1.000
C1 C -8.9972 9.8720 4.9647 1.000
O2 O -6.7423 8.0972 6.9267 1.000
N1 N -8.6003 9.2183 6.1435 1.000
O1 O -10.0772 10.3789 4.7962 1.000
C9 C -9.4693 8.9595 7.2603 1.000
S1 S -10.6126 7.5548 6.9866 1.000
H9A H -10.0511 9.8498 7.4648 1.000
H9B H -8.8641 8.7535 8.1345 1.000
C10 C -9.4186 6.3131 6.7363 1.000
O3 O -8.7979 6.2202 5.5179 1.000
N2 N -9.0454 5.3913 7.5539 1.000
C11 C -7.9981 5.1317 5.6846 1.000
N3 N -8.1086 4.6047 6.8634 1.000
C12 C -7.1858 4.6788 4.5605 1.000
C13 C -6.6189 3.4060 4.6125 1.000
C17 C -6.9921 5.4910 3.4398 1.000
C14 C -5.8903 2.9315 3.5377 1.000
H13 H -6.7490 2.7899 5.4937 1.000
C16 C -6.2514 5.0238 2.3682 1.000
H17 H -7.4211 6.4849 3.4099 1.000
C15 C -5.7200 3.7409 2.4164 1.000
H14 H -5.4560 1.9398 3.5671 1.000
H16 H -6.0877 5.6514 1.5011 1.000
S2 S -4.7771 3.1363 1.0418 1.000
N4 N -3.2063 3.3607 1.3856 1.000
O4 O -5.0968 3.9589 -0.0881 1.000
O5 O -5.0072 1.7204 0.9688 1.000
C18 C -2.5995 2.4691 2.4066 1.000
C21 C -2.6552 4.7260 1.2615 1.000
C19 C -1.1448 2.1726 2.1013 1.000
H18A H -3.1532 1.5391 2.4435 1.000
H18B H -2.6713 2.9391 3.3796 1.000
H21B H -1.6409 4.6492 0.8899 1.000
C22 C -2.6341 5.5598 2.5306 1.000
H21A H -3.2330 5.2582 0.5159 1.000
H19A H -0.5884 3.1016 2.1136 1.000
H19B H -0.7452 1.5393 2.8838 1.000
C20 C -0.9320 1.4948 0.7697 1.000
H22A H -1.9788 5.0976 3.2584 1.000
C23 C -2.1458 6.9719 2.2263 1.000
H22B H -3.6302 5.6030 2.9534 1.000
H20C H 0.1251 1.3111 0.6227 1.000

H20A H -1.3014 2.1334 -0.0233 1.000
H20B H -1.4678 0.5537 0.7541 1.000
H23C H -2.1423 7.5568 3.1378 1.000
H23B H -1.1423 6.9275 1.8216 1.000
H23A H -2.8065 7.4342 1.5035 1.000

[x, y, z]
S1 S -1.5372 1.6782 6.2423 1.000
C10 C -2.7312 2.9200 6.4926 1.000
C9 C -2.6805 0.2735 5.9686 1.000
O3 O -3.3519 3.0129 7.7110 1.000
N2 N -3.1044 3.8417 5.6751 1.000
N1 N -3.5495 0.0147 7.0854 1.000
H9A H -2.0987 -0.6167 5.7641 1.000
H9B H -3.2856 0.4795 5.0945 1.000
C11 C -4.1517 4.1013 7.5443 1.000
N3 N -4.0412 4.6284 6.3655 1.000
C1 C -3.1526 -0.6390 8.2643 1.000
C8 C -4.8457 0.5355 7.1891 1.000
C12 C -4.9640 4.5542 8.6684 1.000
O1 O -2.0726 -1.1459 8.4328 1.000
C2 C -4.3167 -0.5431 9.1842 1.000
O2 O -5.4074 1.1358 6.3023 1.000
C7 C -5.3205 0.1744 8.5462 1.000
C13 C -5.5309 5.8270 8.6164 1.000
C17 C -5.1577 3.7420 9.7892 1.000
C3 C -4.4809 -1.0287 10.4681 1.000
C6 C -6.5226 0.4506 9.1674 1.000
H13 H -5.4008 6.4431 7.7352 1.000
C14 C -6.2594 6.3015 9.6913 1.000
C16 C -5.8984 4.2093 10.8607 1.000
H17 H -4.7287 2.7481 9.8191 1.000
H3 H -3.7022 -1.5971 10.9614 1.000
C4 C -5.6928 -0.7551 11.1005 1.000
C5 C -6.6867 -0.0294 10.4674 1.000
H6 H -7.3020 1.0132 8.6687 1.000
H14 H -6.6938 7.2932 9.6619 1.000
C15 C -6.4297 5.4922 10.8125 1.000
H16 H -6.0621 3.5816 11.7279 1.000
H4 H -5.8602 -1.1177 12.1073 1.000
H5 H -7.6126 0.1706 10.9923 1.000
S2 S -7.3727 6.0967 12.1872 1.000
O4 O -7.0530 5.2741 13.3171 1.000
O5 O -7.1426 7.5126 12.2602 1.000
N4 N -8.9435 5.8723 11.8433 1.000
C18 C -9.5503 6.7640 10.8223 1.000
C21 C -9.4945 4.5070 11.9674 1.000
H18A H -8.9966 7.6939 10.7854 1.000
H18B H -9.4785 6.2939 9.8493 1.000
C19 C -11.0050 7.0604 11.1277 1.000
H21A H -8.9168 3.9748 12.7130 1.000
H21B H -10.5089 4.5838 12.3390 1.000
C22 C -9.5156 3.6732 10.6984 1.000

H19A H -11.5614 6.1314 11.1154 1.000
H19B H -11.4045 7.6938 10.3452 1.000
C20 C -11.2177 7.7382 12.4593 1.000
H22A H -10.1710 4.1354 9.9705 1.000
H22B H -8.5196 3.6301 10.2756 1.000
C23 C -10.0040 2.2611 11.0026 1.000
H20A H -10.8484 7.0997 13.2522 1.000
H20B H -10.6820 8.6793 12.4749 1.000
H20C H -12.2749 7.9219 12.6063 1.000
H23A H -9.3433 1.7988 11.7255 1.000
H23B H -11.0075 2.3055 11.4073 1.000
H23C H -10.0075 1.6762 10.0912 1.000

[x, y, z]
H20C H -2.2833 9.8971 -0.6227 1.000
C20 C -1.2261 9.7134 -0.7697 1.000
H20B H -0.6904 10.6545 -0.7540 1.000
H20A H -0.8568 9.0748 0.0233 1.000
C19 C -1.0134 9.0356 -2.1013 1.000
C18 C 0.4413 8.7391 -2.4066 1.000
H19A H -1.5698 8.1066 -2.1136 1.000
H19B H -1.4129 9.6689 -2.8838 1.000
N4 N 1.0481 7.8475 -1.3856 1.000
H18A H 0.9950 9.6691 -2.4435 1.000
H18B H 0.5131 8.2691 -3.3796 1.000
S2 S 2.6189 8.0719 -1.0418 1.000
C21 C 0.4971 6.4822 -1.2615 1.000
O5 O 2.8490 9.4878 -0.9688 1.000
O4 O 2.9387 7.2493 0.0881 1.000
C15 C 3.5619 7.4674 -2.4164 1.000
H21A H 1.0749 5.9500 -0.5159 1.000
H21B H -0.5173 6.5590 -0.8899 1.000
C22 C 0.4760 5.6484 -2.5306 1.000
C16 C 4.0933 6.1844 -2.3682 1.000
C14 C 3.7322 8.2767 -3.5377 1.000
H22B H 1.4721 5.6053 -2.9534 1.000
C23 C -0.0124 4.2363 -2.2263 1.000
H22A H -0.1794 6.1106 -3.2584 1.000
H16 H 3.9295 5.5568 -1.5011 1.000
C17 C 4.8339 5.7172 -3.4398 1.000
C13 C 4.4607 7.8022 -4.6125 1.000
H14 H 3.2978 9.2684 -3.5671 1.000
H23A H 0.6483 3.7740 -1.5035 1.000
H23B H -1.0159 4.2807 -1.8216 1.000
H23C H -0.0158 3.6514 -3.1378 1.000
C12 C 5.0277 6.5294 -4.5605 1.000
H17 H 5.2630 4.7233 -3.4099 1.000
H13 H 4.5908 8.4183 -5.4937 1.000
C11 C 5.8399 6.0765 -5.6846 1.000
O3 O 6.6398 4.9881 -5.5179 1.000
N3 N 5.9505 6.6035 -6.8634 1.000
C10 C 7.2605 4.8951 -6.7363 1.000
N2 N 6.8873 5.8169 -7.5539 1.000

S1 S 8.4544 3.6534 -6.9866 1.000
C9 C 7.3111 2.2487 -7.2603 1.000
N1 N 6.4421 1.9899 -6.1435 1.000
H9A H 7.8929 1.3585 -7.4648 1.000
H9B H 6.7060 2.4547 -8.1345 1.000
C1 C 6.8390 1.3362 -4.9647 1.000
C8 C 5.1459 2.5107 -6.0398 1.000
C2 C 5.6749 1.4321 -4.0448 1.000
O1 O 7.9190 0.8293 -4.7962 1.000
C7 C 4.6711 2.1496 -4.6828 1.000
O2 O 4.5842 3.1110 -6.9267 1.000
C3 C 5.5107 0.9465 -2.7609 1.000
C6 C 3.4690 2.4257 -4.0616 1.000
H3 H 6.2894 0.3781 -2.2676 1.000
C4 C 4.2988 1.2200 -2.1284 1.000
C5 C 3.3050 1.9458 -2.7615 1.000
H6 H 2.6896 2.9884 -4.5603 1.000
H4 H 4.1314 0.8575 -1.1217 1.000
H5 H 2.3790 2.1458 -2.2366 1.000

[x, y, z]
H20C H 7.8396 -1.3111 -0.6227 1.000
C20 C 8.8967 -1.4948 -0.7697 1.000
H20B H 9.4325 -0.5537 -0.7540 1.000
H20A H 9.2661 -2.1334 0.0233 1.000
C19 C 9.1095 -2.1726 -2.1013 1.000
C18 C 10.5642 -2.4691 -2.4066 1.000
H19A H 8.5531 -3.1016 -2.1136 1.000
H19B H 8.7099 -1.5393 -2.8838 1.000
N4 N 11.1710 -3.3607 -1.3856 1.000
H18A H 11.1179 -1.5391 -2.4435 1.000
H18B H 10.6360 -2.9391 -3.3796 1.000
S2 S 12.7418 -3.1363 -1.0418 1.000
C21 C 10.6199 -4.7260 -1.2615 1.000
O4 O 13.0615 -3.9589 0.0881 1.000
O5 O 12.9719 -1.7204 -0.9688 1.000
C15 C 13.6847 -3.7409 -2.4164 1.000
H21A H 11.1977 -5.2582 -0.5159 1.000
H21B H 9.6056 -4.6492 -0.8899 1.000
C22 C 10.5988 -5.5598 -2.5306 1.000
C14 C 13.8551 -2.9315 -3.5377 1.000
C16 C 14.2161 -5.0238 -2.3682 1.000
H22A H 9.9435 -5.0976 -3.2584 1.000
H22B H 11.5949 -5.6030 -2.9534 1.000
C23 C 10.1105 -6.9719 -2.2263 1.000
C13 C 14.5836 -3.4060 -4.6125 1.000
H14 H 13.4207 -1.9398 -3.5671 1.000
H16 H 14.0524 -5.6514 -1.5011 1.000
C17 C 14.9568 -5.4910 -3.4398 1.000
H23A H 10.7712 -7.4342 -1.5035 1.000
H23B H 9.1070 -6.9275 -1.8216 1.000
H23C H 10.1070 -7.5568 -3.1378 1.000
C12 C 15.1505 -4.6788 -4.5605 1.000

H13 H 14.7137 -2.7899 -5.4937 1.000
H17 H 15.3858 -6.4849 -3.4099 1.000
C11 C 15.9628 -5.1317 -5.6846 1.000
O3 O 16.7626 -6.2202 -5.5179 1.000
N3 N 16.0733 -4.6047 -6.8634 1.000
C10 C 17.3833 -6.3131 -6.7363 1.000
N2 N 17.0101 -5.3913 -7.5539 1.000
S1 S 18.5773 -7.5548 -6.9866 1.000
C9 C 17.4340 -8.9595 -7.2603 1.000
N1 N 16.5650 -9.2183 -6.1435 1.000
H9A H 18.0158 -9.8498 -7.4648 1.000
H9B H 16.8288 -8.7535 -8.1345 1.000
C1 C 16.9619 -9.8720 -4.9647 1.000
C8 C 15.2688 -8.6975 -6.0398 1.000
O1 O 18.0419 -10.3789 -4.7962 1.000
C2 C 15.7978 -9.7761 -4.0448 1.000
O2 O 14.7070 -8.0972 -6.9267 1.000
C7 C 14.7940 -9.0586 -4.6828 1.000
C3 C 15.6336 -10.2617 -2.7609 1.000
C6 C 13.5919 -8.7825 -4.0616 1.000
H3 H 16.4123 -10.8301 -2.2676 1.000
C4 C 14.4217 -9.9882 -2.1284 1.000
C5 C 13.4278 -9.2624 -2.7615 1.000
H6 H 12.8125 -8.2198 -4.5603 1.000
H4 H 14.2543 -10.3508 -1.1217 1.000
H5 H 12.5019 -9.0625 -2.2366 1.000

[x, y, z]
O5 O 5.0072 -1.7204 -0.9688 1.000
S2 S 4.7771 -3.1363 -1.0418 1.000
O4 O 5.0968 -3.9589 0.0881 1.000
N4 N 3.2063 -3.3607 -1.3856 1.000
C15 C 5.7200 -3.7409 -2.4164 1.000
C18 C 2.5995 -2.4691 -2.4066 1.000
C21 C 2.6552 -4.7260 -1.2615 1.000
C14 C 5.8903 -2.9315 -3.5377 1.000
C16 C 6.2514 -5.0238 -2.3682 1.000
C19 C 1.1448 -2.1726 -2.1013 1.000
H18A H 3.1532 -1.5391 -2.4435 1.000
H18B H 2.6713 -2.9391 -3.3796 1.000
H21A H 3.2330 -5.2582 -0.5159 1.000
H21B H 1.6409 -4.6492 -0.8899 1.000
C22 C 2.6341 -5.5598 -2.5306 1.000
C13 C 6.6189 -3.4060 -4.6125 1.000
H14 H 5.4560 -1.9398 -3.5671 1.000
H16 H 6.0877 -5.6514 -1.5011 1.000
C17 C 6.9921 -5.4910 -3.4398 1.000
C20 C 0.9320 -1.4948 -0.7697 1.000
H19A H 0.5884 -3.1016 -2.1136 1.000
H19B H 0.7452 -1.5393 -2.8838 1.000
H22A H 1.9788 -5.0976 -3.2584 1.000
H22B H 3.6302 -5.6030 -2.9534 1.000
C23 C 2.1458 -6.9719 -2.2263 1.000

C12 C 7.1858 -4.6788 -4.5605 1.000
H13 H 6.7490 -2.7899 -5.4937 1.000
H17 H 7.4211 -6.4849 -3.4099 1.000
H20B H 1.4678 -0.5537 -0.7540 1.000
H20A H 1.3014 -2.1334 0.0233 1.000
H20C H -0.1251 -1.3111 -0.6227 1.000
H23A H 2.8065 -7.4342 -1.5035 1.000
H23B H 1.1423 -6.9275 -1.8216 1.000
H23C H 2.1423 -7.5568 -3.1378 1.000
C11 C 7.9981 -5.1317 -5.6846 1.000
O3 O 8.7979 -6.2202 -5.5179 1.000
N3 N 8.1086 -4.6047 -6.8634 1.000
C10 C 9.4186 -6.3131 -6.7363 1.000
N2 N 9.0454 -5.3913 -7.5539 1.000
S1 S 10.6126 -7.5548 -6.9866 1.000
C9 C 9.4693 -8.9595 -7.2603 1.000
N1 N 8.6003 -9.2183 -6.1435 1.000
H9A H 10.0511 -9.8498 -7.4648 1.000
H9B H 8.8641 -8.7535 -8.1345 1.000
C1 C 8.9972 -9.8720 -4.9647 1.000
C8 C 7.3041 -8.6975 -6.0398 1.000
O1 O 10.0772 -10.3789 -4.7962 1.000
C2 C 7.8331 -9.7761 -4.0448 1.000
O2 O 6.7423 -8.0972 -6.9267 1.000
C7 C 6.8293 -9.0586 -4.6828 1.000
C3 C 7.6689 -10.2617 -2.7609 1.000
C6 C 5.6272 -8.7825 -4.0616 1.000
H3 H 8.4476 -10.8301 -2.2676 1.000
C4 C 6.4570 -9.9882 -2.1284 1.000
C5 C 5.4631 -9.2624 -2.7615 1.000
H6 H 4.8478 -8.2198 -4.5603 1.000
H4 H 6.2896 -10.3508 -1.1217 1.000
H5 H 4.5372 -9.0625 -2.2366 1.000

[-x, -y, -z]
C20 C -3.0902 12.7030 0.7697 1.000
H20B H -3.6259 11.7620 0.7541 1.000
H20C H -2.0331 12.5193 0.6227 1.000
C19 C -3.3029 13.3808 2.1013 1.000
H20A H -3.4595 13.3416 -0.0233 1.000
H19B H -2.9034 12.7475 2.8838 1.000
C18 C -4.7576 13.6773 2.4066 1.000
H19A H -2.7465 14.3098 2.1136 1.000
N4 N -5.3645 14.5689 1.3856 1.000
H18A H -5.3113 12.7473 2.4435 1.000
H18B H -4.8294 14.1473 3.3796 1.000
S2 S -6.9353 14.3445 1.0418 1.000
C21 C -4.8134 15.9342 1.2615 1.000
O4 O -7.2550 15.1671 -0.0881 1.000
O5 O -7.1654 12.9286 0.9688 1.000
C15 C -7.8782 14.9491 2.4164 1.000
H21A H -5.3912 16.4664 0.5159 1.000
H21B H -3.7990 15.8574 0.8899 1.000

C22 C -4.7923 16.7680 2.5306 1.000
C14 C -8.0485 14.1398 3.5377 1.000
C16 C -8.4096 16.2320 2.3682 1.000
H22A H -4.1369 16.3059 3.2584 1.000
H22B H -5.7884 16.8112 2.9534 1.000
C23 C -4.3040 18.1801 2.2263 1.000
C13 C -8.7770 14.6142 4.6125 1.000
H14 H -7.6142 13.1480 3.5671 1.000
H16 H -8.2458 16.8597 1.5011 1.000
C17 C -9.1503 16.6992 3.4398 1.000
H23A H -4.9646 18.6424 1.5035 1.000
H23B H -3.3004 18.1357 1.8216 1.000
H23C H -4.3005 18.7650 3.1378 1.000
C12 C -9.3440 15.8871 4.5605 1.000
H13 H -8.9071 13.9981 5.4937 1.000
H17 H -9.5793 17.6931 3.4099 1.000
C11 C -10.1562 16.3399 5.6846 1.000
O3 O -10.9561 17.4284 5.5179 1.000
N3 N -10.2668 15.8129 6.8634 1.000
C10 C -11.5768 17.5213 6.7363 1.000
N2 N -11.2036 16.5995 7.5539 1.000
S1 S -12.7708 18.7630 6.9866 1.000
C9 C -11.6275 20.1678 7.2603 1.000
N1 N -10.7584 20.4265 6.1435 1.000
H9A H -12.2092 21.0580 7.4648 1.000
H9B H -11.0223 19.9617 8.1345 1.000
C1 C -11.1553 21.0802 4.9647 1.000
C8 C -9.4622 19.9057 6.0398 1.000
O1 O -12.2354 21.5871 4.7962 1.000
C2 C -9.9912 20.9843 4.0448 1.000
O2 O -8.9005 19.3054 6.9267 1.000
C7 C -8.9874 20.2668 4.6828 1.000
C3 C -9.8271 21.4699 2.7609 1.000
C6 C -7.7853 19.9907 4.0616 1.000
H3 H -10.6058 22.0383 2.2676 1.000
C4 C -8.6151 21.1964 2.1284 1.000
C5 C -7.6213 20.4706 2.7615 1.000
H6 H -7.0059 19.4280 4.5603 1.000
H4 H -8.4477 21.5590 1.1217 1.000
H5 H -6.6953 20.2707 2.2366 1.000

[x, y, z]
S1 S -3.6954 12.8865 6.2423 1.000
C9 C -4.8386 11.4817 5.9686 1.000
C10 C -4.8893 14.1282 6.4926 1.000
H9A H -4.2569 10.5915 5.7641 1.000
H9B H -5.4438 11.6878 5.0945 1.000
N1 N -5.7077 11.2229 7.0854 1.000
O3 O -5.5100 14.2211 7.7110 1.000
N2 N -5.2625 15.0499 5.6751 1.000
C1 C -5.3108 10.5692 8.2643 1.000
C8 C -7.0039 11.7438 7.1891 1.000
C11 C -6.3099 15.3095 7.5443 1.000

N3 N -6.1993 15.8366 6.3655 1.000
O1 O -4.2307 10.0623 8.4328 1.000
C2 C -6.4749 10.6651 9.1842 1.000
C7 C -7.4787 11.3827 8.5462 1.000
O2 O -7.5656 12.3440 6.3023 1.000
C12 C -7.1221 15.7624 8.6684 1.000
C3 C -6.6391 10.1795 10.4681 1.000
C6 C -8.6808 11.6588 9.1674 1.000
C13 C -7.6891 17.0352 8.6164 1.000
C17 C -7.3158 14.9502 9.7892 1.000
H3 H -5.8603 9.6112 10.9614 1.000
C4 C -7.8510 10.4531 11.1005 1.000
C5 C -8.8448 11.1789 10.4674 1.000
H6 H -9.4602 12.2215 8.6687 1.000
H13 H -7.5590 17.6513 7.7352 1.000
C14 C -8.4176 17.5097 9.6913 1.000
C16 C -8.0565 15.4175 10.8607 1.000
H17 H -6.8868 13.9563 9.8191 1.000
H4 H -8.0184 10.0905 12.1073 1.000
H5 H -9.7708 11.3788 10.9923 1.000
H14 H -8.8519 18.5014 9.6619 1.000
C15 C -8.5879 16.7004 10.8125 1.000
H16 H -8.2203 14.7898 11.7279 1.000
S2 S -9.5308 17.3049 12.1872 1.000
O4 O -9.2111 16.4823 13.3171 1.000
O5 O -9.3007 18.7208 12.2602 1.000
N4 N -11.1016 17.0805 11.8433 1.000
C18 C -11.7085 17.9722 10.8223 1.000
C21 C -11.6527 15.7152 11.9674 1.000
H18A H -11.1548 18.9021 10.7854 1.000
H18B H -11.6367 17.5021 9.8493 1.000
C19 C -13.1632 18.2687 11.1277 1.000
H21A H -11.0749 15.1830 12.7130 1.000
H21B H -12.6671 15.7920 12.3390 1.000
C22 C -11.6738 14.8814 10.6984 1.000
H19A H -13.7196 17.3397 11.1154 1.000
H19B H -13.5627 18.9020 10.3452 1.000
C20 C -13.3759 18.9464 12.4593 1.000
H22A H -12.3292 15.3436 9.9705 1.000
H22B H -10.6777 14.8383 10.2756 1.000
C23 C -12.1621 13.4694 11.0026 1.000
H20A H -13.0066 18.3079 13.2522 1.000
H20B H -12.8402 19.8875 12.4749 1.000
H20C H -14.4331 19.1302 12.6063 1.000
H23A H -11.5015 13.0070 11.7255 1.000
H23B H -13.1657 13.5137 11.4073 1.000
H23C H -12.1656 12.8844 10.0912 1.000

Selected Atoms
58 atoms, fractional coordinates
Label Symbol x y z Occ.

O4 O 0.4538 0.3520 -0.0067 1.000
H20A H 0.8877 0.1900 -0.0018 1.000
S1 S 0.0098 0.7671 0.5281 1.000
S2 S 0.4998 0.2937 0.0787 1.000
O1 O 0.0953 0.9899 0.3625 1.000
O2 O 0.5075 0.8147 0.5236 1.000
O3 O 0.1718 0.6285 0.4171 1.000
O5 O 0.4351 0.1664 0.0732 1.000
N1 N 0.2834 0.9043 0.4644 1.000
N2 N 0.1672 0.5816 0.5710 1.000
N3 N 0.2501 0.5023 0.5188 1.000
N4 N 0.7103 0.3183 0.1047 1.000
C1 C 0.2225 0.9469 0.3753 1.000
C2 C 0.3453 0.9261 0.3058 1.000
C3 C 0.3483 0.9523 0.2087 1.000
H3 H 0.2530 0.9965 0.1714 1.000
C4 C 0.4794 0.9195 0.1609 1.000
H4 H 0.4862 0.9384 0.0848 1.000
C5 C 0.6011 0.8632 0.2087 1.000
H5 H 0.7005 0.8383 0.1691 1.000
C6 C 0.5986 0.8377 0.3070 1.000
H6 H 0.6942 0.7941 0.3447 1.000
C7 C 0.4685 0.8706 0.3540 1.000
C8 C 0.4312 0.8565 0.4566 1.000
C9 C 0.1936 0.8961 0.5488 1.000
H9A H 0.1467 0.9782 0.5643 1.000
H9B H 0.2845 0.8893 0.6149 1.000
C10 C 0.1240 0.6530 0.5092 1.000
C11 C 0.2498 0.5336 0.4297 1.000
C12 C 0.3151 0.4782 0.3447 1.000
C13 C 0.3567 0.3653 0.3487 1.000
H13 H 0.3456 0.3221 0.4153 1.000
C14 C 0.4121 0.3087 0.2674 1.000
H14 H 0.4434 0.2206 0.2696 1.000
C15 C 0.4275 0.3659 0.1827 1.000
C16 C 0.3907 0.4798 0.1790 1.000
H16 H 0.4066 0.5242 0.1135 1.000
C17 C 0.3334 0.5357 0.2600 1.000
H17 H 0.3029 0.6240 0.2578 1.000
C18 C 0.7883 0.2524 0.1819 1.000
H18A H 0.6971 0.1699 0.1847 1.000
H18B H 0.8129 0.3072 0.2555 1.000
C19 C 0.9568 0.2218 0.1588 1.000
C20 C 0.9367 0.1436 0.0582 1.000
H20B H 0.8463 0.0595 0.0570 1.000
C21 C 0.8097 0.4385 0.0954 1.000
H21A H 0.7330 0.4760 0.0390 1.000
H21B H 0.9267 0.4267 0.0673 1.000
C22 C 0.8615 0.5298 0.1913 1.000
H22A H 0.9492 0.4982 0.2463 1.000
H22B H 0.7471 0.5392 0.2233 1.000
C23 C 0.9500 0.6517 0.1683 1.000
H23A H 0.8617 0.6833 0.1137 1.000

H23C H 0.9854 0.7160 0.2372 1.000
H19A H 1.0494 0.3049 0.1598 1.000
H19B H 1.0095 0.1758 0.2180 1.000
H20C H 1.0616 0.1253 0.0471 1.000
H23B H 1.0657 0.6423 0.1377 1.000

Unselected Atoms
870 atoms, fractional coordinates
Label Symbol x y z Occ.

H20C H -0.0616 0.8747 -0.0471 1.000
S1 S -0.0098 0.2329 0.4719 1.000
O3 O -0.1718 0.3715 0.5829 1.000
N2 N -0.1672 0.4184 0.4290 1.000
N3 N -0.2501 0.4977 0.4812 1.000
H5 H -0.2995 0.8383 0.1691 1.000
H6 H -0.3058 0.7941 0.3447 1.000
C10 C -0.1240 0.3470 0.4908 1.000
C11 C -0.2498 0.4664 0.5703 1.000
C13 C -0.3567 0.6347 0.6513 1.000
H13 H -0.3456 0.6779 0.5847 1.000
C19 C -0.0432 0.2218 0.1588 1.000
H19B H -0.0095 0.8242 0.7820 1.000
H21B H -0.0733 0.4267 0.0673 1.000
C22 C -0.1385 0.5298 0.1913 1.000
H22A H -0.0508 0.4982 0.2463 1.000
C23 C -0.0500 0.6517 0.1683 1.000
H23C H -0.0146 0.7160 0.2372 1.000
S1 S -0.0098 1.2329 0.4719 1.000
O1 O -0.0953 1.0101 0.6374 1.000
C1 C -0.2225 1.0531 0.6247 1.000
C9 C -0.1936 1.1039 0.4512 1.000
H9A H -0.1467 1.0218 0.4357 1.000
H9B H -0.2845 1.1107 0.3851 1.000
C20 C -0.0633 1.1436 0.0582 1.000
H20B H -0.1537 1.0595 0.0570 1.000
O5 O 0.5649 -0.1664 -0.0732 1.000
H20B H 0.1537 -0.0594 -0.0570 1.000
H20C H 0.9384 -0.1253 -0.0471 1.000
C2 C 0.3453 -0.0739 0.3058 1.000
C3 C 0.3483 -0.0477 0.2087 1.000
H3 H 0.2530 -0.0035 0.1714 1.000
C4 C 0.4794 -0.0805 0.1609 1.000
H4 H 0.4862 -0.0616 0.0848 1.000
C5 C 0.6011 -0.1368 0.2087 1.000
H5 H 0.7005 -0.1617 0.1691 1.000
S2 S 0.5002 0.7063 -0.0788 1.000
O5 O 0.5649 0.8336 -0.0732 1.000
N4 N 0.2897 0.6817 -0.1047 1.000
C3 C 0.6517 0.0477 -0.2087 1.000
H3 H 0.7470 0.0035 -0.1714 1.000
C4 C 0.5206 0.0805 -0.1609 1.000

H4 H 0.5138 0.0616 -0.0848 1.000
C5 C 0.3989 0.1368 -0.2088 1.000
H5 H 0.2995 0.1617 -0.1691 1.000
C16 C 0.6093 0.5202 -0.1790 1.000
H16 H 0.5934 0.4758 -0.1135 1.000
H18A H 0.3029 0.8301 -0.1847 1.000
C20 C 0.0633 0.8564 -0.0582 1.000
H20B H 0.1537 0.9406 -0.0570 1.000
H20C H 0.9384 0.8747 -0.0471 1.000
C21 C 0.1903 0.5615 -0.0954 1.000
H21A H 0.2670 0.5240 -0.0390 1.000
H21B H 0.0733 0.5733 -0.0673 1.000
H22B H 0.2529 0.4608 -0.2232 1.000
C23 C 0.0500 0.3483 -0.1683 1.000
H23A H 0.1383 0.3167 -0.1137 1.000
H23B H 0.9343 0.3577 -0.1377 1.000
S1 S 0.9902 0.2329 0.4719 1.000
O2 O 0.4925 0.1853 0.4764 1.000
O4 O 0.5462 0.6480 0.0067 1.000
N2 N 0.8328 0.4184 0.4290 1.000
N3 N 0.7499 0.4977 0.4812 1.000
H6 H 0.3058 0.2059 0.6553 1.000
C8 C 0.5688 0.1435 0.5434 1.000
C9 C 0.8064 0.1039 0.4512 1.000
H9B H 0.7155 0.1107 0.3851 1.000
C10 C 0.8760 0.3470 0.4908 1.000
C11 C 0.7502 0.4664 0.5703 1.000
C12 C 0.6849 0.5218 0.6553 1.000
C13 C 0.6433 0.6347 0.6513 1.000
H13 H 0.6544 0.6779 0.5847 1.000
C14 C 0.5879 0.6913 0.7326 1.000
H14 H 0.5566 0.7794 0.7304 1.000
C18 C 0.2117 0.7477 0.8181 1.000
H18A H 0.3029 0.8301 0.8153 1.000
H18B H 0.1871 0.6927 0.7445 1.000
H19A H 0.0494 0.3049 0.1598 1.000
H19B H 0.0095 0.1758 0.2180 1.000
H20A H 0.1124 0.8100 0.0018 1.000
H20C H 0.0616 0.1253 0.0471 1.000
C22 C 0.1385 0.4702 0.8087 1.000
H22A H 0.0508 0.5018 0.7537 1.000
H22B H 0.2529 0.4608 0.7768 1.000
H23B H 0.0657 0.6423 0.1377 1.000
H4 H 0.5138 1.0616 -0.0848 1.000
O1 O 0.9047 1.0101 0.6374 1.000
O2 O 0.4925 1.1853 0.4764 1.000
O5 O 0.4351 1.1664 0.0732 1.000
N1 N 0.7166 1.0957 0.5356 1.000
C1 C 0.7775 1.0531 0.6247 1.000
C2 C 0.6547 1.0739 0.6942 1.000
C3 C 0.6517 1.0477 0.7913 1.000
C4 C 0.5206 1.0805 0.8391 1.000
C5 C 0.3989 1.1368 0.7912 1.000

C6 C 0.4014 1.1623 0.6930 1.000
H6 H 0.3058 1.2059 0.6553 1.000
C7 C 0.5315 1.1294 0.6460 1.000
C8 C 0.5688 1.1436 0.5434 1.000
C9 C 0.8064 1.1039 0.4512 1.000
H9A H 0.8533 1.0218 0.4357 1.000
H9B H 0.7155 1.1107 0.3851 1.000
C14 C 0.4121 1.3087 0.2674 1.000
H14 H 0.4434 1.2206 0.2696 1.000
H18A H 0.6971 1.1699 0.1847 1.000
H19B H 0.0095 1.1758 0.2180 1.000
H20B H 0.8463 1.0595 0.0570 1.000
H20C H 0.0616 1.1253 0.0471 1.000
C20 C 1.0633 -0.1436 -0.0582 1.000
H20B H 1.1537 -0.0594 -0.0570 1.000
O1 O 1.0953 -0.0101 0.3625 1.000
H3 H 1.2530 -0.0035 0.1714 1.000
H20A H 1.1123 -0.1900 0.0018 1.000
H5 H 1.2995 0.1617 -0.1691 1.000
C20 C 1.0633 0.8564 -0.0582 1.000
C21 C 1.1903 0.5615 -0.0954 1.000
H21A H 1.2670 0.5240 -0.0390 1.000
H21B H 1.0733 0.5733 -0.0673 1.000
C23 C 1.0500 0.3483 -0.1683 1.000
H23A H 1.1383 0.3167 -0.1137 1.000
H23C H 1.0146 0.2840 -0.2372 1.000
S1 S 1.0098 0.7671 0.5281 1.000
S2 S 1.4998 0.2937 0.0787 1.000
O1 O 1.0953 0.9899 0.3625 1.000
O3 O 1.1718 0.6285 0.4171 1.000
O5 O 1.4351 0.1664 0.0732 1.000
N1 N 1.2834 0.9043 0.4644 1.000
C1 C 1.2225 0.9469 0.3753 1.000
C2 C 1.3453 0.9261 0.3058 1.000
C3 C 1.3483 0.9523 0.2087 1.000
H3 H 1.2530 0.9965 0.1714 1.000
C10 C 1.1240 0.6530 0.5092 1.000
C11 C 1.2498 0.5336 0.4297 1.000
C12 C 1.3151 0.4782 0.3447 1.000
C13 C 1.3567 0.3653 0.3487 1.000
H13 H 1.3456 0.3221 0.4153 1.000
C14 C 1.4121 0.3087 0.2674 1.000
H14 H 1.4434 0.2206 0.2696 1.000
C15 C 1.4275 0.3659 0.1827 1.000
C16 C 1.3907 0.4798 0.1790 1.000
H16 H 1.4066 0.5242 0.1135 1.000
C17 C 1.3334 0.5357 0.2600 1.000
H17 H 1.3029 0.6240 0.2578 1.000
H20A H 1.1123 0.8100 0.0018 1.000
C9 C -0.1936 0.1039 0.4512 1.000
C5 C -0.3989 0.8632 0.2087 1.000
C6 C -0.4014 0.8377 0.3070 1.000
C12 C -0.3151 0.5218 0.6553 1.000

C14 C -0.4121 0.6913 0.7326 1.000
C18 C -0.2117 0.2524 0.1819 1.000
C20 C -0.0633 0.1436 0.0582 1.000
C19 C 0.0432 0.7782 0.8412 1.000
C21 C -0.1903 0.4385 0.0954 1.000
H22B H -0.2529 0.5392 0.2233 1.000
H23A H -0.1383 0.6833 0.1137 1.000
C10 C -0.1240 1.3470 0.4908 1.000
N1 N -0.2834 1.0957 0.5356 1.000
C2 C -0.3453 1.0739 0.6942 1.000
C19 C -0.0432 1.2218 0.1588 1.000
H20A H -0.1123 1.1900 -0.0018 1.000
S2 S 0.5002 -0.2937 -0.0788 1.000
C20 C 0.0633 -0.1436 -0.0582 1.000
C1 C 0.2225 -0.0531 0.3753 1.000
C7 C 0.4685 -0.1294 0.3540 1.000
C6 C 0.5986 -0.1623 0.3070 1.000
C15 C 0.5725 0.6341 -0.1827 1.000
C18 C 0.2117 0.7477 -0.1819 1.000
C2 C 0.6547 0.0739 -0.3058 1.000
C6 C 0.4014 0.1623 -0.3070 1.000
C17 C 0.6665 0.4643 -0.2600 1.000
C19 C 0.0432 0.7782 -0.1588 1.000
C22 C 0.1385 0.4702 -0.1913 1.000
H23B H -0.0657 0.3577 -0.1377 1.000
H23C H 0.0146 0.2840 -0.2372 1.000
C6 C 0.4014 0.1623 0.6930 1.000
N1 N 0.7166 0.0957 0.5356 1.000
C7 C 0.5315 0.1294 0.6460 1.000
H9A H 0.8533 0.0218 0.4357 1.000
O3 O 0.8282 0.3715 0.5829 1.000
C17 C 0.6665 0.4643 0.7400 1.000
C15 C 0.5725 0.6341 0.8173 1.000
N4 N 0.2897 0.6817 0.8953 1.000
C21 C 0.1903 0.5615 0.9046 1.000
C23 C 0.0500 0.3483 0.8317 1.000
C4 C 0.5206 1.0805 -0.1609 1.000
S2 S 0.4998 1.2937 0.0787 1.000
H3 H 0.7470 1.0035 0.8286 1.000
H4 H 0.5138 1.0616 0.9152 1.000
H5 H 0.2995 1.1616 0.8309 1.000
S1 S 0.9902 1.2329 0.4719 1.000
C13 C 0.3567 1.3653 0.3487 1.000
C15 C 0.4275 1.3659 0.1827 1.000
C18 C 0.7883 1.2523 0.1819 1.000
C20 C 0.9367 1.1436 0.0582 1.000
C19 C 1.0432 -0.2218 -0.1588 1.000
C1 C 1.2225 -0.0531 0.3753 1.000
C3 C 1.3483 -0.0477 0.2087 1.000
C5 C 1.3989 0.1368 -0.2088 1.000
C19 C 1.0432 0.7782 -0.1588 1.000
H20B H 1.1537 0.9406 -0.0570 1.000
N4 N 1.2897 0.6817 -0.1047 1.000

C22 C 1.1385 0.4702 -0.1913 1.000
C9 C 1.1936 0.8961 0.5488 1.000
O4 O 1.4538 0.3520 -0.0067 1.000
N4 N 1.7103 0.3183 0.1047 1.000
C8 C 1.4312 0.8565 0.4566 1.000
C7 C 1.4685 0.8706 0.3540 1.000
C4 C 1.4794 0.9195 0.1609 1.000
N2 N 1.1672 0.5816 0.5710 1.000
N3 N 1.2501 0.5023 0.5188 1.000
N1 N -0.2834 0.0957 0.5356 1.000
H9A H -0.1467 0.0218 0.4357 1.000
H9B H -0.2845 0.1107 0.3851 1.000
C4 C -0.5206 0.9195 0.1609 1.000
C7 C -0.5315 0.8706 0.3540 1.000
C17 C -0.3335 0.4643 0.7400 1.000
H14 H -0.4434 0.7794 0.7304 1.000
C15 C -0.4275 0.6341 0.8173 1.000
N4 N -0.2897 0.3183 0.1047 1.000
H18A H -0.3029 0.1699 0.1847 1.000
H18B H -0.1871 0.3072 0.2555 1.000
H20A H -0.1123 0.1900 -0.0018 1.000
H20B H -0.1537 0.0595 0.0570 1.000
H19A H -0.0494 0.6951 0.8402 1.000
C20 C 0.0633 0.8564 0.9418 1.000
H21A H -0.2670 0.4760 0.0390 1.000
O3 O -0.1718 1.3715 0.5829 1.000
N2 N -0.1672 1.4184 0.4290 1.000
C8 C -0.4312 1.1436 0.5434 1.000
C3 C -0.3483 1.0477 0.7913 1.000
C7 C -0.4685 1.1294 0.6460 1.000
C18 C -0.2117 1.2523 0.1819 1.000
H19A H 0.0494 1.3049 0.1598 1.000
O4 O 0.5462 -0.3520 0.0067 1.000
N4 N 0.2897 -0.3183 -0.1047 1.000
C15 C 0.5725 -0.3659 -0.1827 1.000
C19 C 0.0432 -0.2218 -0.1588 1.000
H20A H 0.1124 -0.1900 0.0018 1.000
H20C H -0.0616 -0.1253 -0.0471 1.000
O1 O 0.0953 -0.0101 0.3625 1.000
N1 N 0.2834 -0.0957 0.4644 1.000
C8 C 0.4312 -0.1435 0.4566 1.000
H6 H 0.6942 -0.2059 0.3447 1.000
C14 C 0.5879 0.6913 -0.2674 1.000
H18B H 0.1871 0.6927 -0.2555 1.000
C1 C 0.7775 0.0531 -0.3753 1.000
C7 C 0.5315 0.1294 -0.3540 1.000
H6 H 0.3058 0.2059 -0.3447 1.000
C12 C 0.6849 0.5218 -0.3447 1.000
H17 H 0.6971 0.3760 -0.2578 1.000
H19A H -0.0494 0.6951 -0.1598 1.000
H19B H -0.0095 0.8242 -0.2180 1.000
H22A H 0.0508 0.5018 -0.2463 1.000
C5 C 0.3989 0.1368 0.7912 1.000

C1 C 0.7775 0.0531 0.6247 1.000
C2 C 0.6547 0.0739 0.6942 1.000
C16 C 0.6093 0.5202 0.8210 1.000
H17 H 0.6971 0.3760 0.7422 1.000
S2 S 0.5002 0.7063 0.9212 1.000
H21A H 0.2670 0.5240 0.9610 1.000
H21B H 0.0733 0.5733 0.9327 1.000
H23A H 0.1383 0.3167 0.8863 1.000
H23B H -0.0657 0.3577 0.8623 1.000
H23C H 0.0146 0.2840 0.7628 1.000
C3 C 0.6517 1.0477 -0.2087 1.000
C5 C 0.3989 1.1368 -0.2088 1.000
O4 O 0.4538 1.3520 -0.0067 1.000
N4 N 0.7103 1.3183 0.1047 1.000
C10 C 0.8760 1.3470 0.4908 1.000
C12 C 0.3151 1.4782 0.3447 1.000
H13 H 0.3456 1.3221 0.4153 1.000
C16 C 0.3907 1.4798 0.1790 1.000
H18B H 0.8129 1.3073 0.2555 1.000
C19 C 0.9568 1.2218 0.1588 1.000
H20A H 0.8877 1.1900 -0.0018 1.000
H20C H 1.0616 1.1253 0.0471 1.000
C18 C 1.2117 -0.2523 -0.1819 1.000
H19A H 0.9506 -0.3049 -0.1598 1.000
H19B H 0.9905 -0.1758 -0.2180 1.000
N1 N 1.2834 -0.0957 0.4644 1.000
C2 C 1.3453 -0.0739 0.3058 1.000
C4 C 1.4794 -0.0805 0.1609 1.000
C4 C 1.5206 0.0805 -0.1609 1.000
C6 C 1.4014 0.1623 -0.3070 1.000
C18 C 1.2117 0.7477 -0.1819 1.000
H19A H 0.9506 0.6951 -0.1598 1.000
H19B H 0.9905 0.8242 -0.2180 1.000
S2 S 1.5002 0.7063 -0.0788 1.000
H22A H 1.0508 0.5018 -0.2463 1.000
H22B H 1.2529 0.4608 -0.2232 1.000
H9A H 1.1467 0.9782 0.5643 1.000
H9B H 1.2845 0.8893 0.6149 1.000
C18 C 1.7883 0.2524 0.1819 1.000
C21 C 1.8097 0.4385 0.0954 1.000
O2 O 1.5075 0.8147 0.5236 1.000
C6 C 1.5986 0.8377 0.3070 1.000
H4 H 1.4862 0.9384 0.0848 1.000
C5 C 1.6011 0.8632 0.2087 1.000
C1 C -0.2225 0.0531 0.6247 1.000
C8 C -0.4312 0.1435 0.5434 1.000
C3 C -0.6517 0.9523 0.2087 1.000
H4 H -0.5138 0.9384 0.0848 1.000
C2 C -0.6547 0.9261 0.3058 1.000
C8 C -0.5688 0.8565 0.4566 1.000
C16 C -0.3907 0.5202 0.8210 1.000
H17 H -0.3029 0.3760 0.7422 1.000
S2 S -0.4998 0.7063 0.9212 1.000

S2 S -0.5002 0.2937 0.0787 1.000
H20A H 0.1124 0.8100 1.0018 1.000
H20B H 0.1537 0.9406 0.9430 1.000
H20C H -0.0616 0.8747 0.9529 1.000
C11 C -0.2498 1.4664 0.5703 1.000
N3 N -0.2501 1.4977 0.4812 1.000
O2 O -0.5075 1.1853 0.4764 1.000
H3 H -0.2530 1.0035 0.8286 1.000
C4 C -0.4794 1.0805 0.8391 1.000
C6 C -0.5986 1.1623 0.6930 1.000
N4 N -0.2897 1.3183 0.1047 1.000
H18A H -0.3029 1.1699 0.1847 1.000
H18B H -0.1871 1.3073 0.2555 1.000
C18 C 0.2117 -0.2523 -0.1819 1.000
C21 C 0.1903 -0.4385 -0.0954 1.000
C14 C 0.5879 -0.3087 -0.2674 1.000
C16 C 0.6093 -0.4798 -0.1790 1.000
H19A H -0.0494 -0.3049 -0.1598 1.000
H19B H -0.0095 -0.1758 -0.2180 1.000
C9 C 0.1936 -0.1039 0.5488 1.000
O2 O 0.5075 -0.1853 0.5236 1.000
C13 C 0.6433 0.6347 -0.3487 1.000
H14 H 0.5566 0.7794 -0.2696 1.000
O1 O 0.9047 0.0101 -0.3626 1.000
N1 N 0.7166 0.0957 -0.4644 1.000
C8 C 0.5688 0.1435 -0.4566 1.000
C11 C 0.7502 0.4664 -0.4297 1.000
C4 C 0.5206 0.0805 0.8391 1.000
H5 H 0.2995 0.1617 0.8309 1.000
O1 O 0.9047 0.0101 0.6374 1.000
C3 C 0.6517 0.0477 0.7913 1.000
H16 H 0.5934 0.4758 0.8865 1.000
O4 O 0.5462 0.6480 1.0067 1.000
O5 O 0.5649 0.8336 0.9268 1.000
C2 C 0.6547 1.0739 -0.3058 1.000
H3 H 0.7470 1.0035 -0.1714 1.000
H5 H 0.2995 1.1616 -0.1691 1.000
C6 C 0.4014 1.1623 -0.3070 1.000
C21 C 0.8097 1.4385 0.0954 1.000
O3 O 0.8282 1.3715 0.5829 1.000
N2 N 0.8328 1.4184 0.4290 1.000
C11 C 0.2498 1.5336 0.4297 1.000
C17 C 0.3334 1.5357 0.2600 1.000
H16 H 0.4066 1.5242 0.1135 1.000
H19A H 1.0494 1.3049 0.1598 1.000
H19B H 1.0095 1.1758 0.2180 1.000
N4 N 1.2897 -0.3183 -0.1047 1.000
H18A H 1.3029 -0.1699 -0.1847 1.000
H18B H 1.1871 -0.3073 -0.2555 1.000
C8 C 1.4312 -0.1435 0.4566 1.000
C9 C 1.1936 -0.1039 0.5488 1.000
C7 C 1.4685 -0.1294 0.3540 1.000
H4 H 1.4862 -0.0616 0.0848 1.000

C5 C 1.6011 -0.1368 0.2087 1.000
C3 C 1.6517 0.0477 -0.2087 1.000
H4 H 1.5138 0.0616 -0.0848 1.000
H6 H 1.3058 0.2059 -0.3447 1.000
C7 C 1.5315 0.1294 -0.3540 1.000
H18A H 1.3029 0.8301 -0.1847 1.000
H18B H 1.1871 0.6927 -0.2555 1.000
O4 O 1.5462 0.6480 0.0067 1.000
O5 O 1.5649 0.8336 -0.0732 1.000
C15 C 1.5725 0.6341 -0.1827 1.000
H18A H 1.6971 0.1699 0.1847 1.000
H18B H 1.8129 0.3072 0.2555 1.000
C19 C 1.9568 0.2218 0.1588 1.000
H21A H 1.7330 0.4760 0.0390 1.000
H21B H 1.9267 0.4267 0.0673 1.000
C22 C 1.8615 0.5298 0.1913 1.000
H6 H 1.6942 0.7941 0.3447 1.000
H5 H 1.7005 0.8383 0.1691 1.000
O1 O -0.0953 0.0101 0.6374 1.000
C2 C -0.3453 0.0739 0.6942 1.000
O2 O -0.5075 0.1853 0.4764 1.000
C7 C -0.4685 0.1294 0.6460 1.000
H3 H -0.7470 0.9965 0.1714 1.000
C1 C -0.7775 0.9469 0.3753 1.000
O2 O -0.4925 0.8147 0.5236 1.000
N1 N -0.7166 0.9043 0.4644 1.000
H16 H -0.4066 0.4758 0.8865 1.000
O4 O -0.4538 0.6480 1.0067 1.000
O5 O -0.4351 0.8336 0.9268 1.000
N4 N -0.7103 0.6817 0.8953 1.000
O4 O -0.5462 0.3520 -0.0067 1.000
O5 O -0.5649 0.1664 0.0732 1.000
C15 C -0.5725 0.3659 0.1827 1.000
C12 C -0.3151 1.5218 0.6553 1.000
H4 H -0.4862 1.0616 0.9152 1.000
C5 C -0.6011 1.1368 0.7912 1.000
H6 H -0.6942 1.2059 0.6553 1.000
S2 S -0.5002 1.2937 0.0787 1.000
C21 C -0.1903 1.4385 0.0954 1.000
H18A H 0.3029 -0.1699 -0.1847 1.000
H18B H 0.1871 -0.3073 -0.2555 1.000
H21A H 0.2670 -0.4760 -0.0390 1.000
H21B H 0.0733 -0.4267 -0.0673 1.000
C22 C 0.1385 -0.5298 -0.1913 1.000
C13 C 0.6433 -0.3653 -0.3487 1.000
H14 H 0.5566 -0.2206 -0.2696 1.000
H16 H 0.5934 -0.5242 -0.1135 1.000
C17 C 0.6665 -0.5357 -0.2600 1.000
S1 S 0.0098 -0.2329 0.5281 1.000
H9A H 0.1467 -0.0218 0.5643 1.000
H9B H 0.2845 -0.1107 0.6149 1.000
H13 H 0.6544 0.6779 -0.4153 1.000
C9 C 0.8064 0.1039 -0.5488 1.000

O2 O 0.4925 0.1853 -0.5236 1.000
O3 O 0.8282 0.3715 -0.4171 1.000
N3 N 0.7499 0.4977 -0.5188 1.000
H4 H 0.5138 0.0616 0.9152 1.000
H3 H 0.7470 0.0035 0.8286 1.000
C1 C 0.7775 1.0531 -0.3753 1.000
C7 C 0.5315 1.1294 -0.3540 1.000
H6 H 0.3058 1.2059 -0.3447 1.000
H21A H 0.7330 1.4760 0.0390 1.000
H21B H 0.9267 1.4267 0.0673 1.000
C22 C 0.8615 1.5298 0.1913 1.000
C11 C 0.7502 1.4664 0.5703 1.000
N3 N 0.7499 1.4977 0.4812 1.000
O3 O 0.1718 1.6285 0.4171 1.000
N3 N 0.2501 1.5023 0.5188 1.000
H17 H 0.3029 1.6240 0.2578 1.000
S2 S 1.5002 -0.2937 -0.0788 1.000
C21 C 1.1903 -0.4385 -0.0954 1.000
O2 O 1.5075 -0.1853 0.5236 1.000
S1 S 1.0098 -0.2329 0.5281 1.000
H9A H 1.1467 -0.0218 0.5643 1.000
H9B H 1.2845 -0.1107 0.6149 1.000
C6 C 1.5986 -0.1623 0.3070 1.000
H5 H 1.7005 -0.1617 0.1691 1.000
C2 C 1.6547 0.0739 -0.3058 1.000
H3 H 1.7470 0.0035 -0.1714 1.000
C8 C 1.5688 0.1435 -0.4566 1.000
C14 C 1.5879 0.6913 -0.2674 1.000
C16 C 1.6093 0.5202 -0.1790 1.000
H19A H 2.0494 0.3049 0.1598 1.000
H19B H 2.0095 0.1758 0.2180 1.000
C20 C 1.9367 0.1436 0.0582 1.000
H22A H 1.9492 0.4982 0.2463 1.000
H22B H 1.7471 0.5392 0.2233 1.000
C23 C 1.9500 0.6517 0.1683 1.000
C3 C -0.3483 0.0477 0.7913 1.000
C6 C -0.5986 0.1623 0.6930 1.000
O1 O -0.9047 0.9899 0.3625 1.000
C9 C -0.8064 0.8961 0.5488 1.000
C18 C -0.7883 0.7477 0.8181 1.000
C21 C -0.8097 0.5615 0.9046 1.000
C14 C -0.5879 0.3087 0.2674 1.000
C16 C -0.6093 0.4798 0.1790 1.000
C13 C -0.3567 1.6347 0.6513 1.000
C17 C -0.3335 1.4643 0.7400 1.000
H5 H -0.7005 1.1616 0.8309 1.000
O4 O -0.5462 1.3520 -0.0067 1.000
O5 O -0.5649 1.1664 0.0732 1.000
C15 C -0.5725 1.3659 0.1827 1.000
H21A H -0.2670 1.4760 0.0390 1.000
H21B H -0.0733 1.4267 0.0673 1.000
C22 C -0.1385 1.5298 0.1913 1.000
H22A H 0.0508 -0.4982 -0.2463 1.000

H22B H 0.2529 -0.5392 -0.2232 1.000
C23 C 0.0500 -0.6517 -0.1683 1.000
C12 C 0.6849 -0.4782 -0.3447 1.000
H13 H 0.6544 -0.3221 -0.4153 1.000
H17 H 0.6971 -0.6240 -0.2578 1.000
C10 C 0.1240 -0.3470 0.5092 1.000
S1 S 0.9902 0.2329 -0.5281 1.000
H9A H 0.8533 0.0218 -0.5643 1.000
H9B H 0.7155 0.1107 -0.6149 1.000
C10 C 0.8760 0.3470 -0.5092 1.000
N2 N 0.8328 0.4184 -0.5710 1.000
O1 O 0.9047 1.0101 -0.3626 1.000
N1 N 0.7166 1.0957 -0.4644 1.000
C8 C 0.5688 1.1436 -0.4566 1.000
H22A H 0.9492 1.4982 0.2463 1.000
H22B H 0.7471 1.5392 0.2233 1.000
C23 C 0.9500 1.6517 0.1683 1.000
C12 C 0.6849 1.5218 0.6553 1.000
C10 C 0.1240 1.6530 0.5092 1.000
N2 N 0.1672 1.5816 0.5710 1.000
O4 O 1.5462 -0.3520 0.0067 1.000
O5 O 1.5649 -0.1664 -0.0732 1.000
C15 C 1.5725 -0.3659 -0.1827 1.000
H21A H 1.2670 -0.4760 -0.0390 1.000
H21B H 1.0733 -0.4267 -0.0673 1.000
C22 C 1.1385 -0.5298 -0.1913 1.000
C10 C 1.1240 -0.3470 0.5092 1.000
H6 H 1.6942 -0.2059 0.3447 1.000
C1 C 1.7775 0.0531 -0.3753 1.000
O2 O 1.4925 0.1853 -0.5236 1.000
N1 N 1.7166 0.0957 -0.4644 1.000
C13 C 1.6433 0.6347 -0.3487 1.000
H14 H 1.5566 0.7794 -0.2696 1.000
H16 H 1.5934 0.4758 -0.1135 1.000
C17 C 1.6665 0.4643 -0.2600 1.000
H20A H 1.8877 0.1900 -0.0018 1.000
H20B H 1.8463 0.0595 0.0570 1.000
H20C H 2.0616 0.1253 0.0471 1.000
H23A H 1.8617 0.6833 0.1137 1.000
H23B H 2.0657 0.6423 0.1377 1.000
H23C H 1.9854 0.7160 0.2372 1.000
H3 H -0.2530 0.0035 0.8286 1.000
C4 C -0.4794 0.0805 0.8391 1.000
C5 C -0.6011 0.1368 0.7912 1.000
H6 H -0.6942 0.2059 0.6553 1.000
S1 S -0.9902 0.7671 0.5281 1.000
H9A H -0.8533 0.9782 0.5643 1.000
H9B H -0.7155 0.8893 0.6149 1.000
H18A H -0.6971 0.8301 0.8153 1.000
H18B H -0.8129 0.6927 0.7445 1.000
C19 C -0.9568 0.7782 0.8412 1.000
H21A H -0.7330 0.5240 0.9610 1.000
H21B H -0.9267 0.5733 0.9327 1.000

C22 C -0.8615 0.4702 0.8087 1.000
C13 C -0.6433 0.3653 0.3487 1.000
H14 H -0.5566 0.2206 0.2696 1.000
H16 H -0.5934 0.5242 0.1135 1.000
C17 C -0.6666 0.5357 0.2600 1.000
H13 H -0.3456 1.6779 0.5847 1.000
C14 C -0.4121 1.6913 0.7326 1.000
C16 C -0.3907 1.5202 0.8210 1.000
H17 H -0.3029 1.3760 0.7422 1.000
C14 C -0.5879 1.3087 0.2674 1.000
C16 C -0.6093 1.4798 0.1790 1.000
H22A H -0.0508 1.4982 0.2463 1.000
H22B H -0.2529 1.5392 0.2233 1.000
C23 C -0.0500 1.6517 0.1683 1.000
H23A H 0.1383 -0.6833 -0.1137 1.000
H23B H -0.0657 -0.6423 -0.1377 1.000
H23C H 0.0146 -0.7160 -0.2372 1.000
C11 C 0.7502 -0.5336 -0.4297 1.000
O3 O 0.1718 -0.3715 0.4171 1.000
N2 N 0.1672 -0.4184 0.5710 1.000
C9 C 0.8064 1.1039 -0.5488 1.000
O2 O 0.4925 1.1853 -0.5236 1.000
H23A H 0.8617 1.6833 0.1137 1.000
H23B H 1.0657 1.6423 0.1377 1.000
H23C H 0.9854 1.7160 0.2372 1.000
C13 C 0.6433 1.6347 0.6513 1.000
C17 C 0.6665 1.4643 0.7400 1.000
S1 S 0.0098 1.7671 0.5281 1.000
C14 C 1.5879 -0.3087 -0.2674 1.000
C16 C 1.6093 -0.4798 -0.1790 1.000
H22A H 1.0508 -0.4982 -0.2463 1.000
H22B H 1.2529 -0.5392 -0.2232 1.000
C23 C 1.0500 -0.6517 -0.1683 1.000
O3 O 1.1718 -0.3715 0.4171 1.000
N2 N 1.1672 -0.4184 0.5710 1.000
O1 O 1.9047 0.0101 -0.3626 1.000
C9 C 1.8064 0.1039 -0.5488 1.000
C12 C 1.6849 0.5218 -0.3447 1.000
H13 H 1.6544 0.6779 -0.4153 1.000
H17 H 1.6971 0.3760 -0.2578 1.000
H4 H -0.4862 0.0616 0.9152 1.000
H5 H -0.7005 0.1617 0.8309 1.000
C10 C -0.8760 0.6530 0.5092 1.000
H19A H -1.0494 0.6951 0.8402 1.000
H19B H -1.0095 0.8242 0.7820 1.000
C20 C -0.9367 0.8564 0.9418 1.000
H22A H -0.9492 0.5018 0.7537 1.000
H22B H -0.7471 0.4608 0.7768 1.000
C23 C -0.9500 0.3483 0.8317 1.000
C12 C -0.6849 0.4782 0.3447 1.000
H13 H -0.6544 0.3221 0.4153 1.000
H17 H -0.6971 0.6240 0.2578 1.000
H14 H -0.4434 1.7794 0.7304 1.000

C15 C -0.4275 1.6341 0.8173 1.000
H16 H -0.4066 1.4758 0.8865 1.000
C13 C -0.6433 1.3653 0.3487 1.000
H14 H -0.5566 1.2206 0.2696 1.000
H16 H -0.5934 1.5242 0.1135 1.000
C17 C -0.6666 1.5357 0.2600 1.000
H23A H -0.1383 1.6833 0.1137 1.000
H23B H 0.0657 1.6423 0.1377 1.000
H23C H -0.0146 1.7160 0.2372 1.000
O3 O 0.8282 -0.6285 -0.4171 1.000
N3 N 0.7499 -0.5023 -0.5188 1.000
C11 C 0.2498 -0.4664 0.4297 1.000
N3 N 0.2501 -0.4977 0.5188 1.000
S1 S 0.9902 1.2329 -0.5281 1.000
H9A H 0.8533 1.0218 -0.5643 1.000
H9B H 0.7155 1.1107 -0.6149 1.000
H13 H 0.6544 1.6779 0.5847 1.000
C14 C 0.5879 1.6913 0.7326 1.000
C16 C 0.6093 1.5202 0.8210 1.000
H17 H 0.6971 1.3760 0.7422 1.000
C9 C 0.1936 1.8961 0.5488 1.000
C13 C 1.6433 -0.3653 -0.3487 1.000
H14 H 1.5566 -0.2206 -0.2696 1.000
H16 H 1.5934 -0.5242 -0.1135 1.000
C17 C 1.6665 -0.5357 -0.2600 1.000
H23A H 1.1383 -0.6833 -0.1137 1.000
H23B H 0.9343 -0.6423 -0.1377 1.000
H23C H 1.0146 -0.7160 -0.2372 1.000
C11 C 1.2498 -0.4664 0.4297 1.000
N3 N 1.2501 -0.4977 0.5188 1.000
S1 S 1.9902 0.2329 -0.5281 1.000
H9A H 1.8533 0.0218 -0.5643 1.000
H9B H 1.7155 0.1107 -0.6149 1.000
C11 C 1.7502 0.4664 -0.4297 1.000
O3 O -0.8282 0.6285 0.4171 1.000
N2 N -0.8328 0.5816 0.5710 1.000
H20A H -0.8877 0.8100 1.0018 1.000
H20B H -0.8463 0.9406 0.9430 1.000
H20C H -1.0616 0.8747 0.9529 1.000
H23A H -0.8617 0.3167 0.8863 1.000
H23B H -1.0657 0.3577 0.8623 1.000
H23C H -0.9854 0.2840 0.7628 1.000
C11 C -0.7502 0.5336 0.4297 1.000
S2 S -0.4998 1.7063 0.9212 1.000
C12 C -0.6849 1.4782 0.3447 1.000
H13 H -0.6544 1.3221 0.4153 1.000
H17 H -0.6971 1.6240 0.2578 1.000
C10 C 0.8760 -0.6530 -0.5092 1.000
N2 N 0.8328 -0.5816 -0.5710 1.000
C12 C 0.3151 -0.5218 0.3447 1.000
C10 C 0.8760 1.3470 -0.5092 1.000
H14 H 0.5566 1.7794 0.7304 1.000
C15 C 0.5725 1.6341 0.8173 1.000

H16 H 0.5934 1.4758 0.8865 1.000
N1 N 0.2834 1.9043 0.4644 1.000
H9A H 0.1467 1.9782 0.5643 1.000
H9B H 0.2845 1.8893 0.6149 1.000
C12 C 1.6849 -0.4782 -0.3447 1.000
H13 H 1.6544 -0.3221 -0.4153 1.000
H17 H 1.6971 -0.6240 -0.2578 1.000
C12 C 1.3151 -0.5218 0.3447 1.000
C10 C 1.8760 0.3470 -0.5092 1.000
O3 O 1.8282 0.3715 -0.4171 1.000
N3 N 1.7499 0.4977 -0.5188 1.000
N3 N -0.7499 0.5023 0.5188 1.000
O4 O -0.4538 1.6480 1.0067 1.000
O5 O -0.4351 1.8336 0.9268 1.000
N4 N -0.7103 1.6817 0.8953 1.000
C11 C -0.7502 1.5336 0.4297 1.000
S1 S 0.9902 -0.7671 -0.5281 1.000
C13 C 0.3567 -0.6347 0.3487 1.000
C17 C 0.3334 -0.4643 0.2600 1.000
O3 O 0.8282 1.3715 -0.4171 1.000
N2 N 0.8328 1.4184 -0.5710 1.000
S2 S 0.5002 1.7063 0.9212 1.000
C1 C 0.2225 1.9469 0.3753 1.000
C8 C 0.4312 1.8565 0.4566 1.000
C11 C 1.7502 -0.5336 -0.4297 1.000
C13 C 1.3567 -0.6347 0.3487 1.000
C17 C 1.3334 -0.4643 0.2600 1.000
N2 N 1.8328 0.4184 -0.5710 1.000
C18 C -0.7883 1.7477 0.8181 1.000
C21 C -0.8097 1.5615 0.9046 1.000
O3 O -0.8282 1.6285 0.4171 1.000
N3 N -0.7499 1.5023 0.5188 1.000
C9 C 0.8064 -0.8961 -0.5488 1.000
H13 H 0.3456 -0.6779 0.4153 1.000
C14 C 0.4121 -0.6913 0.2674 1.000
C16 C 0.3907 -0.5202 0.1790 1.000
H17 H 0.3029 -0.3760 0.2578 1.000
C11 C 0.7502 1.4664 -0.4297 1.000
N3 N 0.7499 1.4977 -0.5188 1.000
O4 O 0.5462 1.6480 1.0067 1.000
O5 O 0.5649 1.8336 0.9268 1.000
N4 N 0.2897 1.6817 0.8953 1.000
O1 O 0.0953 1.9899 0.3625 1.000
C2 C 0.3453 1.9261 0.3058 1.000
O2 O 0.5075 1.8147 0.5236 1.000
C7 C 0.4685 1.8706 0.3540 1.000
O3 O 1.8282 -0.6285 -0.4171 1.000
N3 N 1.7499 -0.5023 -0.5188 1.000
H13 H 1.3456 -0.6779 0.4153 1.000
C14 C 1.4121 -0.6913 0.2674 1.000
C16 C 1.3907 -0.5202 0.1790 1.000
H17 H 1.3029 -0.3760 0.2578 1.000
H18A H -0.6971 1.8301 0.8153 1.000

H18B H -0.8129 1.6927 0.7445 1.000
C19 C -0.9568 1.7782 0.8412 1.000
H21A H -0.7330 1.5240 0.9610 1.000
H21B H -0.9267 1.5733 0.9327 1.000
C22 C -0.8615 1.4702 0.8087 1.000
C10 C -0.8760 1.6530 0.5092 1.000
N2 N -0.8328 1.5816 0.5710 1.000
N1 N 0.7166 -0.9043 -0.4644 1.000
H9A H 0.8533 -0.9782 -0.5643 1.000
H9B H 0.7155 -0.8893 -0.6149 1.000
H14 H 0.4434 -0.7794 0.2696 1.000
C15 C 0.4275 -0.6341 0.1827 1.000
H16 H 0.4066 -0.4758 0.1135 1.000
C12 C 0.6849 1.5218 -0.3447 1.000
C18 C 0.2117 1.7477 0.8181 1.000
C21 C 0.1903 1.5615 0.9046 1.000
C3 C 0.3483 1.9523 0.2087 1.000
C6 C 0.5986 1.8377 0.3070 1.000
C10 C 1.8760 -0.6530 -0.5092 1.000
N2 N 1.8328 -0.5816 -0.5710 1.000
H14 H 1.4434 -0.7794 0.2696 1.000
C15 C 1.4275 -0.6341 0.1827 1.000
H16 H 1.4066 -0.4758 0.1135 1.000
H19A H -1.0494 1.6951 0.8402 1.000
H19B H -1.0095 1.8242 0.7820 1.000
C20 C -0.9367 1.8564 0.9418 1.000
H22A H -0.9492 1.5018 0.7537 1.000
H22B H -0.7471 1.4608 0.7768 1.000
C23 C -0.9500 1.3483 0.8317 1.000
S1 S -0.9902 1.7671 0.5281 1.000
C1 C 0.7775 -0.9469 -0.3753 1.000
C8 C 0.5688 -0.8565 -0.4566 1.000
S2 S 0.4998 -0.7063 0.0787 1.000
C13 C 0.6433 1.6347 -0.3487 1.000
C17 C 0.6665 1.4643 -0.2600 1.000
H18A H 0.3029 1.8301 0.8153 1.000
H18B H 0.1871 1.6927 0.7445 1.000
C19 C 0.0432 1.7782 0.8412 1.000
H21A H 0.2670 1.5240 0.9610 1.000
H21B H 0.0733 1.5733 0.9327 1.000
C22 C 0.1385 1.4702 0.8087 1.000
H3 H 0.2530 1.9965 0.1714 1.000
C4 C 0.4794 1.9195 0.1609 1.000
C5 C 0.6011 1.8632 0.2087 1.000
H6 H 0.6942 1.7941 0.3447 1.000
S1 S 1.9902 -0.7671 -0.5281 1.000
S2 S 1.4998 -0.7063 0.0787 1.000
H20A H -0.8877 1.8100 1.0018 1.000
H20B H -0.8463 1.9406 0.9430 1.000
H20C H -1.0616 1.8747 0.9529 1.000
H23A H -0.8617 1.3167 0.8863 1.000
H23B H -1.0657 1.3577 0.8623 1.000
H23C H -0.9854 1.2840 0.7628 1.000

C9 C -0.8064 1.8961 0.5488 1.000
O1 O 0.9047 -0.9899 -0.3626 1.000
C2 C 0.6547 -0.9261 -0.3058 1.000
O2 O 0.4925 -0.8147 -0.5236 1.000
C7 C 0.5315 -0.8706 -0.3540 1.000
O4 O 0.4538 -0.6480 -0.0067 1.000
O5 O 0.4351 -0.8336 0.0732 1.000
N4 N 0.7103 -0.6817 0.1047 1.000
H13 H 0.6544 1.6779 -0.4153 1.000
C14 C 0.5879 1.6913 -0.2674 1.000
C16 C 0.6093 1.5202 -0.1790 1.000
H17 H 0.6971 1.3760 -0.2578 1.000
H19A H -0.0494 1.6951 0.8402 1.000
H19B H -0.0095 1.8242 0.7820 1.000
C20 C 0.0633 1.8564 0.9418 1.000
H22A H 0.0508 1.5018 0.7537 1.000
H22B H 0.2529 1.4608 0.7768 1.000
C23 C 0.0500 1.3483 0.8317 1.000
H4 H 0.4862 1.9384 0.0848 1.000
H5 H 0.7005 1.8384 0.1691 1.000
C9 C 1.8064 -0.8961 -0.5488 1.000
O4 O 1.4538 -0.6480 -0.0067 1.000
O5 O 1.4351 -0.8336 0.0732 1.000
N4 N 1.7103 -0.6817 0.1047 1.000
N1 N -0.7166 1.9043 0.4644 1.000
H9A H -0.8533 1.9782 0.5643 1.000
H9B H -0.7155 1.8893 0.6149 1.000
C3 C 0.6517 -0.9523 -0.2087 1.000
C6 C 0.4014 -0.8377 -0.3070 1.000
C18 C 0.7883 -0.7477 0.1819 1.000
C21 C 0.8097 -0.5615 0.0954 1.000
H14 H 0.5566 1.7794 -0.2696 1.000
C15 C 0.5725 1.6341 -0.1827 1.000
H16 H 0.5934 1.4758 -0.1135 1.000
H20A H 0.1124 1.8100 1.0018 1.000
H20B H 0.1537 1.9406 0.9430 1.000
H20C H -0.0616 1.8747 0.9529 1.000
H23A H 0.1383 1.3167 0.8863 1.000
H23B H -0.0657 1.3577 0.8623 1.000
H23C H 0.0146 1.2840 0.7628 1.000
N1 N 1.7166 -0.9043 -0.4644 1.000
H9A H 1.8533 -0.9782 -0.5643 1.000
H9B H 1.7155 -0.8893 -0.6149 1.000
C18 C 1.7883 -0.7477 0.1819 1.000
C21 C 1.8097 -0.5615 0.0954 1.000
C1 C -0.7775 1.9469 0.3753 1.000
C8 C -0.5688 1.8565 0.4566 1.000
H3 H 0.7470 -0.9965 -0.1714 1.000
C4 C 0.5206 -0.9195 -0.1609 1.000
C5 C 0.3989 -0.8632 -0.2088 1.000
H6 H 0.3058 -0.7941 -0.3447 1.000
H18A H 0.6971 -0.8301 0.1847 1.000
H18B H 0.8129 -0.6927 0.2555 1.000

C19 C 0.9568 -0.7782 0.1588 1.000
H21A H 0.7330 -0.5240 0.0390 1.000
H21B H 0.9267 -0.5733 0.0673 1.000
C22 C 0.8615 -0.4702 0.1913 1.000
S2 S 0.5002 1.7063 -0.0788 1.000
C1 C 1.7775 -0.9469 -0.3753 1.000
C8 C 1.5688 -0.8565 -0.4566 1.000
H18A H 1.6971 -0.8301 0.1847 1.000
H18B H 1.8129 -0.6927 0.2555 1.000
C19 C 1.9568 -0.7782 0.1588 1.000
H21A H 1.7330 -0.5240 0.0390 1.000
H21B H 1.9267 -0.5733 0.0673 1.000
C22 C 1.8615 -0.4702 0.1913 1.000
O1 O -0.9047 1.9899 0.3625 1.000
C2 C -0.6547 1.9261 0.3058 1.000
O2 O -0.4925 1.8147 0.5236 1.000
C7 C -0.5315 1.8706 0.3540 1.000
H4 H 0.5138 -0.9384 -0.0848 1.000
H5 H 0.2995 -0.8383 -0.1691 1.000
H19A H 1.0494 -0.6951 0.1598 1.000
H19B H 1.0095 -0.8242 0.2180 1.000
C20 C 0.9367 -0.8564 0.0582 1.000
H22A H 0.9492 -0.5018 0.2463 1.000
H22B H 0.7471 -0.4608 0.2233 1.000
C23 C 0.9500 -0.3483 0.1683 1.000
O4 O 0.5462 1.6480 0.0067 1.000
O5 O 0.5649 1.8336 -0.0732 1.000
N4 N 0.2897 1.6817 -0.1047 1.000
O1 O 1.9047 -0.9899 -0.3626 1.000
C2 C 1.6547 -0.9261 -0.3058 1.000
O2 O 1.4925 -0.8147 -0.5236 1.000
C7 C 1.5315 -0.8706 -0.3540 1.000
H19A H 2.0494 -0.6951 0.1598 1.000
H19B H 2.0095 -0.8242 0.2180 1.000
C20 C 1.9367 -0.8564 0.0582 1.000
H22A H 1.9492 -0.5018 0.2463 1.000
H22B H 1.7471 -0.4608 0.2233 1.000
C23 C 1.9500 -0.3483 0.1683 1.000
C3 C -0.6517 1.9523 0.2087 1.000
C6 C -0.4014 1.8377 0.3070 1.000
H20A H 0.8877 -0.8100 -0.0018 1.000
H20B H 0.8463 -0.9405 0.0570 1.000
H20C H 1.0616 -0.8747 0.0471 1.000
H23A H 0.8617 -0.3167 0.1137 1.000
H23B H 1.0657 -0.3577 0.1377 1.000
H23C H 0.9854 -0.2840 0.2372 1.000
C18 C 0.2117 1.7477 -0.1819 1.000
C21 C 0.1903 1.5615 -0.0954 1.000
C3 C 1.6517 -0.9523 -0.2087 1.000
C6 C 1.4014 -0.8377 -0.3070 1.000
H20A H 1.8877 -0.8100 -0.0018 1.000
H20B H 1.8463 -0.9405 0.0570 1.000
H20C H 2.0616 -0.8747 0.0471 1.000

H23A H 1.8617 -0.3167 0.1137 1.000
H23B H 2.0657 -0.3577 0.1377 1.000
H23C H 1.9854 -0.2840 0.2372 1.000
H3 H -0.7470 1.9965 0.1714 1.000
C4 C -0.5206 1.9195 0.1609 1.000
C5 C -0.3989 1.8632 0.2087 1.000
H6 H -0.3058 1.7941 0.3447 1.000
H18A H 0.3029 1.8301 -0.1847 1.000
H18B H 0.1871 1.6927 -0.2555 1.000
C19 C 0.0432 1.7782 -0.1588 1.000
H21A H 0.2670 1.5240 -0.0390 1.000
H21B H 0.0733 1.5733 -0.0673 1.000
C22 C 0.1385 1.4702 -0.1913 1.000
H3 H 1.7470 -0.9965 -0.1714 1.000
C4 C 1.5206 -0.9195 -0.1609 1.000
C5 C 1.3989 -0.8632 -0.2088 1.000
H6 H 1.3058 -0.7941 -0.3447 1.000
H4 H -0.5138 1.9384 0.0848 1.000
H5 H -0.2995 1.8384 0.1691 1.000
H19A H -0.0494 1.6951 -0.1598 1.000
H19B H -0.0095 1.8242 -0.2180 1.000
C20 C 0.0633 1.8564 -0.0582 1.000
H22A H 0.0508 1.5018 -0.2463 1.000
H22B H 0.2529 1.4608 -0.2232 1.000
C23 C 0.0500 1.3483 -0.1683 1.000
H4 H 1.5138 -0.9384 -0.0848 1.000
H5 H 1.2995 -0.8383 -0.1691 1.000
H20A H 0.1124 1.8100 0.0018 1.000
H20B H 0.1537 1.9406 -0.0570 1.000
H20C H -0.0616 1.8747 -0.0471 1.000
H23A H 0.1383 1.3167 -0.1137 1.000
H23B H -0.0657 1.3577 -0.1377 1.000
H23C H 0.0146 1.2840 -0.2372 1.000

All Atoms [16 molecules]
928 atoms, fractional coordinates
Label Symbol x y z Occ.

[x, y, z]
H4 H 0.5138 1.0616 -0.0848 1.000
C4 C 0.5206 1.0805 -0.1609 1.000
C3 C 0.6517 1.0477 -0.2087 1.000
C5 C 0.3989 1.1368 -0.2088 1.000
C2 C 0.6547 1.0739 -0.3058 1.000
H3 H 0.7470 1.0035 -0.1714 1.000
H5 H 0.2995 1.1616 -0.1691 1.000
C6 C 0.4014 1.1623 -0.3070 1.000
C1 C 0.7775 1.0531 -0.3753 1.000
C7 C 0.5315 1.1294 -0.3540 1.000
H6 H 0.3058 1.2059 -0.3447 1.000
O1 O 0.9047 1.0101 -0.3626 1.000
N1 N 0.7166 1.0957 -0.4644 1.000

C8 C 0.5688 1.1436 -0.4566 1.000
C9 C 0.8064 1.1039 -0.5488 1.000
O2 O 0.4925 1.1853 -0.5236 1.000
S1 S 0.9902 1.2329 -0.5281 1.000
H9A H 0.8533 1.0218 -0.5643 1.000
H9B H 0.7155 1.1107 -0.6149 1.000
C10 C 0.8760 1.3470 -0.5092 1.000
O3 O 0.8282 1.3715 -0.4171 1.000
N2 N 0.8328 1.4184 -0.5710 1.000
C11 C 0.7502 1.4664 -0.4297 1.000
N3 N 0.7499 1.4977 -0.5188 1.000
C12 C 0.6849 1.5218 -0.3447 1.000
C13 C 0.6433 1.6347 -0.3487 1.000
C17 C 0.6665 1.4643 -0.2600 1.000
H13 H 0.6544 1.6779 -0.4153 1.000
C14 C 0.5879 1.6913 -0.2674 1.000
C16 C 0.6093 1.5202 -0.1790 1.000
H17 H 0.6971 1.3760 -0.2578 1.000
H14 H 0.5566 1.7794 -0.2696 1.000
C15 C 0.5725 1.6341 -0.1827 1.000
H16 H 0.5934 1.4758 -0.1135 1.000
S2 S 0.5002 1.7063 -0.0788 1.000
O4 O 0.5462 1.6480 0.0067 1.000
O5 O 0.5649 1.8336 -0.0732 1.000
N4 N 0.2897 1.6817 -0.1047 1.000
C18 C 0.2117 1.7477 -0.1819 1.000
C21 C 0.1903 1.5615 -0.0954 1.000
H18A H 0.3029 1.8301 -0.1847 1.000
H18B H 0.1871 1.6927 -0.2555 1.000
C19 C 0.0432 1.7782 -0.1588 1.000
H21A H 0.2670 1.5240 -0.0390 1.000
H21B H 0.0733 1.5733 -0.0673 1.000
C22 C 0.1385 1.4702 -0.1913 1.000
H19A H -0.0494 1.6951 -0.1598 1.000
H19B H -0.0095 1.8242 -0.2180 1.000
C20 C 0.0633 1.8564 -0.0582 1.000
H22A H 0.0508 1.5018 -0.2463 1.000
H22B H 0.2529 1.4608 -0.2232 1.000
C23 C 0.0500 1.3483 -0.1683 1.000
H20A H 0.1124 1.8100 0.0018 1.000
H20B H 0.1537 1.9406 -0.0570 1.000
H20C H -0.0616 1.8747 -0.0471 1.000
H23A H 0.1383 1.3167 -0.1137 1.000
H23B H -0.0657 1.3577 -0.1377 1.000
H23C H 0.0146 1.2840 -0.2372 1.000

[x, y, z]
H20C H 0.9384 0.8747 -0.0471 1.000
C20 C 1.0633 0.8564 -0.0582 1.000
H20A H 1.1123 0.8100 0.0018 1.000
C19 C 1.0432 0.7782 -0.1588 1.000
H20B H 1.1537 0.9406 -0.0570 1.000
C18 C 1.2117 0.7477 -0.1819 1.000

H19A H 0.9506 0.6951 -0.1598 1.000
H19B H 0.9905 0.8242 -0.2180 1.000
N4 N 1.2897 0.6817 -0.1047 1.000
H18A H 1.3029 0.8301 -0.1847 1.000
H18B H 1.1871 0.6927 -0.2555 1.000
C21 C 1.1903 0.5615 -0.0954 1.000
S2 S 1.5002 0.7063 -0.0788 1.000
H21A H 1.2670 0.5240 -0.0390 1.000
H21B H 1.0733 0.5733 -0.0673 1.000
C22 C 1.1385 0.4702 -0.1913 1.000
O4 O 1.5462 0.6480 0.0067 1.000
O5 O 1.5649 0.8336 -0.0732 1.000
C15 C 1.5725 0.6341 -0.1827 1.000
C23 C 1.0500 0.3483 -0.1683 1.000
H22A H 1.0508 0.5018 -0.2463 1.000
H22B H 1.2529 0.4608 -0.2232 1.000
C14 C 1.5879 0.6913 -0.2674 1.000
C16 C 1.6093 0.5202 -0.1790 1.000
H23B H 0.9343 0.3577 -0.1377 1.000
H23A H 1.1383 0.3167 -0.1137 1.000
H23C H 1.0146 0.2840 -0.2372 1.000
C13 C 1.6433 0.6347 -0.3487 1.000
H14 H 1.5566 0.7794 -0.2696 1.000
H16 H 1.5934 0.4758 -0.1135 1.000
C17 C 1.6665 0.4643 -0.2600 1.000
C12 C 1.6849 0.5218 -0.3447 1.000
H13 H 1.6544 0.6779 -0.4153 1.000
H17 H 1.6971 0.3760 -0.2578 1.000
C11 C 1.7502 0.4664 -0.4297 1.000
O3 O 1.8282 0.3715 -0.4171 1.000
N3 N 1.7499 0.4977 -0.5188 1.000
C10 C 1.8760 0.3470 -0.5092 1.000
N2 N 1.8328 0.4184 -0.5710 1.000
S1 S 1.9902 0.2329 -0.5281 1.000
C9 C 1.8064 0.1039 -0.5488 1.000
N1 N 1.7166 0.0957 -0.4644 1.000
H9A H 1.8533 0.0218 -0.5643 1.000
H9B H 1.7155 0.1107 -0.6149 1.000
C8 C 1.5688 0.1435 -0.4566 1.000
C1 C 1.7775 0.0531 -0.3753 1.000
C7 C 1.5315 0.1294 -0.3540 1.000
O2 O 1.4925 0.1853 -0.5236 1.000
C2 C 1.6547 0.0739 -0.3058 1.000
O1 O 1.9047 0.0101 -0.3626 1.000
C6 C 1.4014 0.1623 -0.3070 1.000
C3 C 1.6517 0.0477 -0.2087 1.000
C5 C 1.3989 0.1368 -0.2088 1.000
H6 H 1.3058 0.2059 -0.3447 1.000
C4 C 1.5206 0.0805 -0.1609 1.000
H3 H 1.7470 0.0035 -0.1714 1.000
H5 H 1.2995 0.1617 -0.1691 1.000
H4 H 1.5138 0.0616 -0.0848 1.000

[-x, -y, -z]
O4 O 0.4538 0.3520 -0.0067 1.000
S2 S 0.4998 0.2937 0.0787 1.000
O5 O 0.4351 0.1664 0.0732 1.000
N4 N 0.7103 0.3183 0.1047 1.000
C15 C 0.4275 0.3659 0.1827 1.000
C18 C 0.7883 0.2524 0.1819 1.000
C21 C 0.8097 0.4385 0.0954 1.000
C14 C 0.4121 0.3087 0.2674 1.000
C16 C 0.3907 0.4798 0.1790 1.000
H18A H 0.6971 0.1699 0.1847 1.000
H18B H 0.8129 0.3072 0.2555 1.000
C19 C 0.9568 0.2218 0.1588 1.000
H21A H 0.7330 0.4760 0.0390 1.000
H21B H 0.9267 0.4267 0.0673 1.000
C22 C 0.8615 0.5298 0.1913 1.000
C13 C 0.3567 0.3653 0.3487 1.000
H14 H 0.4434 0.2206 0.2696 1.000
H16 H 0.4066 0.5242 0.1135 1.000
C17 C 0.3334 0.5357 0.2600 1.000
C20 C 0.9367 0.1436 0.0582 1.000
H19A H 1.0494 0.3049 0.1598 1.000
H19B H 1.0095 0.1758 0.2180 1.000
H22A H 0.9492 0.4982 0.2463 1.000
H22B H 0.7471 0.5392 0.2233 1.000
C23 C 0.9500 0.6517 0.1683 1.000
C12 C 0.3151 0.4782 0.3447 1.000
H13 H 0.3456 0.3221 0.4153 1.000
H17 H 0.3029 0.6240 0.2578 1.000
H20A H 0.8877 0.1900 -0.0018 1.000
H20B H 0.8463 0.0595 0.0570 1.000
H20C H 1.0616 0.1253 0.0471 1.000
H23A H 0.8617 0.6833 0.1137 1.000
H23C H 0.9854 0.7160 0.2372 1.000
H23B H 1.0657 0.6423 0.1377 1.000
C11 C 0.2498 0.5336 0.4297 1.000
O3 O 0.1718 0.6285 0.4171 1.000
N3 N 0.2501 0.5023 0.5188 1.000
C10 C 0.1240 0.6530 0.5092 1.000
N2 N 0.1672 0.5816 0.5710 1.000
S1 S 0.0098 0.7671 0.5281 1.000
C9 C 0.1936 0.8961 0.5488 1.000
N1 N 0.2834 0.9043 0.4644 1.000
H9A H 0.1467 0.9782 0.5643 1.000
H9B H 0.2845 0.8893 0.6149 1.000
C1 C 0.2225 0.9469 0.3753 1.000
C8 C 0.4312 0.8565 0.4566 1.000
O1 O 0.0953 0.9899 0.3625 1.000
C2 C 0.3453 0.9261 0.3058 1.000
O2 O 0.5075 0.8147 0.5236 1.000
C7 C 0.4685 0.8706 0.3540 1.000
C3 C 0.3483 0.9523 0.2087 1.000
C6 C 0.5986 0.8377 0.3070 1.000

H3 H 0.2530 0.9965 0.1714 1.000
C4 C 0.4794 0.9195 0.1609 1.000
C5 C 0.6011 0.8632 0.2087 1.000
H6 H 0.6942 0.7941 0.3447 1.000
H4 H 0.4862 0.9384 0.0848 1.000
H5 H 0.7005 0.8383 0.1691 1.000

[-x, -y, -z]
C2 C 0.3453 -0.0739 0.3058 1.000
C3 C 0.3483 -0.0477 0.2087 1.000
C1 C 0.2225 -0.0531 0.3753 1.000
C7 C 0.4685 -0.1294 0.3540 1.000
H3 H 0.2530 -0.0035 0.1714 1.000
C4 C 0.4794 -0.0805 0.1609 1.000
O1 O 0.0953 -0.0101 0.3625 1.000
N1 N 0.2834 -0.0957 0.4644 1.000
C6 C 0.5986 -0.1623 0.3070 1.000
C8 C 0.4312 -0.1435 0.4566 1.000
H4 H 0.4862 -0.0616 0.0848 1.000
C5 C 0.6011 -0.1368 0.2087 1.000
C9 C 0.1936 -0.1039 0.5488 1.000
H6 H 0.6942 -0.2059 0.3447 1.000
O2 O 0.5075 -0.1853 0.5236 1.000
H5 H 0.7005 -0.1617 0.1691 1.000
S1 S 0.0098 -0.2329 0.5281 1.000
H9A H 0.1467 -0.0218 0.5643 1.000
H9B H 0.2845 -0.1107 0.6149 1.000
C10 C 0.1240 -0.3470 0.5092 1.000
O3 O 0.1718 -0.3715 0.4171 1.000
N2 N 0.1672 -0.4184 0.5710 1.000
C11 C 0.2498 -0.4664 0.4297 1.000
N3 N 0.2501 -0.4977 0.5188 1.000
C12 C 0.3151 -0.5218 0.3447 1.000
C13 C 0.3567 -0.6347 0.3487 1.000
C17 C 0.3334 -0.4643 0.2600 1.000
H13 H 0.3456 -0.6779 0.4153 1.000
C14 C 0.4121 -0.6913 0.2674 1.000
C16 C 0.3907 -0.5202 0.1790 1.000
H17 H 0.3029 -0.3760 0.2578 1.000
H14 H 0.4434 -0.7794 0.2696 1.000
C15 C 0.4275 -0.6341 0.1827 1.000
H16 H 0.4066 -0.4758 0.1135 1.000
S2 S 0.4998 -0.7063 0.0787 1.000
O4 O 0.4538 -0.6480 -0.0067 1.000
O5 O 0.4351 -0.8336 0.0732 1.000
N4 N 0.7103 -0.6817 0.1047 1.000
C18 C 0.7883 -0.7477 0.1819 1.000
C21 C 0.8097 -0.5615 0.0954 1.000
H18A H 0.6971 -0.8301 0.1847 1.000
H18B H 0.8129 -0.6927 0.2555 1.000
C19 C 0.9568 -0.7782 0.1588 1.000
H21A H 0.7330 -0.5240 0.0390 1.000
H21B H 0.9267 -0.5733 0.0673 1.000

C22 C 0.8615 -0.4702 0.1913 1.000
H19A H 1.0494 -0.6951 0.1598 1.000
H19B H 1.0095 -0.8242 0.2180 1.000
C20 C 0.9367 -0.8564 0.0582 1.000
H22A H 0.9492 -0.5018 0.2463 1.000
H22B H 0.7471 -0.4608 0.2233 1.000
C23 C 0.9500 -0.3483 0.1683 1.000
H20A H 0.8877 -0.8100 -0.0018 1.000
H20B H 0.8463 -0.9405 0.0570 1.000
H20C H 1.0616 -0.8747 0.0471 1.000
H23A H 0.8617 -0.3167 0.1137 1.000
H23B H 1.0657 -0.3577 0.1377 1.000
H23C H 0.9854 -0.2840 0.2372 1.000

[-x, -y, -z]
S1 S 1.0098 0.7671 0.5281 1.000
C10 C 1.1240 0.6530 0.5092 1.000
C9 C 1.1936 0.8961 0.5488 1.000
O3 O 1.1718 0.6285 0.4171 1.000
N2 N 1.1672 0.5816 0.5710 1.000
N1 N 1.2834 0.9043 0.4644 1.000
H9A H 1.1467 0.9782 0.5643 1.000
H9B H 1.2845 0.8893 0.6149 1.000
C11 C 1.2498 0.5336 0.4297 1.000
N3 N 1.2501 0.5023 0.5188 1.000
C1 C 1.2225 0.9469 0.3753 1.000
C8 C 1.4312 0.8565 0.4566 1.000
C12 C 1.3151 0.4782 0.3447 1.000
O1 O 1.0953 0.9899 0.3625 1.000
C2 C 1.3453 0.9261 0.3058 1.000
C7 C 1.4685 0.8706 0.3540 1.000
O2 O 1.5075 0.8147 0.5236 1.000
C13 C 1.3567 0.3653 0.3487 1.000
C17 C 1.3334 0.5357 0.2600 1.000
C3 C 1.3483 0.9523 0.2087 1.000
C6 C 1.5986 0.8377 0.3070 1.000
H13 H 1.3456 0.3221 0.4153 1.000
C14 C 1.4121 0.3087 0.2674 1.000
C16 C 1.3907 0.4798 0.1790 1.000
H17 H 1.3029 0.6240 0.2578 1.000
H3 H 1.2530 0.9965 0.1714 1.000
C4 C 1.4794 0.9195 0.1609 1.000
C5 C 1.6011 0.8632 0.2087 1.000
H6 H 1.6942 0.7941 0.3447 1.000
H14 H 1.4434 0.2206 0.2696 1.000
C15 C 1.4275 0.3659 0.1827 1.000
H16 H 1.4066 0.5242 0.1135 1.000
H4 H 1.4862 0.9384 0.0848 1.000
H5 H 1.7005 0.8383 0.1691 1.000
S2 S 1.4998 0.2937 0.0787 1.000
O5 O 1.4351 0.1664 0.0732 1.000
O4 O 1.4538 0.3520 -0.0067 1.000
N4 N 1.7103 0.3183 0.1047 1.000

C18 C 1.7883 0.2524 0.1819 1.000
C21 C 1.8097 0.4385 0.0954 1.000
H18A H 1.6971 0.1699 0.1847 1.000
H18B H 1.8129 0.3072 0.2555 1.000
C19 C 1.9568 0.2218 0.1588 1.000
H21A H 1.7330 0.4760 0.0390 1.000
H21B H 1.9267 0.4267 0.0673 1.000
C22 C 1.8615 0.5298 0.1913 1.000
H19A H 2.0494 0.3049 0.1598 1.000
H19B H 2.0095 0.1758 0.2180 1.000
C20 C 1.9367 0.1436 0.0582 1.000
H22A H 1.9492 0.4982 0.2463 1.000
H22B H 1.7471 0.5392 0.2233 1.000
C23 C 1.9500 0.6517 0.1683 1.000
H20A H 1.8877 0.1900 -0.0018 1.000
H20B H 1.8463 0.0595 0.0570 1.000
H20C H 2.0616 0.1253 0.0471 1.000
H23A H 1.8617 0.6833 0.1137 1.000
H23B H 2.0657 0.6423 0.1377 1.000
H23C H 1.9854 0.7160 0.2372 1.000

[-x, -y, -z]
O1 O 1.0953 -0.0101 0.3625 1.000
C1 C 1.2225 -0.0531 0.3753 1.000
N1 N 1.2834 -0.0957 0.4644 1.000
C2 C 1.3453 -0.0739 0.3058 1.000
C8 C 1.4312 -0.1435 0.4566 1.000
C9 C 1.1936 -0.1039 0.5488 1.000
C3 C 1.3483 -0.0477 0.2087 1.000
C7 C 1.4685 -0.1294 0.3540 1.000
O2 O 1.5075 -0.1853 0.5236 1.000
S1 S 1.0098 -0.2329 0.5281 1.000
H9A H 1.1467 -0.0218 0.5643 1.000
H9B H 1.2845 -0.1107 0.6149 1.000
H3 H 1.2530 -0.0035 0.1714 1.000
C4 C 1.4794 -0.0805 0.1609 1.000
C6 C 1.5986 -0.1623 0.3070 1.000
C10 C 1.1240 -0.3470 0.5092 1.000
H4 H 1.4862 -0.0616 0.0848 1.000
C5 C 1.6011 -0.1368 0.2087 1.000
H6 H 1.6942 -0.2059 0.3447 1.000
O3 O 1.1718 -0.3715 0.4171 1.000
N2 N 1.1672 -0.4184 0.5710 1.000
H5 H 1.7005 -0.1617 0.1691 1.000
C11 C 1.2498 -0.4664 0.4297 1.000
N3 N 1.2501 -0.4977 0.5188 1.000
C12 C 1.3151 -0.5218 0.3447 1.000
C13 C 1.3567 -0.6347 0.3487 1.000
C17 C 1.3334 -0.4643 0.2600 1.000
H13 H 1.3456 -0.6779 0.4153 1.000
C14 C 1.4121 -0.6913 0.2674 1.000
C16 C 1.3907 -0.5202 0.1790 1.000
H17 H 1.3029 -0.3760 0.2578 1.000

H14 H 1.4434 -0.7794 0.2696 1.000
C15 C 1.4275 -0.6341 0.1827 1.000
H16 H 1.4066 -0.4758 0.1135 1.000
S2 S 1.4998 -0.7063 0.0787 1.000
O4 O 1.4538 -0.6480 -0.0067 1.000
O5 O 1.4351 -0.8336 0.0732 1.000
N4 N 1.7103 -0.6817 0.1047 1.000
C18 C 1.7883 -0.7477 0.1819 1.000
C21 C 1.8097 -0.5615 0.0954 1.000
H18A H 1.6971 -0.8301 0.1847 1.000
H18B H 1.8129 -0.6927 0.2555 1.000
C19 C 1.9568 -0.7782 0.1588 1.000
H21A H 1.7330 -0.5240 0.0390 1.000
H21B H 1.9267 -0.5733 0.0673 1.000
C22 C 1.8615 -0.4702 0.1913 1.000
H19A H 2.0494 -0.6951 0.1598 1.000
H19B H 2.0095 -0.8242 0.2180 1.000
C20 C 1.9367 -0.8564 0.0582 1.000
H22A H 1.9492 -0.5018 0.2463 1.000
H22B H 1.7471 -0.4608 0.2233 1.000
C23 C 1.9500 -0.3483 0.1683 1.000
H20A H 1.8877 -0.8100 -0.0018 1.000
H20B H 1.8463 -0.9405 0.0570 1.000
H20C H 2.0616 -0.8747 0.0471 1.000
H23A H 1.8617 -0.3167 0.1137 1.000
H23B H 2.0657 -0.3577 0.1377 1.000
H23C H 1.9854 -0.2840 0.2372 1.000

[-x, -y, -z]
O5 O 0.4351 1.1664 0.0732 1.000
S2 S 0.4998 1.2937 0.0787 1.000
C15 C 0.4275 1.3659 0.1827 1.000
O4 O 0.4538 1.3520 -0.0067 1.000
N4 N 0.7103 1.3183 0.1047 1.000
C14 C 0.4121 1.3087 0.2674 1.000
C16 C 0.3907 1.4798 0.1790 1.000
C18 C 0.7883 1.2523 0.1819 1.000
C21 C 0.8097 1.4385 0.0954 1.000
H14 H 0.4434 1.2206 0.2696 1.000
C13 C 0.3567 1.3653 0.3487 1.000
C17 C 0.3334 1.5357 0.2600 1.000
H16 H 0.4066 1.5242 0.1135 1.000
H18A H 0.6971 1.1699 0.1847 1.000
H18B H 0.8129 1.3073 0.2555 1.000
C19 C 0.9568 1.2218 0.1588 1.000
H21A H 0.7330 1.4760 0.0390 1.000
H21B H 0.9267 1.4267 0.0673 1.000
C22 C 0.8615 1.5298 0.1913 1.000
C12 C 0.3151 1.4782 0.3447 1.000
H13 H 0.3456 1.3221 0.4153 1.000
H17 H 0.3029 1.6240 0.2578 1.000
C20 C 0.9367 1.1436 0.0582 1.000
H19A H 1.0494 1.3049 0.1598 1.000

H19B H 1.0095 1.1758 0.2180 1.000
H22A H 0.9492 1.4982 0.2463 1.000
H22B H 0.7471 1.5392 0.2233 1.000
C23 C 0.9500 1.6517 0.1683 1.000
C11 C 0.2498 1.5336 0.4297 1.000
H20B H 0.8463 1.0595 0.0570 1.000
H20A H 0.8877 1.1900 -0.0018 1.000
H20C H 1.0616 1.1253 0.0471 1.000
H23A H 0.8617 1.6833 0.1137 1.000
H23B H 1.0657 1.6423 0.1377 1.000
H23C H 0.9854 1.7160 0.2372 1.000
O3 O 0.1718 1.6285 0.4171 1.000
N3 N 0.2501 1.5023 0.5188 1.000
C10 C 0.1240 1.6530 0.5092 1.000
N2 N 0.1672 1.5816 0.5710 1.000
S1 S 0.0098 1.7671 0.5281 1.000
C9 C 0.1936 1.8961 0.5488 1.000
N1 N 0.2834 1.9043 0.4644 1.000
H9A H 0.1467 1.9782 0.5643 1.000
H9B H 0.2845 1.8893 0.6149 1.000
C1 C 0.2225 1.9469 0.3753 1.000
C8 C 0.4312 1.8565 0.4566 1.000
O1 O 0.0953 1.9899 0.3625 1.000
C2 C 0.3453 1.9261 0.3058 1.000
O2 O 0.5075 1.8147 0.5236 1.000
C7 C 0.4685 1.8706 0.3540 1.000
C3 C 0.3483 1.9523 0.2087 1.000
C6 C 0.5986 1.8377 0.3070 1.000
H3 H 0.2530 1.9965 0.1714 1.000
C4 C 0.4794 1.9195 0.1609 1.000
C5 C 0.6011 1.8632 0.2087 1.000
H6 H 0.6942 1.7941 0.3447 1.000
H4 H 0.4862 1.9384 0.0848 1.000
H5 H 0.7005 1.8384 0.1691 1.000

[x, y, z]
O1 O 0.9047 1.0101 0.6374 1.000
C1 C 0.7775 1.0531 0.6247 1.000
N1 N 0.7166 1.0957 0.5356 1.000
C2 C 0.6547 1.0739 0.6942 1.000
C8 C 0.5688 1.1436 0.5434 1.000
C9 C 0.8064 1.1039 0.4512 1.000
C3 C 0.6517 1.0477 0.7913 1.000
C7 C 0.5315 1.1294 0.6460 1.000
O2 O 0.4925 1.1853 0.4764 1.000
H9A H 0.8533 1.0218 0.4357 1.000
H9B H 0.7155 1.1107 0.3851 1.000
S1 S 0.9902 1.2329 0.4719 1.000
C4 C 0.5206 1.0805 0.8391 1.000
H3 H 0.7470 1.0035 0.8286 1.000
C6 C 0.4014 1.1623 0.6930 1.000
C10 C 0.8760 1.3470 0.4908 1.000
C5 C 0.3989 1.1368 0.7912 1.000

H4 H 0.5138 1.0616 0.9152 1.000
H6 H 0.3058 1.2059 0.6553 1.000
O3 O 0.8282 1.3715 0.5829 1.000
N2 N 0.8328 1.4184 0.4290 1.000
H5 H 0.2995 1.1616 0.8309 1.000
C11 C 0.7502 1.4664 0.5703 1.000
N3 N 0.7499 1.4977 0.4812 1.000
C12 C 0.6849 1.5218 0.6553 1.000
C13 C 0.6433 1.6347 0.6513 1.000
C17 C 0.6665 1.4643 0.7400 1.000
H13 H 0.6544 1.6779 0.5847 1.000
C14 C 0.5879 1.6913 0.7326 1.000
C16 C 0.6093 1.5202 0.8210 1.000
H17 H 0.6971 1.3760 0.7422 1.000
H14 H 0.5566 1.7794 0.7304 1.000
C15 C 0.5725 1.6341 0.8173 1.000
H16 H 0.5934 1.4758 0.8865 1.000
S2 S 0.5002 1.7063 0.9212 1.000
O4 O 0.5462 1.6480 1.0067 1.000
O5 O 0.5649 1.8336 0.9268 1.000
N4 N 0.2897 1.6817 0.8953 1.000
C18 C 0.2117 1.7477 0.8181 1.000
C21 C 0.1903 1.5615 0.9046 1.000
H18A H 0.3029 1.8301 0.8153 1.000
H18B H 0.1871 1.6927 0.7445 1.000
C19 C 0.0432 1.7782 0.8412 1.000
H21A H 0.2670 1.5240 0.9610 1.000
H21B H 0.0733 1.5733 0.9327 1.000
C22 C 0.1385 1.4702 0.8087 1.000
H19A H -0.0494 1.6951 0.8402 1.000
H19B H -0.0095 1.8242 0.7820 1.000
C20 C 0.0633 1.8564 0.9418 1.000
H22A H 0.0508 1.5018 0.7537 1.000
H22B H 0.2529 1.4608 0.7768 1.000
C23 C 0.0500 1.3483 0.8317 1.000
H20A H 0.1124 1.8100 1.0018 1.000
H20B H 0.1537 1.9406 0.9430 1.000
H20C H -0.0616 1.8747 0.9529 1.000
H23A H 0.1383 1.3167 0.8863 1.000
H23B H -0.0657 1.3577 0.8623 1.000
H23C H 0.0146 1.2840 0.7628 1.000

[x, y, z]
H19B H -0.0095 0.8242 0.7820 1.000
C19 C 0.0432 0.7782 0.8412 1.000
C18 C 0.2117 0.7477 0.8181 1.000
H19A H -0.0494 0.6951 0.8402 1.000
C20 C 0.0633 0.8564 0.9418 1.000
H18A H 0.3029 0.8301 0.8153 1.000
H18B H 0.1871 0.6927 0.7445 1.000
N4 N 0.2897 0.6817 0.8953 1.000
H20A H 0.1124 0.8100 1.0018 1.000
H20B H 0.1537 0.9406 0.9430 1.000

H20C H -0.0616 0.8747 0.9529 1.000
C21 C 0.1903 0.5615 0.9046 1.000
S2 S 0.5002 0.7063 0.9212 1.000
C22 C 0.1385 0.4702 0.8087 1.000
H21A H 0.2670 0.5240 0.9610 1.000
H21B H 0.0733 0.5733 0.9327 1.000
C15 C 0.5725 0.6341 0.8173 1.000
O4 O 0.5462 0.6480 1.0067 1.000
O5 O 0.5649 0.8336 0.9268 1.000
H22A H 0.0508 0.5018 0.7537 1.000
H22B H 0.2529 0.4608 0.7768 1.000
C23 C 0.0500 0.3483 0.8317 1.000
C14 C 0.5879 0.6913 0.7326 1.000
C16 C 0.6093 0.5202 0.8210 1.000
H23A H 0.1383 0.3167 0.8863 1.000
H23B H -0.0657 0.3577 0.8623 1.000
H23C H 0.0146 0.2840 0.7628 1.000
C13 C 0.6433 0.6347 0.6513 1.000
H14 H 0.5566 0.7794 0.7304 1.000
C17 C 0.6665 0.4643 0.7400 1.000
H16 H 0.5934 0.4758 0.8865 1.000
C12 C 0.6849 0.5218 0.6553 1.000
H13 H 0.6544 0.6779 0.5847 1.000
H17 H 0.6971 0.3760 0.7422 1.000
C11 C 0.7502 0.4664 0.5703 1.000
N3 N 0.7499 0.4977 0.4812 1.000
O3 O 0.8282 0.3715 0.5829 1.000
N2 N 0.8328 0.4184 0.4290 1.000
C10 C 0.8760 0.3470 0.4908 1.000
S1 S 0.9902 0.2329 0.4719 1.000
C9 C 0.8064 0.1039 0.4512 1.000
H9B H 0.7155 0.1107 0.3851 1.000
N1 N 0.7166 0.0957 0.5356 1.000
H9A H 0.8533 0.0218 0.4357 1.000
C8 C 0.5688 0.1435 0.5434 1.000
C1 C 0.7775 0.0531 0.6247 1.000
O2 O 0.4925 0.1853 0.4764 1.000
C7 C 0.5315 0.1294 0.6460 1.000
C2 C 0.6547 0.0739 0.6942 1.000
O1 O 0.9047 0.0101 0.6374 1.000
C6 C 0.4014 0.1623 0.6930 1.000
C3 C 0.6517 0.0477 0.7913 1.000
H6 H 0.3058 0.2059 0.6553 1.000
C5 C 0.3989 0.1368 0.7912 1.000
C4 C 0.5206 0.0805 0.8391 1.000
H3 H 0.7470 0.0035 0.8286 1.000
H5 H 0.2995 0.1617 0.8309 1.000
H4 H 0.5138 0.0616 0.9152 1.000

[-x, -y, -z]
H5 H -0.2995 0.8383 0.1691 1.000
C5 C -0.3989 0.8632 0.2087 1.000
C6 C -0.4014 0.8377 0.3070 1.000

C4 C -0.5206 0.9195 0.1609 1.000
H6 H -0.3058 0.7941 0.3447 1.000
C7 C -0.5315 0.8706 0.3540 1.000
C3 C -0.6517 0.9523 0.2087 1.000
H4 H -0.5138 0.9384 0.0848 1.000
C2 C -0.6547 0.9261 0.3058 1.000
C8 C -0.5688 0.8565 0.4566 1.000
H3 H -0.7470 0.9965 0.1714 1.000
C1 C -0.7775 0.9469 0.3753 1.000
O2 O -0.4925 0.8147 0.5236 1.000
N1 N -0.7166 0.9043 0.4644 1.000
O1 O -0.9047 0.9899 0.3625 1.000
C9 C -0.8064 0.8961 0.5488 1.000
S1 S -0.9902 0.7671 0.5281 1.000
H9A H -0.8533 0.9782 0.5643 1.000
H9B H -0.7155 0.8893 0.6149 1.000
C10 C -0.8760 0.6530 0.5092 1.000
O3 O -0.8282 0.6285 0.4171 1.000
N2 N -0.8328 0.5816 0.5710 1.000
C11 C -0.7502 0.5336 0.4297 1.000
N3 N -0.7499 0.5023 0.5188 1.000
C12 C -0.6849 0.4782 0.3447 1.000
C13 C -0.6433 0.3653 0.3487 1.000
C17 C -0.6666 0.5357 0.2600 1.000
C14 C -0.5879 0.3087 0.2674 1.000
H13 H -0.6544 0.3221 0.4153 1.000
C16 C -0.6093 0.4798 0.1790 1.000
H17 H -0.6971 0.6240 0.2578 1.000
C15 C -0.5725 0.3659 0.1827 1.000
H14 H -0.5566 0.2206 0.2696 1.000
H16 H -0.5934 0.5242 0.1135 1.000
S2 S -0.5002 0.2937 0.0787 1.000
N4 N -0.2897 0.3183 0.1047 1.000
O4 O -0.5462 0.3520 -0.0067 1.000
O5 O -0.5649 0.1664 0.0732 1.000
C18 C -0.2117 0.2524 0.1819 1.000
C21 C -0.1903 0.4385 0.0954 1.000
C19 C -0.0432 0.2218 0.1588 1.000
H18A H -0.3029 0.1699 0.1847 1.000
H18B H -0.1871 0.3072 0.2555 1.000
H21B H -0.0733 0.4267 0.0673 1.000
C22 C -0.1385 0.5298 0.1913 1.000
H21A H -0.2670 0.4760 0.0390 1.000
H19A H 0.0494 0.3049 0.1598 1.000
H19B H 0.0095 0.1758 0.2180 1.000
C20 C -0.0633 0.1436 0.0582 1.000
H22A H -0.0508 0.4982 0.2463 1.000
C23 C -0.0500 0.6517 0.1683 1.000
H22B H -0.2529 0.5392 0.2233 1.000
H20C H 0.0616 0.1253 0.0471 1.000
H20A H -0.1123 0.1900 -0.0018 1.000
H20B H -0.1537 0.0595 0.0570 1.000
H23C H -0.0146 0.7160 0.2372 1.000

H23B H 0.0657 0.6423 0.1377 1.000
H23A H -0.1383 0.6833 0.1137 1.000

[x, y, z]
S1 S -0.0098 0.2329 0.4719 1.000
C10 C -0.1240 0.3470 0.4908 1.000
C9 C -0.1936 0.1039 0.4512 1.000
O3 O -0.1718 0.3715 0.5829 1.000
N2 N -0.1672 0.4184 0.4290 1.000
N1 N -0.2834 0.0957 0.5356 1.000
H9A H -0.1467 0.0218 0.4357 1.000
H9B H -0.2845 0.1107 0.3851 1.000
C11 C -0.2498 0.4664 0.5703 1.000
N3 N -0.2501 0.4977 0.4812 1.000
C1 C -0.2225 0.0531 0.6247 1.000
C8 C -0.4312 0.1435 0.5434 1.000
C12 C -0.3151 0.5218 0.6553 1.000
O1 O -0.0953 0.0101 0.6374 1.000
C2 C -0.3453 0.0739 0.6942 1.000
O2 O -0.5075 0.1853 0.4764 1.000
C7 C -0.4685 0.1294 0.6460 1.000
C13 C -0.3567 0.6347 0.6513 1.000
C17 C -0.3335 0.4643 0.7400 1.000
C3 C -0.3483 0.0477 0.7913 1.000
C6 C -0.5986 0.1623 0.6930 1.000
H13 H -0.3456 0.6779 0.5847 1.000
C14 C -0.4121 0.6913 0.7326 1.000
C16 C -0.3907 0.5202 0.8210 1.000
H17 H -0.3029 0.3760 0.7422 1.000
H3 H -0.2530 0.0035 0.8286 1.000
C4 C -0.4794 0.0805 0.8391 1.000
C5 C -0.6011 0.1368 0.7912 1.000
H6 H -0.6942 0.2059 0.6553 1.000
H14 H -0.4434 0.7794 0.7304 1.000
C15 C -0.4275 0.6341 0.8173 1.000
H16 H -0.4066 0.4758 0.8865 1.000
H4 H -0.4862 0.0616 0.9152 1.000
H5 H -0.7005 0.1617 0.8309 1.000
S2 S -0.4998 0.7063 0.9212 1.000
O4 O -0.4538 0.6480 1.0067 1.000
O5 O -0.4351 0.8336 0.9268 1.000
N4 N -0.7103 0.6817 0.8953 1.000
C18 C -0.7883 0.7477 0.8181 1.000
C21 C -0.8097 0.5615 0.9046 1.000
H18A H -0.6971 0.8301 0.8153 1.000
H18B H -0.8129 0.6927 0.7445 1.000
C19 C -0.9568 0.7782 0.8412 1.000
H21A H -0.7330 0.5240 0.9610 1.000
H21B H -0.9267 0.5733 0.9327 1.000
C22 C -0.8615 0.4702 0.8087 1.000
H19A H -1.0494 0.6951 0.8402 1.000
H19B H -1.0095 0.8242 0.7820 1.000
C20 C -0.9367 0.8564 0.9418 1.000

H22A H -0.9492 0.5018 0.7537 1.000
H22B H -0.7471 0.4608 0.7768 1.000
C23 C -0.9500 0.3483 0.8317 1.000
H20A H -0.8877 0.8100 1.0018 1.000
H20B H -0.8463 0.9406 0.9430 1.000
H20C H -1.0616 0.8747 0.9529 1.000
H23A H -0.8617 0.3167 0.8863 1.000
H23B H -1.0657 0.3577 0.8623 1.000
H23C H -0.9854 0.2840 0.7628 1.000

[x, y, z]
H20C H -0.0616 0.8747 -0.0471 1.000
C20 C 0.0633 0.8564 -0.0582 1.000
H20B H 0.1537 0.9406 -0.0570 1.000
H20A H 0.1124 0.8100 0.0018 1.000
C19 C 0.0432 0.7782 -0.1588 1.000
C18 C 0.2117 0.7477 -0.1819 1.000
H19A H -0.0494 0.6951 -0.1598 1.000
H19B H -0.0095 0.8242 -0.2180 1.000
N4 N 0.2897 0.6817 -0.1047 1.000
H18A H 0.3029 0.8301 -0.1847 1.000
H18B H 0.1871 0.6927 -0.2555 1.000
S2 S 0.5002 0.7063 -0.0788 1.000
C21 C 0.1903 0.5615 -0.0954 1.000
O5 O 0.5649 0.8336 -0.0732 1.000
O4 O 0.5462 0.6480 0.0067 1.000
C15 C 0.5725 0.6341 -0.1827 1.000
H21A H 0.2670 0.5240 -0.0390 1.000
H21B H 0.0733 0.5733 -0.0673 1.000
C22 C 0.1385 0.4702 -0.1913 1.000
C16 C 0.6093 0.5202 -0.1790 1.000
C14 C 0.5879 0.6913 -0.2674 1.000
H22B H 0.2529 0.4608 -0.2232 1.000
C23 C 0.0500 0.3483 -0.1683 1.000
H22A H 0.0508 0.5018 -0.2463 1.000
H16 H 0.5934 0.4758 -0.1135 1.000
C17 C 0.6665 0.4643 -0.2600 1.000
C13 C 0.6433 0.6347 -0.3487 1.000
H14 H 0.5566 0.7794 -0.2696 1.000
H23A H 0.1383 0.3167 -0.1137 1.000
H23B H -0.0657 0.3577 -0.1377 1.000
H23C H 0.0146 0.2840 -0.2372 1.000
C12 C 0.6849 0.5218 -0.3447 1.000
H17 H 0.6971 0.3760 -0.2578 1.000
H13 H 0.6544 0.6779 -0.4153 1.000
C11 C 0.7502 0.4664 -0.4297 1.000
O3 O 0.8282 0.3715 -0.4171 1.000
N3 N 0.7499 0.4977 -0.5188 1.000
C10 C 0.8760 0.3470 -0.5092 1.000
N2 N 0.8328 0.4184 -0.5710 1.000
S1 S 0.9902 0.2329 -0.5281 1.000
C9 C 0.8064 0.1039 -0.5488 1.000
N1 N 0.7166 0.0957 -0.4644 1.000

H9A H 0.8533 0.0218 -0.5643 1.000
H9B H 0.7155 0.1107 -0.6149 1.000
C1 C 0.7775 0.0531 -0.3753 1.000
C8 C 0.5688 0.1435 -0.4566 1.000
C2 C 0.6547 0.0739 -0.3058 1.000
O1 O 0.9047 0.0101 -0.3626 1.000
C7 C 0.5315 0.1294 -0.3540 1.000
O2 O 0.4925 0.1853 -0.5236 1.000
C3 C 0.6517 0.0477 -0.2087 1.000
C6 C 0.4014 0.1623 -0.3070 1.000
H3 H 0.7470 0.0035 -0.1714 1.000
C4 C 0.5206 0.0805 -0.1609 1.000
C5 C 0.3989 0.1368 -0.2088 1.000
H6 H 0.3058 0.2059 -0.3447 1.000
H4 H 0.5138 0.0616 -0.0848 1.000
H5 H 0.2995 0.1617 -0.1691 1.000

[x, y, z]
H20C H 0.9384 -0.1253 -0.0471 1.000
C20 C 1.0633 -0.1436 -0.0582 1.000
H20B H 1.1537 -0.0594 -0.0570 1.000
H20A H 1.1123 -0.1900 0.0018 1.000
C19 C 1.0432 -0.2218 -0.1588 1.000
C18 C 1.2117 -0.2523 -0.1819 1.000
H19A H 0.9506 -0.3049 -0.1598 1.000
H19B H 0.9905 -0.1758 -0.2180 1.000
N4 N 1.2897 -0.3183 -0.1047 1.000
H18A H 1.3029 -0.1699 -0.1847 1.000
H18B H 1.1871 -0.3073 -0.2555 1.000
S2 S 1.5002 -0.2937 -0.0788 1.000
C21 C 1.1903 -0.4385 -0.0954 1.000
O4 O 1.5462 -0.3520 0.0067 1.000
O5 O 1.5649 -0.1664 -0.0732 1.000
C15 C 1.5725 -0.3659 -0.1827 1.000
H21A H 1.2670 -0.4760 -0.0390 1.000
H21B H 1.0733 -0.4267 -0.0673 1.000
C22 C 1.1385 -0.5298 -0.1913 1.000
C14 C 1.5879 -0.3087 -0.2674 1.000
C16 C 1.6093 -0.4798 -0.1790 1.000
H22A H 1.0508 -0.4982 -0.2463 1.000
H22B H 1.2529 -0.5392 -0.2232 1.000
C23 C 1.0500 -0.6517 -0.1683 1.000
C13 C 1.6433 -0.3653 -0.3487 1.000
H14 H 1.5566 -0.2206 -0.2696 1.000
H16 H 1.5934 -0.5242 -0.1135 1.000
C17 C 1.6665 -0.5357 -0.2600 1.000
H23A H 1.1383 -0.6833 -0.1137 1.000
H23B H 0.9343 -0.6423 -0.1377 1.000
H23C H 1.0146 -0.7160 -0.2372 1.000
C12 C 1.6849 -0.4782 -0.3447 1.000
H13 H 1.6544 -0.3221 -0.4153 1.000
H17 H 1.6971 -0.6240 -0.2578 1.000
C11 C 1.7502 -0.5336 -0.4297 1.000

O3 O 1.8282 -0.6285 -0.4171 1.000
N3 N 1.7499 -0.5023 -0.5188 1.000
C10 C 1.8760 -0.6530 -0.5092 1.000
N2 N 1.8328 -0.5816 -0.5710 1.000
S1 S 1.9902 -0.7671 -0.5281 1.000
C9 C 1.8064 -0.8961 -0.5488 1.000
N1 N 1.7166 -0.9043 -0.4644 1.000
H9A H 1.8533 -0.9782 -0.5643 1.000
H9B H 1.7155 -0.8893 -0.6149 1.000
C1 C 1.7775 -0.9469 -0.3753 1.000
C8 C 1.5688 -0.8565 -0.4566 1.000
O1 O 1.9047 -0.9899 -0.3626 1.000
C2 C 1.6547 -0.9261 -0.3058 1.000
O2 O 1.4925 -0.8147 -0.5236 1.000
C7 C 1.5315 -0.8706 -0.3540 1.000
C3 C 1.6517 -0.9523 -0.2087 1.000
C6 C 1.4014 -0.8377 -0.3070 1.000
H3 H 1.7470 -0.9965 -0.1714 1.000
C4 C 1.5206 -0.9195 -0.1609 1.000
C5 C 1.3989 -0.8632 -0.2088 1.000
H6 H 1.3058 -0.7941 -0.3447 1.000
H4 H 1.5138 -0.9384 -0.0848 1.000
H5 H 1.2995 -0.8383 -0.1691 1.000

[x, y, z]
O5 O 0.5649 -0.1664 -0.0732 1.000
S2 S 0.5002 -0.2937 -0.0788 1.000
O4 O 0.5462 -0.3520 0.0067 1.000
N4 N 0.2897 -0.3183 -0.1047 1.000
C15 C 0.5725 -0.3659 -0.1827 1.000
C18 C 0.2117 -0.2523 -0.1819 1.000
C21 C 0.1903 -0.4385 -0.0954 1.000
C14 C 0.5879 -0.3087 -0.2674 1.000
C16 C 0.6093 -0.4798 -0.1790 1.000
C19 C 0.0432 -0.2218 -0.1588 1.000
H18A H 0.3029 -0.1699 -0.1847 1.000
H18B H 0.1871 -0.3073 -0.2555 1.000
H21A H 0.2670 -0.4760 -0.0390 1.000
H21B H 0.0733 -0.4267 -0.0673 1.000
C22 C 0.1385 -0.5298 -0.1913 1.000
C13 C 0.6433 -0.3653 -0.3487 1.000
H14 H 0.5566 -0.2206 -0.2696 1.000
H16 H 0.5934 -0.5242 -0.1135 1.000
C17 C 0.6665 -0.5357 -0.2600 1.000
C20 C 0.0633 -0.1436 -0.0582 1.000
H19A H -0.0494 -0.3049 -0.1598 1.000
H19B H -0.0095 -0.1758 -0.2180 1.000
H22A H 0.0508 -0.4982 -0.2463 1.000
H22B H 0.2529 -0.5392 -0.2232 1.000
C23 C 0.0500 -0.6517 -0.1683 1.000
C12 C 0.6849 -0.4782 -0.3447 1.000
H13 H 0.6544 -0.3221 -0.4153 1.000
H17 H 0.6971 -0.6240 -0.2578 1.000

H20B H 0.1537 -0.0594 -0.0570 1.000
H20A H 0.1124 -0.1900 0.0018 1.000
H20C H -0.0616 -0.1253 -0.0471 1.000
H23A H 0.1383 -0.6833 -0.1137 1.000
H23B H -0.0657 -0.6423 -0.1377 1.000
H23C H 0.0146 -0.7160 -0.2372 1.000
C11 C 0.7502 -0.5336 -0.4297 1.000
O3 O 0.8282 -0.6285 -0.4171 1.000
N3 N 0.7499 -0.5023 -0.5188 1.000
C10 C 0.8760 -0.6530 -0.5092 1.000
N2 N 0.8328 -0.5816 -0.5710 1.000
S1 S 0.9902 -0.7671 -0.5281 1.000
C9 C 0.8064 -0.8961 -0.5488 1.000
N1 N 0.7166 -0.9043 -0.4644 1.000
H9A H 0.8533 -0.9782 -0.5643 1.000
H9B H 0.7155 -0.8893 -0.6149 1.000
C1 C 0.7775 -0.9469 -0.3753 1.000
C8 C 0.5688 -0.8565 -0.4566 1.000
O1 O 0.9047 -0.9899 -0.3626 1.000
C2 C 0.6547 -0.9261 -0.3058 1.000
O2 O 0.4925 -0.8147 -0.5236 1.000
C7 C 0.5315 -0.8706 -0.3540 1.000
C3 C 0.6517 -0.9523 -0.2087 1.000
C6 C 0.4014 -0.8377 -0.3070 1.000
H3 H 0.7470 -0.9965 -0.1714 1.000
C4 C 0.5206 -0.9195 -0.1609 1.000
C5 C 0.3989 -0.8632 -0.2088 1.000
H6 H 0.3058 -0.7941 -0.3447 1.000
H4 H 0.5138 -0.9384 -0.0848 1.000
H5 H 0.2995 -0.8383 -0.1691 1.000

[-x, -y, -z]
C20 C -0.0633 1.1436 0.0582 1.000
H20B H -0.1537 1.0595 0.0570 1.000
H20C H 0.0616 1.1253 0.0471 1.000
C19 C -0.0432 1.2218 0.1588 1.000
H20A H -0.1123 1.1900 -0.0018 1.000
H19B H 0.0095 1.1758 0.2180 1.000
C18 C -0.2117 1.2523 0.1819 1.000
H19A H 0.0494 1.3049 0.1598 1.000
N4 N -0.2897 1.3183 0.1047 1.000
H18A H -0.3029 1.1699 0.1847 1.000
H18B H -0.1871 1.3073 0.2555 1.000
S2 S -0.5002 1.2937 0.0787 1.000
C21 C -0.1903 1.4385 0.0954 1.000
O4 O -0.5462 1.3520 -0.0067 1.000
O5 O -0.5649 1.1664 0.0732 1.000
C15 C -0.5725 1.3659 0.1827 1.000
H21A H -0.2670 1.4760 0.0390 1.000
H21B H -0.0733 1.4267 0.0673 1.000
C22 C -0.1385 1.5298 0.1913 1.000
C14 C -0.5879 1.3087 0.2674 1.000
C16 C -0.6093 1.4798 0.1790 1.000

H22A H -0.0508 1.4982 0.2463 1.000
H22B H -0.2529 1.5392 0.2233 1.000
C23 C -0.0500 1.6517 0.1683 1.000
C13 C -0.6433 1.3653 0.3487 1.000
H14 H -0.5566 1.2206 0.2696 1.000
H16 H -0.5934 1.5242 0.1135 1.000
C17 C -0.6666 1.5357 0.2600 1.000
H23A H -0.1383 1.6833 0.1137 1.000
H23B H 0.0657 1.6423 0.1377 1.000
H23C H -0.0146 1.7160 0.2372 1.000
C12 C -0.6849 1.4782 0.3447 1.000
H13 H -0.6544 1.3221 0.4153 1.000
H17 H -0.6971 1.6240 0.2578 1.000
C11 C -0.7502 1.5336 0.4297 1.000
O3 O -0.8282 1.6285 0.4171 1.000
N3 N -0.7499 1.5023 0.5188 1.000
C10 C -0.8760 1.6530 0.5092 1.000
N2 N -0.8328 1.5816 0.5710 1.000
S1 S -0.9902 1.7671 0.5281 1.000
C9 C -0.8064 1.8961 0.5488 1.000
N1 N -0.7166 1.9043 0.4644 1.000
H9A H -0.8533 1.9782 0.5643 1.000
H9B H -0.7155 1.8893 0.6149 1.000
C1 C -0.7775 1.9469 0.3753 1.000
C8 C -0.5688 1.8565 0.4566 1.000
O1 O -0.9047 1.9899 0.3625 1.000
C2 C -0.6547 1.9261 0.3058 1.000
O2 O -0.4925 1.8147 0.5236 1.000
C7 C -0.5315 1.8706 0.3540 1.000
C3 C -0.6517 1.9523 0.2087 1.000
C6 C -0.4014 1.8377 0.3070 1.000
H3 H -0.7470 1.9965 0.1714 1.000
C4 C -0.5206 1.9195 0.1609 1.000
C5 C -0.3989 1.8632 0.2087 1.000
H6 H -0.3058 1.7941 0.3447 1.000
H4 H -0.5138 1.9384 0.0848 1.000
H5 H -0.2995 1.8384 0.1691 1.000

[x, y, z]
S1 S -0.0098 1.2329 0.4719 1.000
C9 C -0.1936 1.1039 0.4512 1.000
C10 C -0.1240 1.3470 0.4908 1.000
H9A H -0.1467 1.0218 0.4357 1.000
H9B H -0.2845 1.1107 0.3851 1.000
N1 N -0.2834 1.0957 0.5356 1.000
O3 O -0.1718 1.3715 0.5829 1.000
N2 N -0.1672 1.4184 0.4290 1.000
C1 C -0.2225 1.0531 0.6247 1.000
C8 C -0.4312 1.1436 0.5434 1.000
C11 C -0.2498 1.4664 0.5703 1.000
N3 N -0.2501 1.4977 0.4812 1.000
O1 O -0.0953 1.0101 0.6374 1.000
C2 C -0.3453 1.0739 0.6942 1.000

C7 C -0.4685 1.1294 0.6460 1.000
O2 O -0.5075 1.1853 0.4764 1.000
C12 C -0.3151 1.5218 0.6553 1.000
C3 C -0.3483 1.0477 0.7913 1.000
C6 C -0.5986 1.1623 0.6930 1.000
C13 C -0.3567 1.6347 0.6513 1.000
C17 C -0.3335 1.4643 0.7400 1.000
H3 H -0.2530 1.0035 0.8286 1.000
C4 C -0.4794 1.0805 0.8391 1.000
C5 C -0.6011 1.1368 0.7912 1.000
H6 H -0.6942 1.2059 0.6553 1.000
H13 H -0.3456 1.6779 0.5847 1.000
C14 C -0.4121 1.6913 0.7326 1.000
C16 C -0.3907 1.5202 0.8210 1.000
H17 H -0.3029 1.3760 0.7422 1.000
H4 H -0.4862 1.0616 0.9152 1.000
H5 H -0.7005 1.1616 0.8309 1.000
H14 H -0.4434 1.7794 0.7304 1.000
C15 C -0.4275 1.6341 0.8173 1.000
H16 H -0.4066 1.4758 0.8865 1.000
S2 S -0.4998 1.7063 0.9212 1.000
O4 O -0.4538 1.6480 1.0067 1.000
O5 O -0.4351 1.8336 0.9268 1.000
N4 N -0.7103 1.6817 0.8953 1.000
C18 C -0.7883 1.7477 0.8181 1.000
C21 C -0.8097 1.5615 0.9046 1.000
H18A H -0.6971 1.8301 0.8153 1.000
H18B H -0.8129 1.6927 0.7445 1.000
C19 C -0.9568 1.7782 0.8412 1.000
H21A H -0.7330 1.5240 0.9610 1.000
H21B H -0.9267 1.5733 0.9327 1.000
C22 C -0.8615 1.4702 0.8087 1.000
H19A H -1.0494 1.6951 0.8402 1.000
H19B H -1.0095 1.8242 0.7820 1.000
C20 C -0.9367 1.8564 0.9418 1.000
H22A H -0.9492 1.5018 0.7537 1.000
H22B H -0.7471 1.4608 0.7768 1.000
C23 C -0.9500 1.3483 0.8317 1.000
H20A H -0.8877 1.8100 1.0018 1.000
H20B H -0.8463 1.9406 0.9430 1.000
H20C H -1.0616 1.8747 0.9529 1.000
H23A H -0.8617 1.3167 0.8863 1.000
H23B H -1.0657 1.3577 0.8623 1.000
H23C H -0.9854 1.2840 0.7628 1.000
